# Kinetic and Structural Characterization of the Self-Labeling Protein Tags HaloTag7, SNAP-tag, and CLIP-tag

**DOI:** 10.1021/acs.biochem.1c00258

**Published:** 2021-08-02

**Authors:** Jonas Wilhelm, Stefanie Kühn, Miroslaw Tarnawski, Guillaume Gotthard, Jana Tünnermann, Timo Tänzer, Julie Karpenko, Nicole Mertes, Lin Xue, Ulrike Uhrig, Jochen Reinstein, Julien Hiblot, Kai Johnsson

**Affiliations:** † Department of Chemical Biology, Max Planck Institute for Medical Research, 69120 Heidelberg, Germany; ‡ Protein Expression and Characterization Facility, Max Planck Institute for Medical Research, 69120 Heidelberg, Germany; § Structural Biology Group, European Synchrotron Radiation Facility (ESRF), 38043 Grenoble, France; ∥ Institute of Chemical Sciences and Engineering, École Polytechnique Fédérale de Lausanne (EPFL), 1015 Lausanne, Switzerland; ⊥ Chemical Biology Core Facility, European Molecular Biology Laboratory, 69117 Heidelberg, Germany; # Department of Biomolecular Mechanisms, Max Planck Institute for Medical Research, 69120 Heidelberg, Germany

## Abstract

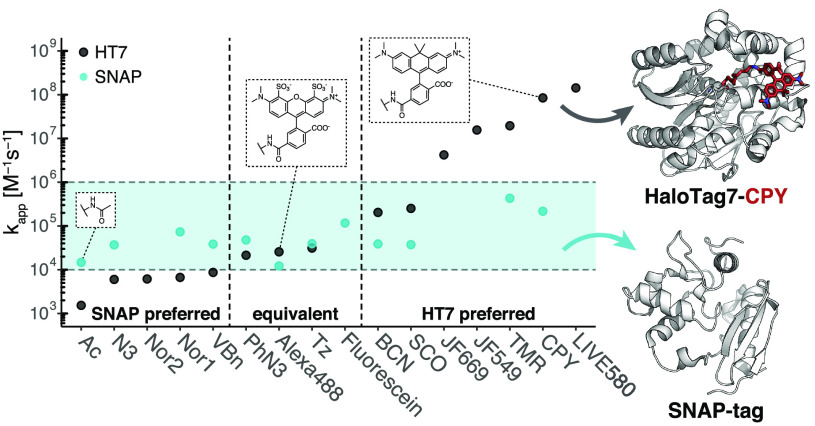

The self-labeling protein tags (SLPs) HaloTag7, SNAP-tag, and CLIP-tag allow the covalent labeling of fusion proteins with synthetic molecules for applications in bioimaging and biotechnology. To guide the selection of an SLP–substrate pair and provide guidelines for the design of substrates, we report a systematic and comparative study of the labeling kinetics and substrate specificities of HaloTag7, SNAP-tag, and CLIP-tag. HaloTag7 reaches almost diffusion-limited labeling rate constants with certain rhodamine substrates, which are more than 2 orders of magnitude higher than those of SNAP-tag for the corresponding substrates. SNAP-tag labeling rate constants, however, are less affected by the structure of the label than those of HaloTag7, which vary over 6 orders of magnitude for commonly employed substrates. Determining the crystal structures of HaloTag7 and SNAP-tag labeled with fluorescent substrates allowed us to rationalize their substrate preferences. We also demonstrate how these insights can be exploited to design substrates with improved labeling kinetics.

Fluorescence imaging techniques require the specific labeling of proteins with appropriate fluorescent probes. Various techniques were developed to visualize proteins of interest (POIs) in living cells, among which the genetic fusion to fluorescent proteins (FPs) remains the most popular approach. However, the brightness and photostability of FPs are inferior to those of organic fluorophores, making the latter attractive for applications in high-resolution fluorescence imaging techniques that require high brightness and photostability.^1^ One approach to coupling organic fluorophores to a POI is through a combination of bio-orthogonal chemistry and the incorporation of unnatural amino acids (UAAs) into POIs.^2−4^ However, applying this approach to live-cell labeling is challenging because of (i) its toxicity,^3^ (ii) the relatively slow labeling reaction (10^–2^ M^–1^ s^–1^ < *k*_2_ < 10^4^ M^–1^ s^–1^), and (iii) putative off-target labeling.^5^ Self-labeling protein tags (SLPs) have been shown to offer a straightforward way to circumvent these issues. They can be genetically fused to POIs and undergo a specific, rapid, and irreversible reaction with their synthetic substrates coupled to bright fluorophores.^6^ SLPs are furthermore employed in various other applications such as *in vitro* biophysical studies,^7,8^ the generation of semisynthetic biosensors,^9−12^ and yeast three-hybrid screenings.^13^ The three most popular SLPs are HaloTag7 (HT7),^14^ SNAP-tag (SNAP),^15^ and CLIP-tag (CLIP)^16^ (Figure 1).

**Figure 1:**
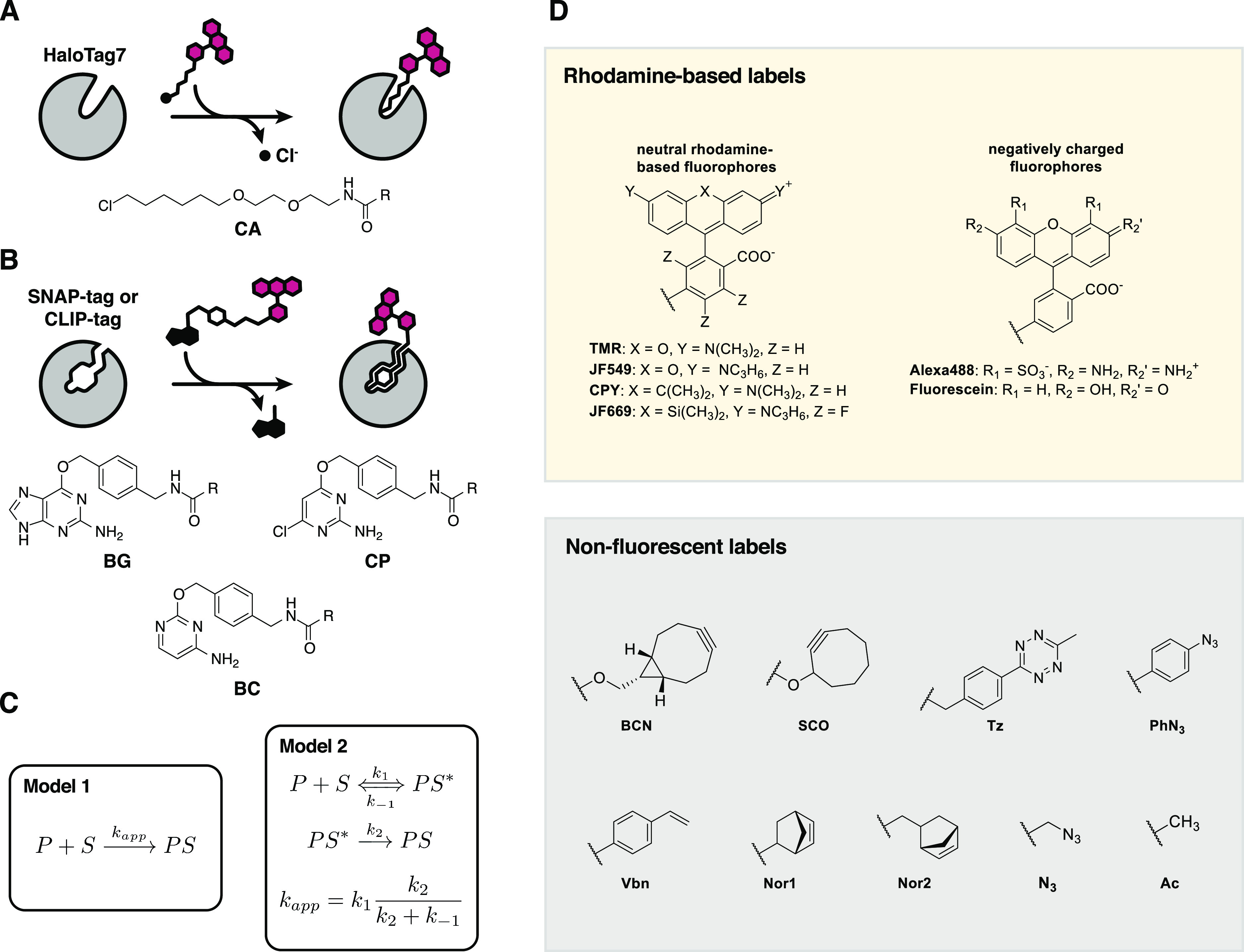
Self-labeling reaction, substrates, and kinetic models. (A) Scheme of the HT7 labeling reaction with fluorophore substrates. The chemical structure of HT7 substrates (CA) is depicted below. R represents the functional moiety to be linked to HT7. (B) Scheme of the SNAP(f)/CLIP(f) labeling reaction with fluorophore substrates. The chemical structures of SNAP/CLIP substrates (BG/CP/BC) are depicted below. R represents the functional moiety to be linked to the SLP. (C) Models employed to describe the SLP kinetics in this study. (D) Popular SLP labels used in this study. Abbreviations: TMR, tetramethylrhodamine; JF, Janelia Fluor dyes; CPY, carbopyronine; BCN, biscyclononyne; SCO, cyclooctyne; Tz, tetrazine; PhN3, phenylazide; Vbn, vinylbenzene; Nor1, (1*R*,4*R*)-bicyclo[2.2.1]hept-2-ene; Nor2, (1*S*,4*S*)-5-methylbicyclo[2.2.1]hept-2-ene; N3, methylazide; Ac, acetate.

HT7 was engineered from a bacterial dehalogenase (DhaA from *Rhodococcus* sp.), an enzyme that can hydrolyze halogenated alkanes.^17^ Inactivating the second catalytic step of its enzymatic reaction (mutation H272N in HT7) abolished the hydrolysis of the ester formed with an active site aspartate residue and created an SLP. HT7 reacts specifically with chloroalkane-PEG (CA) molecules resulting in covalent bonding of the alkane chain to the reactive aspartate and release of a chloride ion (Figure 1A). HT7 was further engineered for increased stability and efficient labeling kinetics toward CA–fluorophore substrates.^18^

SNAP was engineered from the human *O*^6^-alkylguanine-DNA alkyltransferase (hAGT), a protein involved in the repair of alkylated DNA by transferring alkyl moieties to its reactive cysteine.^19^ SNAP was engineered to efficiently react with benzylguanine (BG) derivatives as substrates (Figure 1B) and to reduce its DNA binding properties.^15^ SNAP irreversibly transfers the benzyl moiety of the substrate to its reactive cysteine, leading to the release of guanine. SNAP also accepts substrates in which the guanine is replaced by the more cell-permeable chloropyrimidine (CP)^20^ (Figure 1B). Later, CLIP was engineered from SNAP as an orthogonal SLP system, accepting benzylcytosine (BC) derivatives as substrates^16^ (Figure 1B).

Even though it has become clear over the past several years that the transferred label can have a significant impact on the SLP labeling kinetics,^14,21,22^ no systematic study has yet addressed this point. The structural reasons for the differences in labeling rates are poorly understood. Furthermore, the reaction kinetics of SLPs are usually characterized as a single-step reaction under pseudo-first-order reaction conditions, i.e., in a large excess of one of the reactants (model 1, Figure 1C). We hypothesize that the reaction mechanism of SLPs is more complex and should be characterized by a multistep kinetic model comprising reversible substrate binding (*k*_1_), unbinding (*k*_–1_), and irreversible covalent reaction (*k*_2_) (model 2, Figure 1C).

Here, we report an in-depth characterization of the reaction kinetics of HT7, SNAP, and CLIP with different substrates (Figure 1D), identifying those structural features of labels that control labeling rates for the different tags. We complement these kinetic studies by reporting crystal structures of HT7 and SNAP covalently labeled with rhodamine-based fluorophores, providing a detailed understanding of their substrate preferences. Our results will (i) facilitate the use of SLPs in various applications, (ii) aid in SLP engineering, and (iii) help in the design of improved labeling substrates.

## Experimental Section

### Labeling Substrates and Chemical Synthesis

Labeling substrates for HaloTag, SNAP-tag, and CLIP-tag were synthesized according to literature procedures;^15,16,20,23−32^ were purchased from Promega Corp. (Madison, WI), Abberior GmbH (Göttingen, Germany), Santa Cruz Biotechnology Inc. (Dallas, TX), and NEB Inc. (Ipswich, MA); were kind gifts from L. Lavis (Janelia research campus) and A. D. N. Butkevich (Max Planck Institute for Medical Research); or were synthesized according to the procedure available in the Supporting Information.

### Cloning, Protein Expression, and Purification

SNAP, SNAPf, SNAP^cx^, CLIP, CLIPf, HT7, and HOB were cloned in a pET51b(+) vector (Novagen) for production in *Escherichia coli*, featuring an N-terminal His_10_ tag and a tobacco etch virus (TEV) cleavage site. *Ss*OGT-H^5^ and hAGT were cloned in the same plasmid featuring an N-terminal StrepTag-II and an enterokinase cleavage site together with a C-terminal His_10_ tag. Cloning was performed by Gibson assembly^33^ using E.cloni 10G cells (Lucigen), and point mutations were performed using the Q5 site-directed mutagenesis kit (NEB). Proteins were expressed in *E. coli* strain BL21(DE3)-pLysS (Novagen). Lysogeny broth (LB)^34^ cultures were grown at 37 °C to an optical density at 600 nm (OD_600_) of 0.8. Transgene expression was induced by the addition of 0.5 mM isopropyl β-d-thiogalactopyranoside (IPTG), and cells were grown at 17 °C overnight in the presence of 1 mM MgCl_2_. Cells were harvested by centrifugation and lysed by sonication.

For N-terminally His-tagged proteins, the cell lysate was cleared by centrifugation (75000*g*, 4 °C, 10 min) before affinity-tag purification using a HisTrap FF crude column (Cytiva, Marlborough, MA) and an ÄktaPure FPLC instrument (Cytiva). Buffer was exchanged using a HiPrep 26/10 desalting column (Cytiva) for 50 mM HEPES and 50 mM NaCl (pH 7.3) (i.e., activity buffer). Proteins were concentrated using Ultra 15 mL centrifugal filter devices (Amicon, Merck KGaA, Darmstadt, Germany) with a molecular weight cutoff (MWCO) smaller than the protein size to a final concentration of 500 μM. Proteins were aliquoted and stored at −80 °C after being flash-frozen in liquid nitrogen. Double-tagged proteins, after similar cell lysis and clearing, were purified using HisPur Ni-NTA Superflow Agarose (Thermo Fisher Scientific, Waltham, MA) by batch incubation followed by washing and elution steps on a polypropylene column (Qiagen). Proteins were subsequently purified using a StrepTrap HP column (Cytiva) on an ÄktaPure FPLC instrument. Proteins were then concentrated using Ultra 5 mL centrifugal filter devices with a MWCO smaller than the protein size and conserved in 45% (w/v) glycerol at −20 °C.

The correct size and purity of proteins were assessed by sodium dodecyl sulfate–polyacrylamide gel electrophoresis (SDS–PAGE) and liquid chromatography-mass spectrometry (LC-MS) analysis.

### Affinity of HT7 and HOB for CA Substrates

Binding affinities of HT7^D106A^ or HOB^D106A^ for chloroalkane (CA) substrates were determined by fluorescence polarization (FP, eq 1) measurements using a microplate reader (Spark20M, Tecan Group AG, Männedorf, Switzerland). The fluorescent substrates (10 nM) were titrated against different protein concentrations (0–250 μM) in activity buffer supplemented with 0.5 g/L bovine serum albumin (BSA). Assays were performed in black low-volume nonbinding 384-well plates (Corning Inc., Corning, NY) with a final volume of 20 μL. All measurements were performed in triplicate at 37 °C, and filter settings are listed in Table 1. Obtained FP values were averaged and fitted to a single-site binding model (eq 2) to estimate *K*_d_ values for each fluorescent substrate. The FP value of each dye fully reacted with the native HT7 was used to improve fitting of the upper plateau of the curves by adding an extra data point at a protein concentration of 0.1 M.
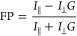
1where FP is the fluorescence polarization, *I*_∥_ is the fluorescence intensity parallel to the excitation light polarization, *I*_⊥_ is the fluorescence intensity perpendicular to the excitation light polarization, and *G* is the grating factor (*G* = *I*_∥_/*I*_⊥_).
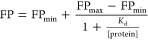
2where FP_min_ is the fluorescence polarization of the free fluorophore (lower plateau), FP_max_ is the maximal fluorescence polarization of fully bound fluorophore (upper plateau), *K*_d_ is the dissociation constant, and [protein] is the protein concentration.

**Table 1:** Filter Settings Used in FP Measurements

fluorophore	excitation filter (BW) (nm)	emission filter (BW) (nm)
Alexa488, fluorescein, Oregon green, JF503, 500R	485 (20)	535 (25)
TMR, JF549, JF525, TMR-az-F2, TMR-CN, TMR-SCH3, TMR-SNH2, MaP555, 510R, 515R, 580CP, Atto565, Atto590, (sulfo-)Cy3	535 (25)	595 (35)
CPY, SiR, LIVE580, JF608, JF646, JF669, (sulfo-)Cy5	620 (20)	680 (30)

### Affinity of SNAP and SNAPf for BG and CP Substrates

Binding affinities of SNAP^C145A^ and SNAPf^C145A^ for BG-Alexa488, CP-Alexa488, BG-fluorescein, CP-fluorescein, BG-MAP555, BG-JF549, BG-TMR(6), BG-TMR(5), CP-TMR, BG-CPY(6), BG-CPY(5), CP-CPY, BG-SiR, CP-SiR, BG-JF646, BG-Atto565, BG-Atto590, BG-sulfo-Cy3, BG-Cy3, BG-sulfo-Cy5, and BG-Cy5 were determined by fluorescence polarization in a manner analogous to that for HT7 affinities for CA substrates described above with the following changes. Fluorescent substrates were titrated at a final concentration of 50 nM against protein concentrations ranging from 0 to 250 μM at room temperature using 0.1 g/L BSA and 1 mM DTT (SNAP-FP buffer). The FP value of each dye fully reacted with the native SNAP/SNAPf was used to improve fitting of the upper plateau of the curves by adding an extra data point at a protein concentration of 0.005 M.

### Affinity of HT7 for Methyl-amide Fluorophores

Binding affinities of HT7 for methyl-amide fluorophores were determined by fluorescence polarization in a manner analogous to that of CA substrates described above with the following changes. Fluorescent substrates were used at a final concentration of 50 nM, and measurements were performed at room temperature.

### Affinity of HT7^D106A^ for CA-Ac Determined via an FP Competition Assay

The binding affinity of HT7^D106A^ for CA-Ac was determined by a fluorescence polarization competition assay against CA-TMR. Therein, 5 μM protein and 50 nM CA-TMR were titrated against CA-Ac concentrations ranging from 80 μM to 10 mM in activity buffer supplemented with 0.5 g/L BSA. Assays were performed in low-volume nonbinding black 384-well plates (Corning Inc.) with a final volume of 20 μL using a microplate reader (Spark20M, Tecan). All measurements were performed in triplicate at 37 °C, and filter settings are listed in Table 1. Obtained FP values were averaged and fitted to a four-parameter logistic curve (eq 3) to estimate the *I*_50_ value. The lower plateau was fixed to the measured FP value of the free dye to improve the fit. The dissociation constant of CA-Ac was calculated as described by Rossi and Taylor.^35^
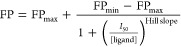
3where FP_min_ is the fluorescence polarization of the free fluorophore (lower plateau), FP_max_ is the maximal fluorescence polarization of the fully bound fluorophore (upper plateau), *I*_50_ is the half-maximal effective concentration, and [ligand] is the ligand concentration.

### Affinity of SNAP^C145A^ for Nonfluorescent Substrates Determined via an FP Competition Assay

Binding affinities of SNAP^C145A^ for BG, CP, BG-Ac, CP-Ac, and BC-Ac were obtained as previously described for HT7 by titrating 5 μM protein and 50 nM CP-TMR against nonfluorescent substrate concentrations ranging from 150 nM to 1.5 mM. Experimental conditions and data analysis were identical despite the fact that 1 mM DTT was added to the buffer and the assay was performed at room temperature.

### Calculation of the Free Binding Energy from *K*_d_

Free binding energies were calculated from *K*_d_ values according to eq 4:
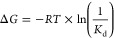
4where Δ*G* is the free binding energy, *R* is the universal gas constant, *T* is the temperature, and *K*_d_ is the dissociation constant.

### HT7 and HOB Labeling Kinetics Determined via Stopped Flow

Labeling kinetics of HT7 with CA-TMR, CA-JF549, CA-CPY, CA-LIVE580, and CA-JF669 and labeling kinetics of HOB with CA-TMR were measured by recording fluorescence anisotropy changes over time using a BioLogic SFM-400 stopped-flow instrument (BioLogic Science Instruments, Claix, France) in a single-mixing configuration at 37 °C. Monochromator wavelengths for excitation and long pass filters used for detection are listed in Table 2. HT7 protein and substrates in activity buffer were mixed in a 1:1 stoichiometry to reach a recordable speed for these fast reactions and to increase the information content of the traces. Concentrations were varied from 0.125 to 1 μM. The anisotropy of the free substrate was measured to obtain a baseline.

**Table 2:** Monochromator Excitation Wavelengths and Filters Used for Stopped-Flow Measurements

fluorophore	excitation wavelength (nm)	emission filter (nm)
TMR/JF549	555	570 Longpass
CPY	610	630 Longpass
LIVE580	603	630 Longpass
JF669	669	690 Longpass

The dead time of the instrument was measured according to the manufacturer’s protocol (BioLogic Technical Note 53) by recording the fluorescence decay during the pseudo-first-order reaction of *N*-acetyl-l-tryptophanamide with a large excess of *N*-bromosuccinimide and fitting the data to the first-order reaction rate law.

### SNAP Labeling Kinetics Determined via Stopped Flow

Labeling kinetics of SNAP with BG-TMR were measured via stopped-flow in a manner analogous to that for HT7 kinetics described above; however, the final substrate concentration was fixed at 2 μM, and the protein concentration was varied from 1.875 to 50 μM. The activity buffer was supplemented with 1 mM DTT.

### HT7 and HOB Labeling Kinetics Determined via a Microplate Reader

Labeling kinetics of HT7 and HOB with CA-Alexa488 were measured by recording FP over time using a microplate reader (Spark20M, Tecan). The final concentration of the fluorophore substrate remained constant (50 nM) with varying protein concentrations (from 200 nM to 256 μM) in activity buffer supplemented with 0.5 g/L BSA. Labeling reactions were started by adding the fluorophore substrate using either multichannel pipets or the injector module of the plate reader. Assays were performed in black nonbinding flat bottom 96-well plates (Corning Inc.) with a final reaction volume of 200 μL. All measurements were performed in triplicate at 37 °C with the filter settings listed in Table 1. The FP of the free substrate was measured to obtain a baseline.

### HT7 Competitive Labeling Kinetics

Competitive kinetics were measured by recording FP over time using a microplate reader (Spark20M, Tecan). The final concentrations of CA-Alexa488 (50 nM) and HT7 protein (200 nM) remained constant with varying concentrations of nonfluorescent substrates (0–1 μM) in activity buffer supplemented with 0.5 g/L BSA. Assays were performed in black nonbinding flat bottom 96-well plates with a final reaction volume of 200 μL. Labeling reactions were started by adding the HT7 protein to wells containing CA-Alexa488 and nonfluorescent substrates using an electronic 96-channel pipettor (Integra Bioscience Corp., Hudson, NH). All measurements were performed in triplicate at 37 °C with the filter settings listed in Table 1. The FP of free CA-Alexa488 was measured to obtain a baseline.

### SNAP and CLIP Labeling Kinetics Determined via a Microplate Reader

Labeling kinetics of SNAP and CLIP substrates were measured by recording FP over time using a microplate reader in a manner analogous to that of HT7 labeling kinetics described above with the following changes. The fluorescent substrate concentration was fixed to 20 nM, and protein concentrations were varied from 15 to 900 nM. Measurements were performed in SNAP-FP buffer. Kinetics with substrates that showed adsorption to plastic were recorded in a black quartz 96-well plate (Hellma GmbH, Müllheim, Germany).

### SNAP Competitive Labeling Kinetics

Competitive kinetics were measured by recording FP over time using a microplate reader in a manner analogous to that for HT7 competition kinetics described above using 100 nM BG-Alexa488 as the fluorescent substrate in SNAP-FP buffer.

### Analysis of Stopped-Flow Data

Kinetic stopped-flow data were preprocessed using a custom R script.^36,37^ Recorded pretrigger time points were removed, and time points were adjusted to start at time zero. Values from replicates were averaged. The anisotropy of the free dye was calculated by averaging anisotropy values of the baseline measurements. Preprocessed data were fitted to a kinetic model (eqs 5 and 6) described by differential eqs 7–10 using the DynaFit software.^38^ The anisotropy of the free dye and the mixing delay of the stopped-flow machine were set as fixed offset and delay parameters in DynaFit. It was assumed that the protein substrate complex and the reacted product are contributing equally to the anisotropy signal. Hence, the response for both species was set equal in DynaFit and fitted together with the kinetic constants. Standard deviations (normal distribution verified) and confidence intervals of fitted parameters were estimated with the Monte Carlo method^39^ with standard settings (*N* = 1000, 5% worst fits discarded). In the case of SNAP kinetics with BG-TMR, the substrate concentration was fitted by DynaFit to rule out quantification errors of the BG-quenched fluorophore. Accurate fitting of the concentration was ensured by including conditions in which protein is limiting and no maximum FP value was reached. Data points and predictions based on the fitted models were plotted using R. Fluorescence intensity changes upon protein binding were verified to be minimal (<12%) and hence not noticeably biasing the fluorescence anisotropy kinetics.
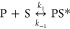
5
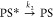
6where P is the SLP protein, S is the SLP substrate, PS* is the protein–substrate complex, and PS is the protein–substrate conjugate.
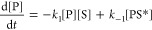
7
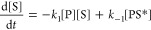
8
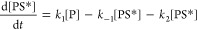
9
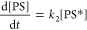
10The derived parameters *K*_d_ (dissociation constant) and *k*_app_ (apparent first-order reaction rate constant) were calculated using the following equations:
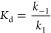
11
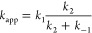
12

### Analysis of Kinetic Microplate Reader Data

Kinetic data from microplate reader assays were fitted to a simplified kinetic model (eq 13) described by differential eqs 14–16 using DynaFit. The dead time of the measurements and baseline FP value were put in as fixed parameters. Standard deviations (normal distribution verified) and confidence intervals of fitted parameters were estimated with the Monte Carlo method with standard settings (*N* = 1000, 5% worst fits discarded). In the case of BG, CP, and BC kinetics, the substrate concentration was fitted by DynaFit to rule out quantification errors of the BG, CP, or BC fluorophores. Accurate fitting of the concentration was ensured by including conditions under which protein is limiting and no maximum FP value was reached. Data points and predictions based on the fitted models were plotted using R.
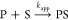
13where P is the SLP protein, S is the SLP substrate, and PS is the protein–substrate conjugate.
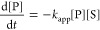
14
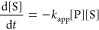
15
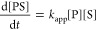
16In some cases, a slow second phase (*k*_3_) was observed in the kinetic data that could not be described by the simplified model (eq 13). These data were fitted to an expanded model that includes a potential conformational change in a second step (eqs 17 and 18).
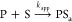
17
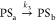
18where P is the SLP protein, S is the SLP substrate, PS_a_ is protein–substrate conjugate state A, and PS_b_ is protein–substrate conjugate state B.

### Analysis of Competition Kinetics

Data were fitted to a simplified kinetic competition model (eqs 19 and 20) described by differential eqs 21–25 using DynaFit. The dead time of the measurements and baseline FP value were put in as fixed parameters. Standard deviations (normal distribution verified) and confidence intervals of fitted parameters were estimated with the Monte Carlo method with standard settings (*N* = 1000, 5% worst fits discarded).
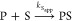
19
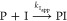
20where P is the SLP protein, S is the fluorescent SLP substrate, I is the nonfluorescent SLP substrate (inhibitor), PS is the protein–fluorescent substrate conjugate, and PI is the protein–nonfluorescent substrate conjugate.
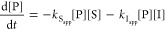
21
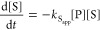
22
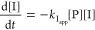
23
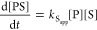
24
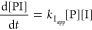
25

### Protein Crystallization

For crystallization trials, protein purification tags were removed by overnight cleavage with TEV protease at 30 °C as previously described.^40^ Cleaved proteins were purified by affinity-tag purification using a HisTrap FF crude column (Cytiva) on an ÄktäPure FPLC instrument, collecting the flow-through. Proteins were further separated by size exclusion chromatography (HiLoad 26/600 Superdex 75, Cytiva) and concentrated using Ultra-4 or 15 mL centrifugal filter devices (Amicon, Merck). The correct size and high purity were verified via SDS–PAGE and LC-MS analysis. Protein labeling was performed in activity buffer, overnight at room temperature using fluorophore substrates at 10 μM (CA-TMR/CA-CPY and BG-TMR for HT7/HOB and SNAP, respectively) in the presence of 5 μM (3 mg) protein. After concentration to ∼200 μL, an excess of fluorophore substrate was removed by buffer exchange using Illustra microspin G-25 columns (Cytiva) according to the manufacturer’s instructions. Protein labeling was verified by SDS–PAGE fluorescence scanning and LC-MS analysis. Protein concentrations were adjusted between 10 and 20 mg/mL and submitted to crystallization trials using different commercial screens via mixing in a 200 nL final volume protein solution/crystallization solution (1:1) using a Mosquito robot (TTP Labtech).

### HT7 Crystal Structures

Crystallization was performed at 20 °C using the vapor diffusion method. Crystals of HT7 labeled with a chloroalkane-PEG-tetramethylrhodamine (CA-TMR) fluorophore substrate were grown by mixing equal volumes of a 20 mg/mL protein solution in 50 mM HEPES (pH 7.3), 50 mM sodium chloride, and a reservoir solution containing 0.1 M MES (pH 6.0), 1.0 M lithium chloride, and 15% (m/v) PEG 6000. The crystals were briefly washed in a cryoprotectant solution consisting of the reservoir solution with glycerol added to a final concentration of 20% (v/v), prior to flash-cooling in liquid nitrogen. Crystals of HT7 labeled with a chloroalkane-PEG-carbopyronine (CA-CPY) fluorophore substrate were obtained by mixing equal volumes of a 15 mg/mL protein solution in 50 mM HEPES (pH 7.3), 50 mM sodium chloride, and a precipitant solution containing 0.1 M Bicine (pH 9.0) and 1.7 M ammonium sulfate. The crystals were briefly washed in a cryoprotectant solution consisting of the reservoir solution supplemented with 20% (v/v) ethylene glycol before flash-cooling in liquid nitrogen. Crystals of HT7-based oligonucleotide binder (HOB) labeled with a CA-TMR fluorophore substrate were grown by mixing equal volumes of a 9.0 mg/mL protein solution in 50 mM HEPES (pH 7.3), 50 mM sodium chloride, and a reservoir solution composed of 0.2 M calcium acetate and 20% (m/v) PEG 3350. Prior to flash-cooling in liquid nitrogen, the crystals were stepwise transferred into a reservoir solution with the PEG 3350 concentration increased to 30% and 40% (m/v).

Single-crystal X-ray diffraction data were collected at 100 K on beamline X10SA at the SLS (Paul Scherrer Institute, Villigen, Switzerland). All data were processed with XDS.^41^ The structure of HT7 labeled with TMR was determined by molecular replacement (MR) using Phaser^42^ and the coordinates of Protein Data Bank (PDB) entry 5UY1 as a search model. The structures of HT7 labeled with CPY and HOB labeled with TMR were subsequently determined by molecular replacement using HT7-TMR as a search model. Geometrical restraints for TMR and CPY were generated using Grade server.^43^ The final models were optimized in iterative cycles of manual rebuilding using Coot^44^ and refinement using Refmac5^45^ and phenix.refine.^46^ Data collection and refinement statistics are summarized in Table S1, and the model quality was validated with MolProbity^47^ as implemented in PHENIX.

### SNAP Crystal Structure

SNAP-TMR crystals were obtained on the crystallography platform of EPFL using the SNAP^cx^-tag construct that features the sequence of SNAP identical to available SNAP crystal structures (PDB entries 3L00, 3KZZ, and 3KZY). Previously crystallized SNAP features the P179R mutation involved in the crystal packing, suggesting its important role for crystallization.^48^ Crystals were obtained under different conditions, including in 100 mM sodium HEPES (pH 7.5) and 25% PEG 8000 from the PEG suite screen (Qiagen) after 48 h at 18 °C. Single crystals were fished and placed in a cryoprotectant solution [containing the crystallization solution supplemented with 20% (v/v) glycerol] before being flash-frozen in liquid nitrogen. Single-crystal X-ray diffraction data were collected on the ID29 beamline at the ESRF (Grenoble, France). Integration, scaling, molecular replacement (using PDB entry 3L00 as a starting model), and refinement were performed as explained for HT7. Refinement statistics are listed in Table S1.

### SNAPf *In Silico* Modeling

The glutamic acid in position 30 of the SNAP-TMR structure (PDB entry 6Y8P) was modeled as an arginine using the mutate function using SYBYL-X1.3 (Tripos International). A side-chain conformation for the arginine was selected from the rotamer source library of Lovell and minimized with few steps with no steric clashes and no direct contact with other positive charges as criteria.

### Structural Analysis

Analysis was conducted on PyMOL.^49^ Root-mean-square deviations (RMSDs) were calculated using the cealign function from PyMOL. Electrostatic potentials were generated using the adaptive Poisson–Boltzmann solver (APBS)^50^ as a PyMOL plugin including the PDB 2PQR software.^51^ Plasmids from this study are available at Addgene (167266–167275).

## Results and Discussion

### Kinetic Characterization of HaloTag7

**Figure 2:**
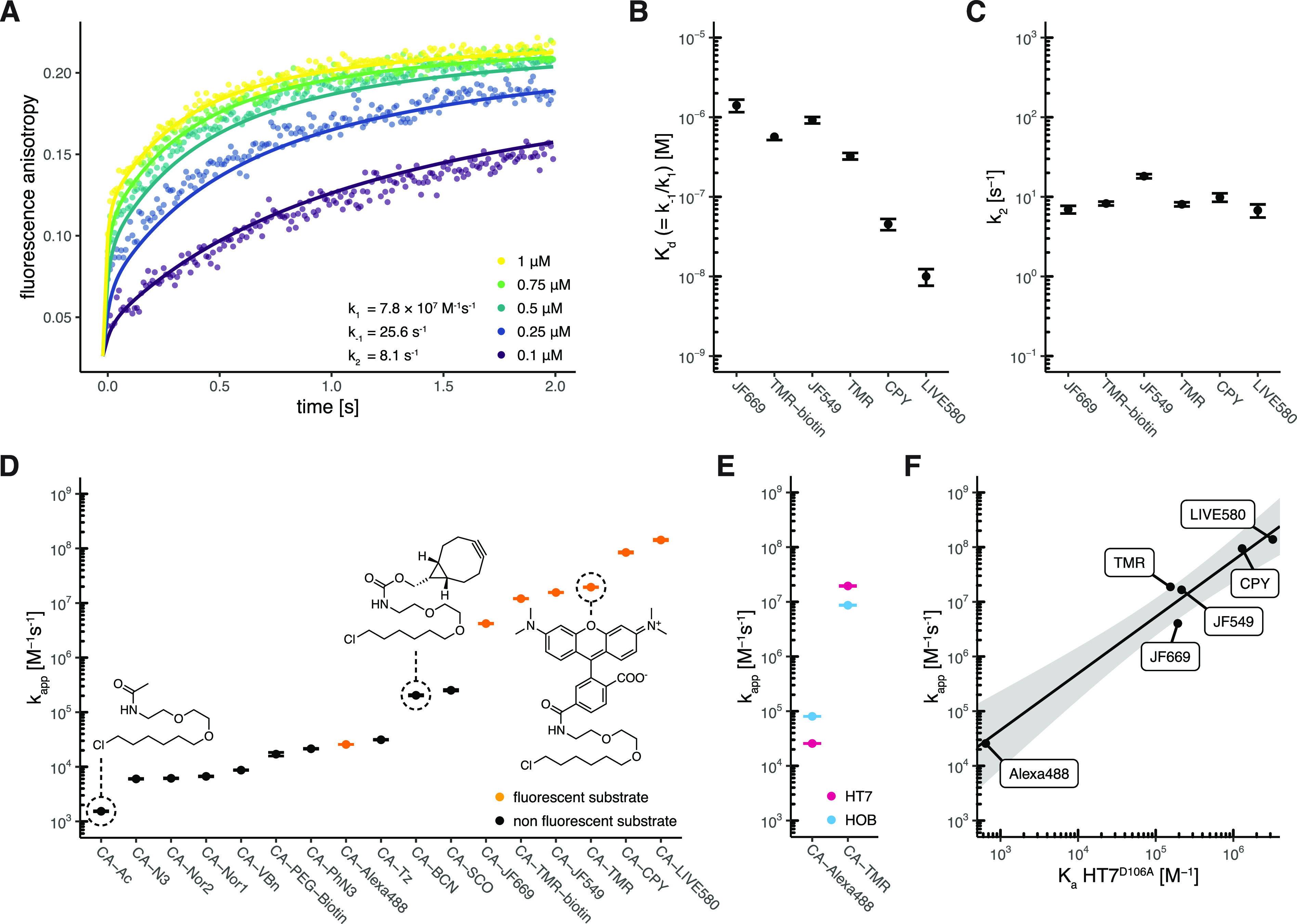
Characterization of HaloTag7 labeling kinetics. (A) Fluorescence anisotropy traces (points) and fitted curves of HT7 labeling with CA-TMR in a 1:1 stoichiometry at the indicated concentrations. Kinetics were recorded by following the fluorescence anisotropy over time using a stopped-flow device. Reactions were started by mixing equal volumes of HT7 and CA-TMR. Data were fitted to kinetic model 2 (lines). (B) HT7 affinities (*K*_d_) for different fluorophore substrates calculated from the kinetic parameters (*k*_–1_ and *k*_1_). (C) HT7 reactivity (*k*_2_) for different fluorophore substrates obtained from fluorescence anisotropy kinetics. The minimal differences in *k*_2_ illustrate that labeling kinetics are mostly influenced by differences in *K*_d_. (D) Apparent second-order labeling rate constants (*k*_app_) of HT7 with different substrates. Rate constants span >6 orders of magnitude. Non-negatively charged fluorophore substrates reach the fastest labeling kinetics. (E) Comparison of *k*_app_ between HT7 and HOB for CA-TMR and CA-Alexa488 labeling highlighting the preference of HOB for the negatively charged substrate CA-Alexa488. (F) Correlation between the HT7 apparent second-order rate constant (*k*_app_) and affinity (*K*_a_ = 1/*K*_d_) for different fluorophore substrates. Affinities were obtained with the inactive variant HT7^D106A^. Log-transformed values were fitted to a linear model [black line, log(*k*_app_) = log(*K*_a_) × 1.042 + 1.544]. The gray area represents the 95% confidence bands (the area in which the true regression line lies with 95% confidence).

Fluorophores represent the most popular class of labels employed with SLPs. We characterized HT7 labeling kinetics with different CA-fluorophore substrates, namely, CA-TMR, CA-JF549, CA-LIVE580, CA-CPY, CA-JF669, and CA-Alexa488 (Figure 1D and Figure S1) by tracking the change in fluorescence anisotropy over time at different reactant concentrations. The very high labeling speed of HT7 toward most rhodamine-based CA substrates required a stopped-flow setup to precisely measure the labeling kinetics. Data were fitted to kinetic model 2 (Figure 1C), which described the reaction kinetics of most rhodamine-based HT7 substrates and allowed the determination of the three kinetic parameters (*k*_1_, *k*_–1_, and *k*_2_) independently (Figure 2A–C, Figure S2, and Table S2). Data fitted to simplified model 1 resulted in a poorer fit, because curves show a clear biphasic character, indicating that model 2 should be preferred to describe these fast labeling kinetics (Figure S3). It should be noted that fitting the data for the faster-reacting substrates to model 1 would lead to a significant overestimation of the labeling speed (Figure S4 and Table S3). The slower labeling reaction with CA-Alexa488 allowed measurements to be carried out in a microplate reader. However, fitting model 2 to these data does not allow determination of the kinetic parameters (*k*_1_, *k*_–1_, and *k*_2_) independently. Hence, the data were fitted using kinetic model 1 (Figure S5). Kinetic model 1 yields the apparent second-order rate constant *k*_app_ that describes the labeling reaction at reactant concentrations far below the *K*_d_ at which the substrate binding site is not saturated and the labeling rate depends linearly on the reactant concentrations. To compare the labeling rate constants of substrates analyzed through different kinetic models (Figure 2D and Table 3), *k*_app_ can also be calculated from the individual rate constants obtained with kinetic model 2 (Figure 1C).

**Table 3:** Apparent Labeling Rate Constants (*k*_app_) for Different HT7, SNAP, and CLIP Substrates

		*k*_app_ (M^–1^ s^–1^) (value ± standard deviation)			
		Halo	SNAP		CLIP
		CA	BG	CP	BC
fluorescent	Alexa488	(2.57 ± 0.01) × 10^4^	(1.22 ± 0.01) × 10^4^	(3.12 ± 0.01) × 10^3^	(1.26 ± 0.01) × 10^3^
	fluorescein	–	(1.17 ± 0.01) × 10^5^	(1.42 ± 0.01) × 10^4^ ^*^	(4.36 ± 0.01) × 10^3^
	JF669	(4.03 ± 0.02) × 10^6^ ^#^	–	–	–
	TMR-biotin	(1.04 ± 0.01) × 10^7^ ^#^	–	–	–
	JF549	(1.66 ± 0.01) × 10^7^ ^#^	–	–	–
	TMR	(1.88 ± 0.01) × 10^7^ ^#^	(4.29 ± 0.01) × 10^5^	(7.69 ± 0.01) × 10^4^	(1.85 ± 0.01) × 10^4^
	CPY	(9.44 ± 0.18) × 10^7^ ^#^	(2.17 ± 0.01) × 10^5^	(1.59 ± 0.01) × 10^4^ ^*^	(2.65 ± 0.01) × 10^4^ ^*^
	Live580	(1.39 ± 0.03) × 10^8^ ^#^	–	–	–
nonfluorescent	Ac	(1.53 ± 0.02) × 10^3^	(1.48 ± 0.05) × 10^4^	(3.45 ± 0.38) × 10^3^	
	–	–	(1.87 ± 0.05) × 10^4^	(4.15 ± 0.62) × 10^3^	
	N_3_	(6.00 ± 0.06) × 10^3^	(3.70 ± 0.09) × 10^4^	(6.36 ± 0.41) × 10^3^	
	Nor2	(6.15 ± 0.07) × 10^3^	–	–	
	Nor1	(6.68 ± 0.06) × 10^3^	(7.34 ± 0.01) × 10^4^	(1.77 ± 0.04) × 10^4^	
	Vbn	(8.68 ± 0.07) × 10^3^	(3.84 ± 0.07) × 10^4^	(5.50 ± 0.45) × 10^3^	
	PEG-biotin	(1.70 ± 0.08) × 10^4^	–	–	
	PhN_3_	(2.14 ± 0.02) × 10^4^	(4.78 ± 0.09) × 10^4^	(2.91 ± 0.40) × 10^3^	
	Tz	(3.13 ± 0.03) × 10^4^	(3.94 ± 0.08) × 10^4^	–	
	BCN	(2.04 ± 0.03) × 10^5^	(3.88 ± 0.07) × 10^4^	(3.34 ± 0.31) × 10^3^	
	SCO	(2.52 ± 0.05) × 10^5^	(3.75 ± 0.06) × 10^4^	(4.22 ± 0.61) × 10^3^	

Rate constants were obtained by fitting the data to kinetic model 1 or using model 2 (#).

For some SNAP and CLIP substrates, a third kinetic model that included a slow aging event of the labeled species (*) was used (see Table S5).

### HaloTag7 Reaches Fast Kinetics with Fluorophore Substrates

Among the tested fluorophore substrates, CA-LIVE580 turned out to be the fastest substrate for HT7 with a *k*_app_ of (1.39 ± 0.03) × 10^8^ M^–1^ s^–1^, reaching an almost diffusion-limited labeling rate, and a calculated *K*_d_ (=*k*_–1_/*k*_1_) of 9.99 nM [7.64–12.35 nM 95% confidence interval (CI)]. All other rhodamine-based substrates showed efficient labeling kinetics, as well (10^6^ M^–1^ s^–1^ < *k*_app_ < 10^9^ M^–1^ s^–1^), with the exception of the negatively charged CA-Alexa488 [*k*_app_ = (2.57 ± 0.01) × 10^4^ M^–1^ s^–1^], which nevertheless presents kinetics equivalent to the fastest click reactions^5^ (Figure 2D and Table 3). The HT7 variant HOB (halo-based oligonucleotide binder)^52^ features several positively charged surface mutations close to the substrate binding site, which were introduced to increase the labeling rates with chloroalkanes attached to oligonucleotides. We hypothesized that HOB may have increased labeling kinetics with the negatively charged CA-Alexa488. Indeed, HOB shows a 3.13 ± 0.01-fold increase in *k*_app_ compared to that of HT7 with CA-Alexa488, while a decrease in *k*_app_ was observed with CA-TMR (2.09 ± 0.01-fold) (Figure 2E, Figure S5, and Table S4). This suggests that kinetics of negatively charged substrates might suffer from charge repulsions at the HT7 surface.

### HaloTag7 Labeling Kinetics Correlate with Substrate Affinity

For the substrates with labeling kinetics that followed model 2, we observed that *k*_1_ and *k*_2_ values were rather constant among the different HT7 fluorophore substrates, while larger differences were observed for dissociation rate constant *k*_–1_ (Figure S6 and Table S2). The substrate preference of HT7 seems therefore mainly driven by the substrate affinity (*K*_d_^kinetic^ = *k*_–1_/*k*_1_) (Figure 2B). After binding, the deeply buried CA moiety might adopt a similar conformation for all substrates, potentially explaining the minor effects of the substituent on the catalytic step (*k*_2_) (Figure 2C). The trend observed for the *K*_d_ values calculated from the kinetic parameters was confirmed by measuring the affinity of inactive variant HT7^D106A^ for the same CA-fluorophore substrates using fluorescence polarization (Figures S6 and S7). *K*_d_^kinetic^ correlates with *K*_d_^D106A^ (Figure S6E), and as a consequence, association constant *K*_a_^D106A^ (=1/*K*_d_) correlates with *k*_app_ (Figure 2F). Hence, *K*_a_^D106A^ can be used to estimate the *k*_app_ for fluorescent HT7 substrates.

### HaloTag7 Reacts Slower with Nonfluorophore Substrates

To determine the *k*_app_ for nonfluorescent CA substrates, we developed a competitive kinetic assay in which the nonfluorescent CA substrates compete with CA-Alexa488 for protein labeling. Nonfluorescent substrates were significantly slower than zwitterionic rhodamine substrates (10^3^ M^–1^ s^–1^ < *k*_app_ < 10^6^ M^–1^ s^–1^), highlighting the strong preference of HT7 for the rhodamine core structure. Larger alkynes (e.g., SCO and BCN) and aromatic structures (e.g., Tz, PhN_3_, and VBn) were preferred over alkenes (Nor) and small moieties (Ac and N_3_) (Figure 2D, Figure S8, and Table 3).

### HaloTag7 Substrate Design

Overall, HT7 can reach labeling kinetics near the diffusion limit, but its apparent rate constants span >6 orders of magnitude, depending on the nature of the label (Figure 2D). HT7 exhibits a strong preference for rhodamine derivatives, with the exception of negatively charged rhodamines. It is noteworthy that the substrate with the slowest labeling rate carries the smallest label, i.e., an acetate group (CA-Ac). The preference for rhodamines can be exploited to increase the labeling rates of poor substrates. As an example, the commercially available CA-PEG-biotin substrate presents slow reaction kinetics [*k*_app_ = (1.70 ± 0.08) × 10^4^ M^–1^ s^–1^ (Table 3 and Figure S8)], but synthesizing a CA-TMR-biotin ligand led to a >500-fold increase in labeling kinetics [*k*_app_ = (1.04 ± 0.01) × 10^7^ M^–1^ s^–1^ (Table 3, Table S1, and Figure S2)], greatly facilitating biotinylation of HT7 fusion proteins. This strategy for improving labeling rates of HT7 ligands should be applicable to various other labels.

### Structural Analysis of Rhodamine-Bound HaloTag

**Figure 3:**
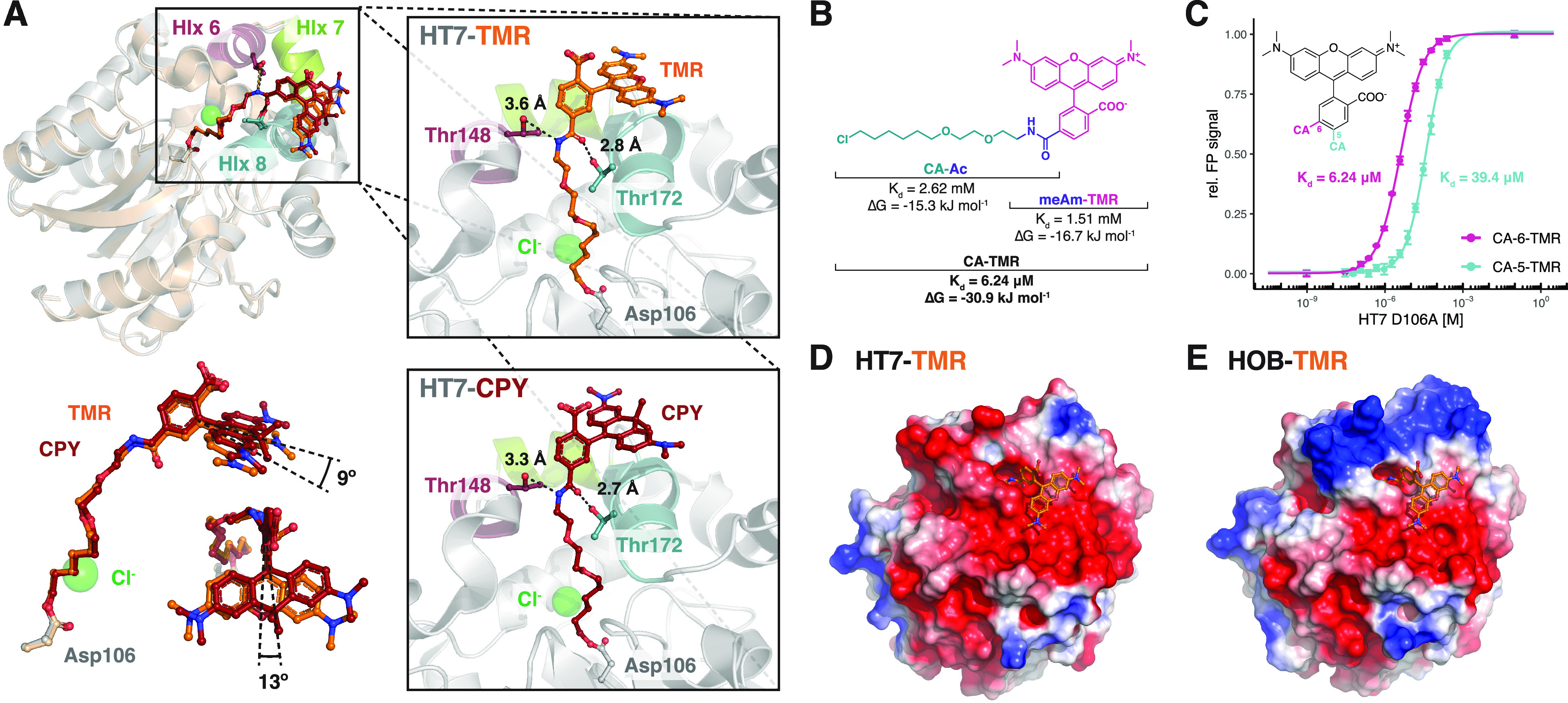
Structure–function analysis of HaloTag7–substrate interactions. (A) Structural comparison between HT7-TMR (PDB entry 6Y7A, gray) and HT7-CPY (PDB entry 6Y7B, chain A, gold). Close-ups of the substrate binding sites of both proteins are included. Proteins are represented as gray cartoons, and the fluorophore substrates and residues as sticks. Putative hydrogen bonds are represented as dashed lines with annotated distances. A comparison of the TMR and CPY conformations on HT7 is shown (bottom left). (B) HT7 affinities (*K*_d_) and free binding energies (Δ*G*) for different TMR substrate substructures. (C) Comparison of HT7 affinity for CA-6-TMR and CA-5-TMR. (D and E) Surface electrostatic potentials of HT7-TMR (PDB entry 6Y7A) and HOB-TMR (PDB entry 6ZCC), respectively. Electrostatic potentials are drawn as protein surfaces from −2.0 (red) to 2.0 (blue) kJ mol^–1^ e^–1^ and were obtained using the APBS software with standard parameters.

To improve our understanding of the substrate preference of HT7 for rhodamine-based CA substrates, we determined the X-ray structure of TMR-bound (PDB entry 6Y7A) and CPY-bound HT7 (PDB entry 6Y7B) at 1.4 and 3.1 Å resolution, respectively (Figure 3A and Table S1). Additionally, the TMR-bound structure of HOB was obtained at 1.5 Å resolution (PDB entry 6ZCC) (Table S1). These structures present the same α/β-hydrolase fold of the superfamily with minimal deviation from already available HT7 X-ray structures^53−56^ (0.26 Å < RMSD^HT7-TMR^ < 0.70 Å over 288 residues with other HT7 structures). In addition to the conventional α/β-hydrolase topology, HT7 features an extra capping domain made of six α-helices (Hlx4–9) that partially cover the catalytic site and form an entry channel for the CA substrate. After reaction, the PEG-alkane ligand is buried in the protein, while the xanthene moiety of the dye lays on the distorted α-helix 8 (Hlx8) in a conformation partially constrained by the crystal packing (Figure 3A and Figure S9A). A recently published HT7-TMR X-ray structure (PDB entry 6U32) shows the fluorophore bound in two alternative conformations.^57^ In one conformation, the fluorophore lays on Hlx8 similar to what we report here, and in the other, it lays on the Hlx7–turn–Hlx8 motif (Figure S9B,C). This second conformation is incompatible with our HT7-TMR structure due to steric clashes caused by crystal packing. The alkane-fluorophore is positioned by the Hlx6–turn–Hlx7–turn–Hlx8 motif of the HT7 capping domain from which T172^Hlx8^ and, to a lesser extent, T148^Hlx6^ form hydrogen bonds with the oxygen and the nitrogen of the amide bond linking PEG-alkane and fluorophore (Figure 3A). CA-TMR and CA-CPY have similar conformations in both structures with only minor differences in their torsion angles (Figure 3A). In comparison to TMR, one of the additional methyl groups of CPY is forming van der Waals interactions at the protein surface, potentially explaining the increased affinity of CA-CPY relative to that of CA-TMR.

### The Fluorophore and CA Core Contribute to HaloTag7 Substrate Affinity

To characterize the contributions of rhodamine structures and the CA core to the overall affinity of HT7 substrates, we measured the affinities of the inactive variant HT7^D106A^ for the acetylated chloroalkane (CA-Ac) and *N*-methylamide-fluorophores (meAm-TMR/CPY). Although the acetylated chloroalkane should form hydrogen bonds to the protein (via T148/T172) and is well buried in the cavity, we observed a rather low affinity (*K*_d_) of 2.62 mM [2.44 to 2.72 mM 95% CI (Figure 3B and Figure S10)], which is consistent with the low apparent labeling rate constant of CA-Ac (Figure 2D). The protein binds the meAm-TMR fluorophore with a slightly higher affinity [*K*_d_ = 1.51 mM, 1.40–1.64 mM 95% CI (Figure 3B and Figure S10)]. The free binding energies for both fragments calculated from the *K*_d_ values (CA-Ac, −15.3 kJ mol^–1^; meAm-TMR, −16.7 kJ mol^–1^) are thus comparable and almost sum to the calculated free binding energy of the full CA-TMR substrate (30.9 kJ mol^–1^; *K*_d_ = 6.24 μM); i.e., no synergistic effect in binding is observed.^58^ Similar results were obtained for meAm-CPY (Figure S10). The CA-fluorophore binding is thus driven by interactions with both the CA core and the fluorophore, explaining the strong impact of changes in the fluorophore structure on the overall labeling kinetics.

The importance of substrate geometry was interrogated by synthesizing CA-fluorophore substrates linked via position 5 of the rhodamine benzyl ring instead of the usual position 6 (Figure 3C). According to the observed conformations in the presented crystal structures, these 5-substrates should not be able to interact with Hlx8 after HT7 binding because the xanthene would be turned 60° away from the protein surface. HT7^D106A^ showed reduced affinities for these substrates compared to the 6-substituted rhodamine substrates (6.31- and 22.7-fold decrease for CA-TMR and CA-CPY, respectively) (Figure 3C). This result emphasizes the importance of the interaction between the xanthene ring and Hlx8.

### The HaloTag7 Surface Charge Impacts Substrate Recognition

HOB comprises four surface mutations compared to HT7 close to the substrate entry channel but opposite the TMR binding site (Figure S9D). These mutations lead to a small but significant increase in labeling rates (3.1-fold) with negatively charged CA substrates relative to HT7. Only minor differences can be observed between the crystal structures of HOB and HT7 labeled with CA-TMR (Figure S9D). Because the HOB mutations replace mostly negatively with positively charged residues, we analyzed the electrostatic potential of both proteins. While HT7 features an overall negatively charged surface around the substrate entry channel (Figure 3D), HOB shows a positively charged patch opposite the fluorophore binding site (Figure 3E). Hence, a putative electrostatic steering effect^59^ could explain the altered substrate preference of HOB despite the fact that its positive charges are on the opposite side of the fluorophore binding site.

### Kinetic Characterization of SNAP-tag

**Figure 4:**
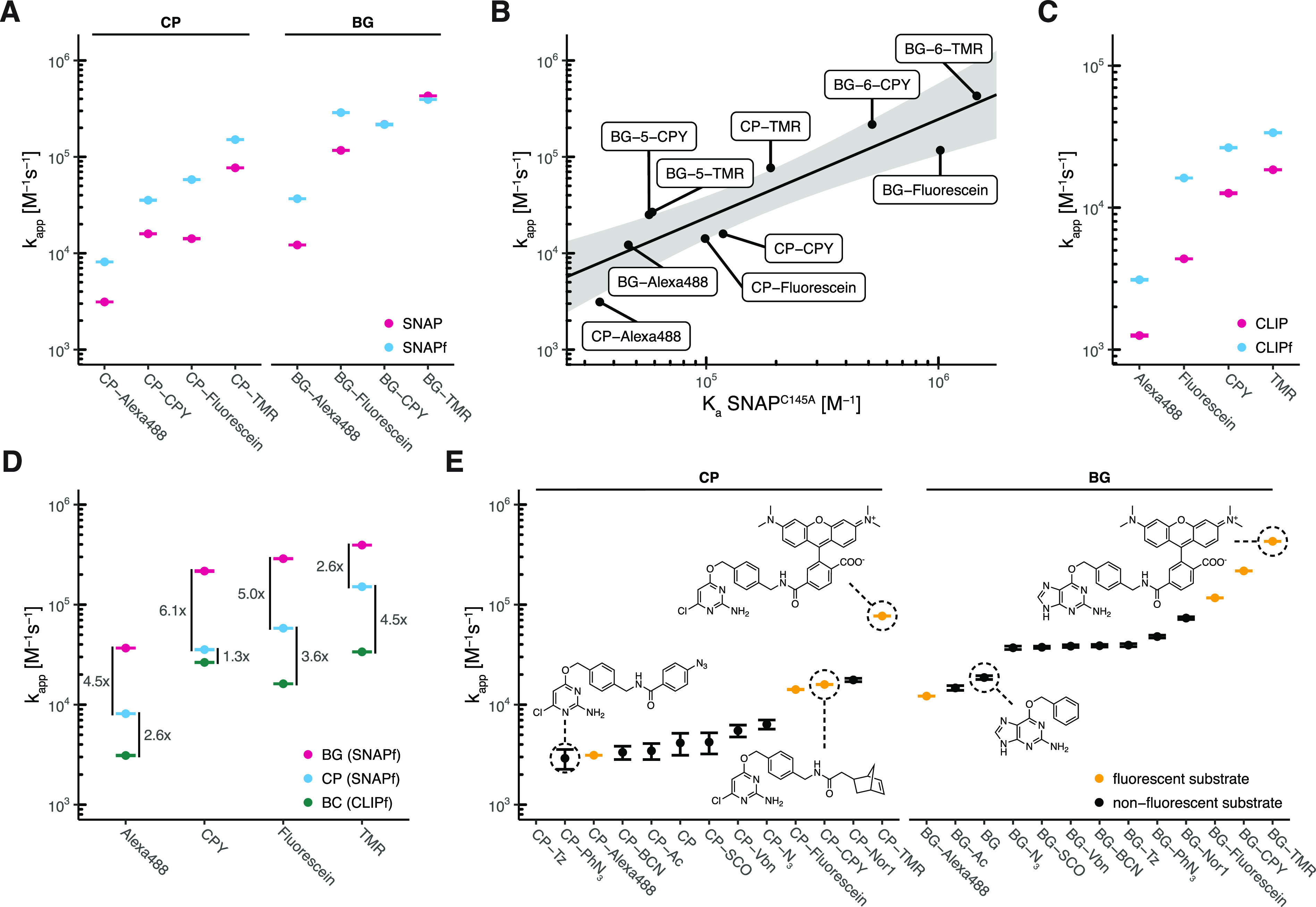
Characterization of SNAP- and CLIP-tag labeling kinetics. (A) Comparison of labeling kinetics (*k*_app_) between SNAP and SNAPf. (B) Correlation between the SNAP apparent second-order rate constant (*k*_app_) and affinity (*K*_a_ = 1/*K*_d_) for different fluorophore substrates. Affinities were obtained for inactive variant SNAP^C145A^. Log-transformed values were fitted to a linear model [black line, log(*k*_app_) = log(*K*_a_) × 1.0217 – 0.7407]. The gray area represents the 95% confidence bands (the area in which the true regression line lies with 95% confidence). (C) Comparison of labeling kinetics (*k*_app_) between CLIP and CLIPf. (D) Comparison of labeling kinetics (*k*_app_) between SNAPf and CLIPf. (E) Apparent second-order labeling rate constants (*k*_app_) of SNAP with different substrates. Kinetics span >3 orders of magnitude (2 orders of magnitude within each substrate class BG/CP). BG-based, non-negatively charged fluorophore substrates reach the fastest labeling kinetics.

SNAP labeling kinetics were characterized for both BG- and CP-fluorophore substrates (i.e., TMR, CPY, Alexa488, and fluorescein) (Figure 1D and Figure S1), by following fluorescence polarization changes during the labeling reaction at different protein concentrations in a plate reader assay. Kinetic model 2 did not allow determination of the kinetic parameters (*k*_1_, *k*_–1_, and *k*_2_) independently. Hence, data were fitted to model 1 to obtain apparent second-order rate constants (*k*_app_) of the labeling reactions (Table 3 and Figure S11). SNAP’s apparent labeling rate constants range between 10^4^ and 10^6^ M^–1^ s^–1^ for BG-fluorophore substrates (Figure 4A), among which BG-TMR presents the fastest labeling rate [*k*_app_ = (4.29 ± 0.01) × 10^5^ M^–1^ s^–1^ (Table 3)]. CP substrates show 4–14-fold slower reaction kinetics than the corresponding BG substrates (10^3^ M^–1^ s^–1^ < *k*_app_ < 10^5^ M^–1^ s^–1^) (Figure 4A). Some CP substrates (CPY and fluorescein) exhibit a slow additional phase of a decrease or increase in fluorescence polarization after labeling that might be due to a slow conformational change of the labeled protein. To fit these traces, kinetic model 1 was extended by adding a step that occurs after labeling. The rate constants of this additional process (*k*_3_) ranged between 10^–2^ and 10^–3^ s^–1^ (Figure S11 and Table S5). SNAP labeling with BG-TMR and CP-TMR was further investigated by measuring stopped-flow fluorescence anisotropy kinetics at higher protein concentrations (Figure S12 and Table S6). Fitting the data to kinetic model 2 allowed estimation of kinetic parameters *k*_1_, *k*_–1_, and *k*_2_ independently and calculation of *K*_d_ values (Figure S12C). The calculated *k*_app_ values for both substrates were similar to the *k*_app_ determined via the plate reader assay using model 1 (Figure S12C). CP-TMR presents *k*_1_ and *k*_2_ values similar to those of BG-TMR, while *k*_–1_ is significantly higher for CP-TMR (8.8-fold), indicating that both substrates feature the same reactivity but differ in their affinity for SNAP.

### SNAP-tag Labeling Kinetics Correlate with Substrate Affinity

To confirm the previous finding, affinities for different fluorescent substrates were measured using inactive variant SNAP^C145A^ (Figure S13). A strong preference for BG-TMR over CP-TMR was observed with an ∼1 order of magnitude difference in *K*_d_^C145A^. SNAP^C145A^ presents a 3-fold lower *K*_d_^C145A^ for BG-TMR (0.68 μM, 0.63–0.75 μM 95% CI) compared to that calculated from stopped-flow experiments using active SNAP. SNAP^C145A^ showed affinities similar to those of BG-TMR toward various xanthene-based fluorophores such as BG-MaP555, BG-JF549, and BG-fluorescein (Figure S13), indicating that modifications of the rhodamine structure seem not to affect the affinity of the protein as much as observed for HT7 substrates. However, SNAP^C145A^ has a very low affinity for sulfonated fluorophore substrates such as BG-Alexa488 (21.6 μM, 20.5–22.9 μM 95% CI) or BG-sulfo-Cy3/5 (Cy3, 68.1 μM; 63.8–72.7 μM 95% CI) (Figure S13). A good correlation between *K*_d_^C145A^ and *k*_app_ was observed for the tested fluorophore substrates (Figure 4B), highlighting again the importance of high affinity for a quick labeling reaction. As for HT7, we attempt to decipher SNAP substrate recognition by measuring its affinity for BG-Ac and meAm-TMR. While no affinity could be measured for meAm-TMR, SNAP^C145A^ presented a relatively high affinity for BG-Ac (88.0 μM, 88.6–91.5 μM 95% CI) and CP-Ac (201 μM, 192–212 μM 95% CI) compared to the affinity of HT7 for CA-Ac (Figure S14), which could explain the promiscuity of SNAP.

### Kinetic Characterization of CLIP-tag and SNAP-tag Variants

SNAPf (SNAP^E30R^) is a SNAP variant with faster labeling rates for BG-Alexa488, BG-TMR, BG-Atto549, and BG-AlexaFluor647^60^ (Figure 4A and Figure S15A,B). Fluorescence polarization kinetics of SNAPf revealed a 2–4-fold increase in *k*_app_ compared to that of SNAP for most BG- and CP-fluorophore substrates (Figure 4A, Figure S16, and Table S7). Nevertheless, no increase in labeling kinetics was observed for the best SNAP substrates BG-TMR and BG-CPY (Figure 4A). CLIP^16^ and CLIPf (CLIP^E30R^)^60^ are orthogonal variants of SNAP accepting BC instead of BG substrates (Figure 1B and Figure S15A,C). Labeling kinetics of CLIP and CLIPf (Table S7 and Figure S17) yielded apparent second-order rate constants (*k*_app_) ranging from 10^3^ to 10^5^ M^–1^ s^–1^ with a 2–4-fold increase for CLIPf compared to that for CLIP (Figure 4C). The fastest labeling kinetics were achieved with CLIPf and BC-TMR showing a *k*_app_ of (3.37 ± 0.01) × 10^4^ M^–1^ s^–1^. However, CLIPf is significantly slower than SNAPf (Figure 4D).

### Cross-Reactivity of SNAP- and CLIP-tag Substrates

SNAP and CLIP originate from hAGT^15,16^ (Figure S15A,C), which can potentially react with SNAP and CLIP substrates. We therefore measured the labeling activity of hAGT for the corresponding TMR-based substrates (Figure S18). BG- and CP-TMR labeling of hAGT is 130 and 20 times slower, respectively, than the labeling of SNAP [*k*_app_^BG-TMR^ = (3.38 ± 0.01) × 10^3^ M^–1^ s^–1^; *k*_app_^CP-TMR^ = (3.13 ± 0.01) × 10^3^ M^–1^ s^–1^ (Table 4)]. Interestingly, hAGT shows no preference for BG over CP substrates. BC-TMR reaction with hAGT is 25000 times slower than with CLIP [*k*_app_ = 0.70 ± 0.01 M^–1^ s^–1^ (Table 4)]. Our results suggest that CLIP should be preferred over SNAP in cases in which cross-reactivity of substrates with endogenous hAGT is a concern.

**Table 4:** Labeling Kinetics (*k*_app_) of hAGT, SNAP, and CLIP with TMR Substrates

	*k*_app_ (M^–1^ s^–1^) (value ± standard deviation)		
	hAGT	SNAP	CLIP
BG-TMR	(3.38 ± 0.01) × 10^3^	(4.29 ± 0.01) × 10^5^	(8.26 ± 0.05) × 10^1^
CP-TMR	(3.13 ± 0.01) × 10^3^	(7.69 ± 0.01) × 10^4^	(7.22 ± 0.04) × 10^0^
BC-TMR	(6.25 ± 0.01) × 10^–1^	(3.20 ± 0.02) × 10^2^	(1.85 ± 0.01) × 10^4^

CLIP development was motivated by the perspective to use both SLPs together for multicolor labeling. However, the cross-reactivities of the fastest reacting SNAP and CLIP rhodamine substrates have not yet been determined. Hence, we measured cross-reactivity of BG/CP-TMR with CLIP and BC-TMR with SNAP (Table 4). SNAP reacts more than 1000 times slower with BC-TMR [SNAP *k*_app_^BC-TMR^ = (3.20 ± 0.02) × 10^2^ M^–1^ s^–1^] than with BG-TMR despite the noticeable affinity of SNAP^C145A^ for BC-Ac (416 μM, 408–421 μM 95% CI), which is only 5 times lower than for BG-Ac (Figure S14). CLIP reacts 100 times slower with BG-TMR [CLIP *k*_app_^BG-TMR^ = (8.26 ± 0.05) × 10 M^–1^ s^–1^] than with BC-TMR. These data are in agreement with values previously reported for fluorescein substrates.^16^ Because both proteins show residual reactivity toward their nonrespective substrates, simultaneous co-labeling of both proteins or prior SNAP labeling is advisible to minimize cross-reactions.

### SNAP-tag Is a Promiscuous SLP

Labeling kinetics of nonfluorescent SNAP substrates were characterized by competition kinetics against BG-Alexa488 (Figure S19). Nonfluorescent BG substrates (10^4^ M^–1^ s^–1^ < *k*_app_ < 10^5^ M^–1^ s^–1^) were preferred over CP substrates (10^3^ M^–1^ s^–1^ < *k*_app_ < 10^4^ M^–1^ s^–1^) (Figure 4E and Table 3). In general, SNAP kinetics with nonfluorescent substrates were slower than with fluorescent substrates with the exception of the negatively charged Alexa488. However, in comparison to HT7, the labeling rates of SNAP show a much weaker dependence on the nature of the label (Figure 4E and Table 3).

### Structural Analysis of TMR-Bound SNAP-tag

**Figure 5:**
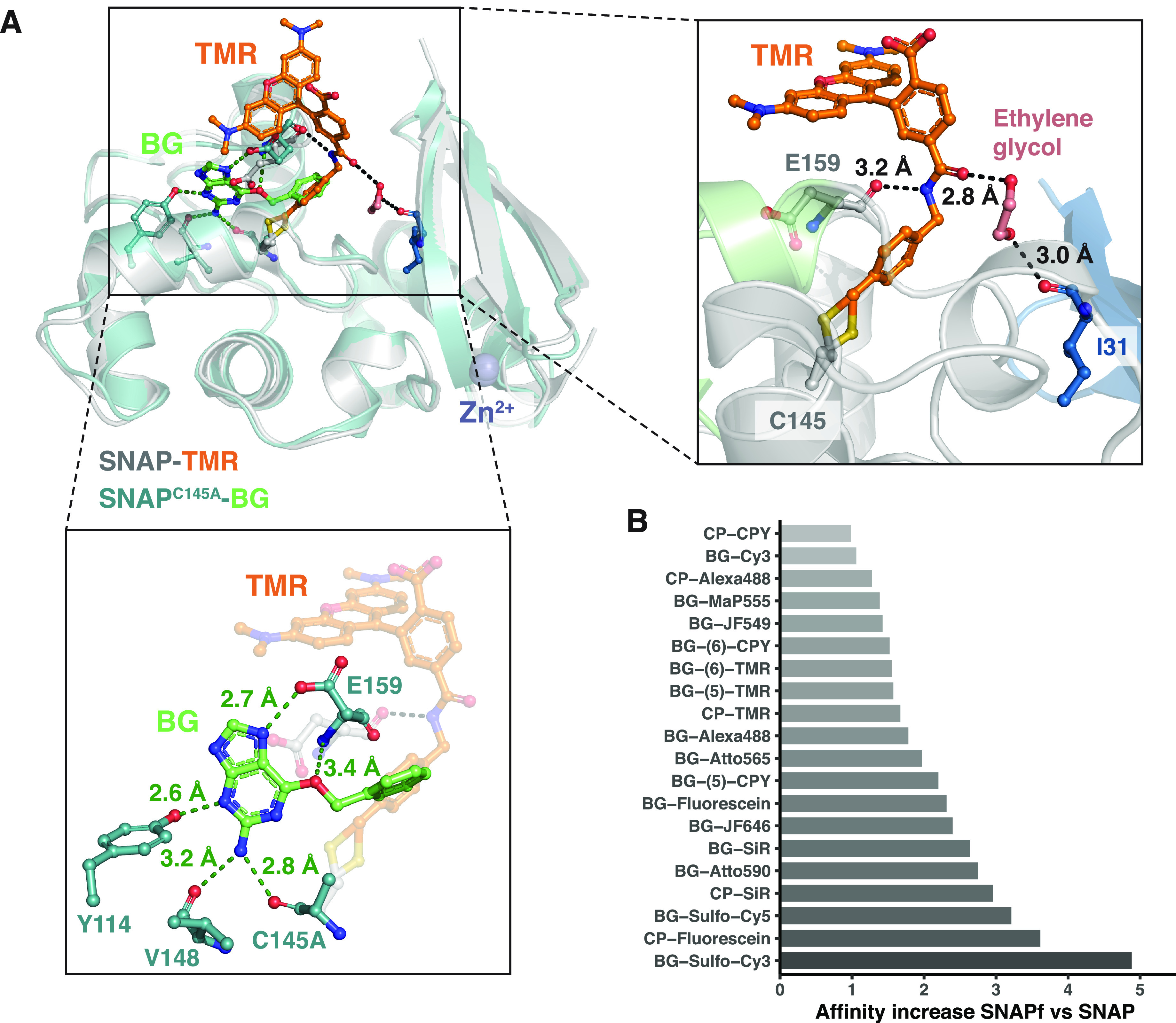
Structure–function analysis of SNAP-tag fluorophore–substrate interactions. (A) Structural comparison between SNAP-TMR (PDB entry 6Y8P) and the BG-bound variant of SNAP^C145A^ (PDB entry 3KZZ). SNAP is represented as a cartoon, and the ligands and residues are represented as sticks. Putative hydrogen bonds and corresponding distances are indicated by dashed lines. (B) Increase in affinity between SNAP^C145A^ and SNAPf^C145A^ for different fluorophore substrates. The number in parentheses indicate different linkages of the fluorophore benzyl group to BG.

To better understand the preference of SNAP for TMR substrates, the X-ray structure of SNAP labeled with TMR was determined at 2.3 Å resolution (PDB entry 6Y8P) (Figure 5A and Table S1). The structure shows the same α/β topology with two domains as observed for hAGT and other SNAP structures.^48,61^ The active site is very similar to the benzylated SNAP structure (PDB entry 3L00),^48^ despite the presence of an alternative cysteine conformation. The TMR moiety strongly participates in the crystal packing, engaging in interactions with the neighboring xanthene ring and protein in a sandwichlike topology (Figure S15D). As a consequence, and in contrast to HT7-TMR, SNAP does not interact with the bound fluorophore in the X-ray structure presented here.

We next evaluated the relative preference for 6- versus 5-carboxy isomers of TMR and CPY substrates by studying their labeling rates (Figure S20 and Table S8) and affinities (Figure S13) for SNAP, SNAPf, and their inactive variants. SNAP and SNAPf showed 10-fold slower reaction rates with 5-fluorophores (*k*_app_ ≈ 10^4^–10^5^ M^–1^ s^–1^) than with the corresponding 6-fluorophores (*k*_app_ ≥ 10^5^ M^–1^ s^–1^). These differences were even more pronounced for the affinities, which were up to 25-fold higher for the 6-carboxy isomers.

In the crystal structure of TMR-labeled SNAP, a structural ethylene glycol forms hydrogen bonds with both the backbone carbonyl oxygen of I31 and the carbonyl oxygen of the amide linking the benzyl to the fluorophore (Figure 5A). This benzyl-fluorophore amide is also forming a hydrogen bond to the backbone carbonyl oxygen of residue E159 via its Nα atom. Comparison with the BG-bound SNAP^C145A^ structure (PDB entry 3KZZ) (Figure 5A) suggests that, after reaction, the E159 side chain flips inside the BG binding cavity, resulting in a reorientation of its backbone carbonyl oxygen that can then interact with the amide of the substrate (Figure 5A).

### SNAPf Has a Higher Affinity for Its Substrates

We modeled the SNAPf mutation E30R in the structure of TMR-labeled SNAP to gain a better understanding of how it affects the labeling kinetics (Figure S15B). The results suggest that an arginine at position 30 could interact with the carbonyl oxygen of the amide group in the label via a moderate hydrogen bond (3.2 Å), replacing the hydrogen bond observed with the ethylene glycol in the crystal structure. This could lead to an increased affinity for the substrate or a better substrate positioning for labeling. To probe this hypothesis, the affinities of SNAP^C145A^ and SNAPf^C145A^ were compared side by side for various fluorophore substrates (Figure S13). Among the 20 fluorophore substrates tested, only five did not show a significant increase in affinity (i.e., >50%) and nine showed a >2-fold increase in affinity (Figure 5B). As observed for SNAP, SNAPf^C145A^ substrate affinities correlate well with the corresponding *k*_app_ values for SNAPf (Figure S21). It is worth mentioning that negatively charged substrates such as BG-sulfo-Cy3 show the largest increase in protein affinities and labeling rates upon comparison of SNAP to SNAPf. This could be due to the exchange of the negatively charged glutamic acid with a positively charged arginine, resulting in a potential electrostatic steering effect as mentioned for HT7.^59^

### Comparison between SNAP-tag and SsOGT-H^5^

Recently, a homologue of hAGT from an extremophile archaeon was converted to an SLP (*Ss*OGT-H^5^) by introducing mutations that have been shown to increase the reactivity of SNAP.^62^ Its crystal structure labeled with SNAP-Vista Green (SVG, i.e., BG-5-fluorescein)^63^ shows a different fluorophore conformation (Figure S15E), constrained by the crystal packing. Interestingly, the *Ss*OGT-H^5^-SVG structure was obtained with a fluorophore connected via the 5-carboxy isomer of the fluorophore and presents a substrate conformation that could not exist in the SNAP structure due to steric clashes. We compared the kinetics of SNAP and *Ss*OGT-H^5^ (Figure S22 and Table S9) toward the substrates BG-TMR (5- and 6-substituted) and BG-6-Alexa488 at 37 °C. In contrast to SNAP, *Ss*OGT-H^5^ showed a preference for BG-5-TMR [*k*_app_ = (1.45 ± 0.92) × 10^2^ M^–1^ s^–1^] over BG-6-TMR [*k*_app_ = (6.78 ± 0.67) × 10 M^–1^ s^–1^]. Furthermore, the negatively charged BG-6-Alexa488 [*k*_app_ = (1.24 ± 0.01) × 10^2^ M^–1^ s^–1^] presents kinetics in the same range as those of BG-5-TMR, highlighting a different substrate preference between SNAP and *Ss*OGT-H^5^. For all substrates, *Ss*OGT-H^5^ presents kinetics 100 times slower than those of SNAP or CLIP, making it less suitable for labeling applications at physiological temperatures.

## Conclusion

We provide here a systematic comparison of the labeling kinetics of HT7, SNAP, and CLIP for a large panel of substrates. A structure–function relationship analysis complements this comparison, thereby yielding insights into the origins of the different substrate specificities of HT7 and SNAP. The data should assist scientists in choosing SLP–substrate pairs for specific purposes.

**Figure 6:**
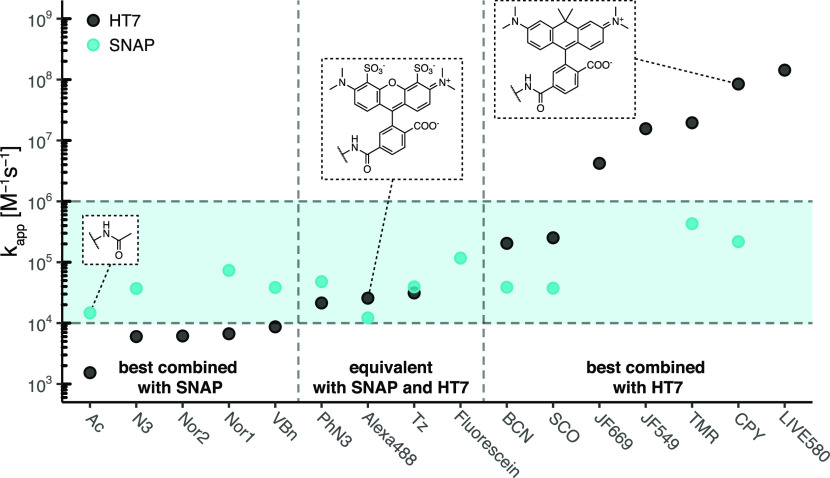
Comparison of labeling kinetics between SNAP-tag and HaloTag7. Apparent labeling rate constants (*k*_app_) of HT7 span >6 orders of magnitude, while rate constants of SNAP span only >2 orders of magnitude (BG-substrates). The blue area highlights the span of SNAP apparent labeling rate constants. Depending on the application, some substrates should preferentially be employed with HT7 or SNAP to ensure quick labeling. A rate constant of 10^5^ M^–1^ s^–1^ corresponds to a half-labeling time of ∼7 s at 1 μM substrate, in excess.

The direct comparison of SNAP and HT7 reveals that HT7 features significantly higher labeling rate constants with various fluorescent rhodamine derivatives (Figure 6 and Table 3). These differences in reactivity can be explained by specific interactions of the rhodamine’s xanthene ring with selected surface residues of HT7. The high reactivity of HT7 toward rhodamines is important as rhodamines to date represent the most relevant class of cell-permeable fluorophores for live-cell imaging. The interactions between rhodamines and HT7 also help to explain why some rhodamine-based HT7 substrates tend to have improved spectroscopic properties and are more fluorogenic than the corresponding SNAP or CLIP substrates.^21^ Most rhodamine-based fluorophores exist in an equilibrium between spirocyclic nonfluorescent and zwitterionic fluorescent forms. While in solution the spirocyclic form might be favored, the labeling reaction with an SLP switches this equilibrium toward the zwitterionic form, leading to an increase in fluorescence intensity.^64^ This property is of particular interest in wash-free live-cell fluorescence microscopy because it leads to a higher signal over background^28−30,60,65^ and can also be exploited for sensor design.^57,66^ Furthermore, the dynamic equilibrium between the spirocyclic nonfluorescent and zwitterionic fluorescent form is crucial for cell permeability.^29^ The mechanism underlying the equilibrium shift from the spirocyclic nonfluorescent to the zwitterionic fluorescent form is not yet fully understood, but our results indicate that the planar, zwitterionic form of rhodamines (e.g., TMR and CPY) features energetically favorable interactions with the HT7 surface, thus potentially favoring this state of the fluorophore when attached to the protein.

While HT7 reacts quicker with most rhodamine-based fluorophore substrates than SNAP, the differences become much less pronounced or reversed for negatively charged substrates. For example, SNAP reacts faster with Alexa488 than HT7 and the reactivity for most other nonfluorescent substrates tends to be higher for SNAP, as well (Figure 6 and Table 3). It is interesting to hypothesize about the origin of the differences in substrate specificity between SNAP and HT7. Most likely, these differences are, at least partially, a consequence of the substrates used in the engineering of the tags. For HT7, TMR was used in most screening assays,^14,18^ and as a result, HT7 shows a specificity for zwitterionic rhodamines. In contrast, different substrates such as BG-fluorescein,^67^ BG-Cy3^68^ as well as affinity reagents such as BG-biotin^67^ were used in SNAP screening and selection assays. As a consequence, SNAP is more promiscuous than HT7. Differences in the speed of labeling of both SLPs are mostly driven by differences in substrate affinity: an overall correlation between affinity and rate constants was observed for both proteins that was more pronounced for HT7. Indeed, HT7 presents a very low affinity for the, e.g., unsubstituted CA-Ac substrate, highlighting that the affinity of HT7 for substrates is strongly driven by the substituent and so are the kinetics. We show here how the low reactivity of HT7, for example, toward CA-PEG-biotin, can be overcome by designing substrates in which the label of interest is attached to a CA-TMR core and anticipate that such a strategy could be expanded to other substituents. In addition, it might be possible to replace the rhodamine scaffold with smaller structures that could be used to increase the affinity of poor HT7 substrates while maintaining good cell permeability.

Indeed, a key property of SLP substrates for live-cell applications that we have not addressed in this study is their cell permeability. In general, the CA core is less polar and possesses a molecular weight slightly lower than those of BG, CP, and BC. The permeability of HT7 substrates therefore can be expected to be higher than those of the corresponding SNAP-tag substrates. Furthermore, the SLP labeling rates measured *in vitro* might not translate precisely to labeling rates in the crowded environment of a cell due to slower diffusion rates of small molecules in cells and the potentially decreased concentration of the free substrate as a result of weak, unspecific binding to other macromolecules.^69^ Our results remain valuable for choosing SLP substrate pairs because the overall trends of labeling rates should translate to *in cellulo* environments. However, the question of substrate permeability and attenuated diffusion will have to be addressed more systematically in future studies. High affinity and fast labeling kinetics (*k*_app_ > 10^6^ M^–1^ s^–1^) are highly desirable features of SLP–substrate pairs because they allow efficient labeling at low concentrations of fluorescent substrates. This is particularly important for applications in which the amount of available substrate is limited, e.g., in the case of a poorly permeable substrate or for *in vivo* SLP labeling. Moreover, using low nanomolar concentrations of fluorophore substrates for no-wash live-cell imaging decreases the intensity of the unspecific signal and effectively increases signal:background ratios, leading to improved image quality.

For future engineering of SLPs, it would be particularly interesting to increase the affinity of SNAP and CLIP for rhodamine-based substrates. Given the importance of these fluorophores for live-cell fluorescence (super-resolution) microscopy,^6^ additional tags that display labeling kinetics toward rhodamines similar to those of HT7 would be strongly welcomed. Our results suggest that increasing the reactivity toward these dyes might come with the risk of reducing the activity toward other substrates, thereby limiting the flexibility of such tags. However, given the importance of SLPs and rhodamine-based probes for live-cell imaging, the generation of such specialized tags is warranted.

## Supporting Information Available

Chemical synthesis, material suppliers and references, chemical structures, raw biochemical–kinetic data and fits, supplementary kinetic analysis, X-ray collection and refinement statistics, supplementary structural analysis, protein sequences, and examples of Dynafit scripts (PDF)

## Accession Codes

DhaA, Uniprot entry P59336; hAGT, Uniprot entry P16455. Atomic coordinates and structure factors were deposited as PDB entries 6Y7A (HT7-TMR), 6ZCC (HOB-TMR), 6Y7B (HT7-CPY), and 6Y8P (SNAP-TMR).
