# Supplementary material for: Kinetic and Structural Characterization of the Self-Labeling Protein Tags HaloTag7, SNAP-tag, and CLIP-tag
Source: Biochemistry. 2021 Aug 2;60(33):2560–75. doi: 10.1021/acs.biochem.1c00258 (PMC8388125; doi:10.1021/acs.biochem.1c00258)
Supplement: Supplementary file 1 — bi1c00258_si_001.pdf [file bi1c00258_si_001.pdf]

Supporting information

for

Kinetic and structural characterization of the self-labeling protein tags HaloTag7, SNAP-tag and CLIP-tag

Jonas Wilhelm[1,9], Stefanie Kühn[1,9], Miroslaw Tarnawski[2], Guillaume Gotthard[3,7], Jana Tünnermann[1], Timo Tänzer[4], Julie Karpenko[4,8], Nicole Mertes[1], Lin Xue[1], Ulrike Uhrig[5], Jochen Reinstein[6], Julien Hiblot[1,4,10]* and Kai Johnsson[1,4,10]*.

[1]Department of Chemical Biology, Max Planck Institute for Medical Research, 69120, Heidelberg, Germany.

[2]Protein Expression and Characterization Facility, Max Planck Institute for Medical Research, 69120, Heidelberg, Germany.

[3]Structural Biology Group, European Synchrotron Radiation Facility (ESRF), 38043, Grenoble, France.

[4]Institute of Chemical Sciences and Engineering, École Polytechnique Fédérale de Lausanne (EPFL), 1015, Lausanne, Switzerland.

[5]Chemical Biology Core Facility, European Molecular Biology Laboratory, 69117, Heidelberg, Germany.

[6]Department of Biomolecular Mechanisms, Max Planck Institute for Medical Research, 69120, Heidelberg, Germany.

[7]Present addresses: Division of Biology and Chemistry–Laboratory for Biomolecular Research, Paul Scherrer Institute, 5232, Villigen, Switzerland. Department of Biology, Institute of Molecular Biology and Biophysics, ETH Zürich, 8093, Zürich, Switzerland.

[8]Present address: Laboratoire d'Innovation Thérapeutique, UMR7200 CNRS/Université de Strasbourg, Strasbourg Drug Discovery and Development Institute (IMS), 67401 Illkirch-Graffenstaden, France.

[9]These authors contributed equally: Jonas Wilhelm, Stefanie Kühn.

[10]These authors contributed equally: Julien Hiblot, Kai Johnsson.

* e-mail: julien.hiblot@mr.mpg.de; johnsson@mr.mpg.de

For Table of Contents use only

	Page
**Chemistry**	S4-27
General information	S4
**Material Table:** Sources of substrates and chemicals used in this study	S5-7
Chemical Synthesis	S8-27
**Supplementary figures**	S28-49
**Figure S1:** Chemical structure of SLP substrates	S28-29
**Figure S2:** Labeling kinetics of HT7 with fluorescent CA substrates	S30
**Figure S3:** Comparison of model 1 and model 2 fitted to HT7 labeling kinetics	S31
**Figure S4:** Modeling of HT7 labeling kinetics using measured parameters to compare the kinetic models 1 and 2	S32
**Figure S5:** Labeling kinetics of HT7 and HOB with CA-TMR and CA-Alexa488	S33
**Figure S6:** Rate and equilibrium constants of HT7 labeling with various fluorescent CA substrates	S34
**Figure S7:** Affinity of the dead mutant $HT7^{D106A}$ to fluorescent CA substrates	S35
**Figure S8:** Labeling kinetics of HT7 with non-fluorescent CA substrates	S36
**Figure S9:** Additional structural information on HaloTag proteins	S37
**Figure S10:** Biochemical study of the interaction of HT7 with chloroalkane-fluorophores	S38
**Figure S11:** Labeling kinetics of SNAP with fluorescent BG and CP substrates	S39
**Figure S12:** Labeling kinetics of SNAP measured by stopped flow anisotropy	S40
**Figure S13:** Comparison of fluorophore substrate affinities between the dead mutants $SNAP^{C145A}$ and $SNAPf^{C145A}$	S41
**Figure S14:** Comparison of non-derivatized core substrate affinities to the dead mutant $SNAP^{C145}$	S42
**Figure S15:** Sequence and additional structural information related to SNAP.	S43
**Figure S16:** Labeling kinetics of SNAPf with fluorescent BG and CP substrates	S44
**Figure S17:** Labeling kinetics of CLIP and CLIPf with fluorescent BC	S45
**Figure S18:** Labeling kinetics of hAGT, SNAP and CLIP with the non-respective BG-, CP- and BC-TMR substrates	S46
**Figure S19:** Labeling kinetics of SNAP with non-fluorescent BG and CP substrates	S47
**Figure S20:** Labeling kinetics of SNAP and SNAPf with BG-5-TMR and BG-5-CPY	S48
**Figure S21:** Correlation between SNAPf labeling kinetics and substrate affinity	S48
**Figure S22:** Labeling kinetics of *Ss*OGT-H^5 with BG-Alexa488 and BG-TMR	S49
**Supplementary tables**	S50-52
**Table S1:** Data collection and refinement statistics the X-ray crystal structures	S50
**Table S2:** Kinetic parameters of HT7 labeling with fluorescent CA substrates	S51
**Table S3:** Comparison k_{app} of HT7 labeling kinetics analyzed using models 1 and 2	S51
**Table S4:** Comparison of HT7 and HOB labeling kinetics with fluorescent CA substrates	S51
**Table S5:** Kinetic parameters of SNAP and SNAPf labeling with fluorescent substrates analyzed using model 1.2	S52
**Table S6:** Kinetic parameters of SNAP labeling with BG-/CP-TMR measured via stopped flow	S52
**Table S7:** Comparison of SNAP/CLIP with SNAPf/CLIPf labeling kinetics with fluorescent substrates	S52
**Table S8:** Comparison of SNAP labeling with 5- and 6-fluorophores.	S52
**Table S9:** Kinetic parameters of *Ss*OGT-H^5 labeling	S52
**Protein sequences**	S53-54

Examples DynaFit scripts S55-59

References S60-61

Chemical Synthesis

General information

All chemical reagents and (anhydrous) solvents for synthesis were purchased from commercial suppliers (Merck KGaA, Darmstadt, Germany; Honeywell, Charlotte, NC, USA; TCI, Tokyo, Japan; Thermo Fisher Scientific, Waltham, MA, USA; SiChem, Bremen, Germany) and were used without further purification or distillation. Anhydrous solvents were handled under argon atmosphere. SLP substrates were purchased from commercial sources, synthesized according to published procedures or gifts from colleagues. Details are given in **Material Table**.

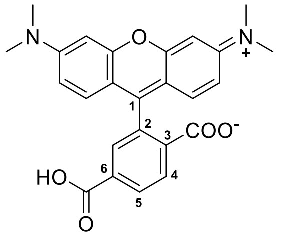

^{1}H- and ^{13}C-NMR spectra were recorded in deuterated solvents on a Bruker (Bruker Corp., Billerica, MA, USA) DPX400 (400 MHz for ^{1}H, 101 MHz for ^{13}C, respectively) or on a Bruker AVANCE III HD 400 (400 MHz for ^{1}H, 101 MHz for ^{13}C, respectively) equipped with a CryoProbe. Chemical shifts (δ) are reported in ppm referenced to the residual solvent peaks of DMSO-*d*$_6$ (δ_H = 2.50 ppm, δ_C = 39.52 ppm), acetone-*d*$_6$ (δ_H = 2.05 ppm, $\delta_C(CH_3)$ = 29.84 ppm, $\delta_C(CO)$ = 206.26 ppm) or $CDCl_3$ (δ_H = 7.26 ppm, δ_C = 77.16 ppm). Coupling constants *J* are reported in Hz and corresponding multiplicities are abbreviates as follows: s = singlet, d = doublet, t = triplet, q = quartet, p = pentet, m = multiplet and br = broad.

Reaction progress was monitored by thin layer chromatography (TLC) (Silica gel 60G F_{254} on TLC glass plates) in appropriate solvents. Reaction spots were visualized under UV lamp (254 nm or 366 nm) and/or by staining solutions. LC-MS was performed on a Shimadzu MS2020 (Shimadzu Corp., Kyoto, Japan) connected to a Nexera UHPLC system equipped with a Waters (Waters Crop., Milford, MA, USA) ACQUITY UPLC BEH C18 (1.7 µm, 2.1x50 mm) column. Buffer A: 0.1% formic acid in H_2O, Buffer B: acetonitrile. Measurements were done with an analytical gradient from 10% to 90% B over 6 min or from 1% to 90% B over 10 min.

Normal phase flash chromatography was performed on self-packed silica gel (60 M, 0.04 - 0.063 mm, Macherey-Nagel GmbH & Co. KG, Düren, Germany) columns or by using an Isolera One system (Biotage Sweden AB, Uppsala, Sweden) using pre-packed silica gel columns (ultra pure silica gel 12 g or 25 g). Solvent compositions are reported individually in parentheses.

Preparative reversed phase high-performance liquid chromatography (RP-HPLC) was conducted using a Waters SunFire™ Prep C18 OBDTM column (10 × 150 mm, 5 µm pore size, 4 mL/min. flow rate) or an Ascentis (Merck KGaA, Darmstadt, Germany) C18 column (10 × 250 mm, 5 µm pore size, 8 mL/min. flow rate) on either a Waters Alliance e2695 separation module connected to a 2998 PDA detector or a Dionex system equipped with an UVD (170 U, UV-Vis detector). Solvent A: 0.1%TFA in H_2O, Solvent B: acetonitrile.

High resolution mass spectra (HRMS) were measured by the MS-service of the EPF Lausanne (SSMI) on a Waters Xevo® G2-S Q-Tof spectrometer (Waters, Milford, MA, USA) with electron spray ionization (ESI) or by the MS-facility of the Max Planck Institute for Medical Research on a Bruker maXis IITM ETD.

Material Table: Substrate and chemical source used in the study

	Substrate	Source / reference
General	CPY-6-COOH	Butkevich *et al.,* (2016) [1]
	CPY-5-COOH	Butkevich *et al.,* (2016) [1]
	TMR-5-COOH	Mudd *et al.,* (2015) [2]
	TMR-6-COOH	Mudd *et al.,* (2015) [2]
	Cy3-COOH	Ueno *et al.,* (2011) [3]
	Cy5-COOH	Ueno *et al.,* (2011) [3]
	SiR-COOH	Lukinavicius *et al.,* (2013) [4]
	meAm-6-TMR	this study
	meAm-5-TMR	this study
	meAm-6-CPY	this study
	meAm-5-CPY	this study
HaloTag substrates	CA-TMR	Purchased from Promega, Madison, WI, USA
	CA-Alexa488	Purchased from Promega, Madison, WI, USA
	CA-Fluorescein	Purchased from Promega, Madison, WI, USA
	CA-Oregon green	Purchased from Promega, Madison, WI, USA
	CA-JF549	Gift from Dr. Luke Lavis, HHMI, Ashburn, VA, USA
	CA-JF503	Gift from Dr. Luke Lavis, HHMI, Ashburn, VA, USA
	CA-JF525	Gift from Dr. Luke Lavis, HHMI, Ashburn, VA, USA
	CA-JF608	Gift from Dr. Luke Lavis, HHMI, Ashburn, VA, USA
	CA-JF669	Gift from Dr. Luke Lavis, HHMI, Ashburn, VA, USA
	CA-TMR-az-F_4	Gift from Dr. Luke Lavis, HHMI, Ashburn, VA, USA
	CA-TMR-CN	Wang *et al.,* (2020) [5]
	CA-TMR-SCH_3	Wang *et al.,* (2020) [5]
	CA-TMR-SNH_2	Wang *et al.,* (2020) [5]
	CA-MaP555	Wang *et al.,* (2020) [5]
	CA-CPY	Butkevich *et al.,* (2016) [1]
	CA-500R	Butkevich *et al.,* (2016) [1]
	CA-510R	Purchased from Abberior GmbH, Göttingen, Germany
	CA-515R	Purchased from Abberior GmbH, Göttingen, Germany
	CA-580CP	Gift from Dr. Alexey N. Butkevich, MPI-MF, Heidelberg, Germany
	CA-LIVE580	Purchased from Abberior GmbH, Göttingen, Germany
	CA-Cy3	this study
	CA-Cy5	this study
	CA-TMR-biotin	this study
	CA-PEG-biotin	Purchased from Promega, Madison, WI, USA
	CA-Ac	this study
	CA-N_3	this study
	CA-Nor1	this study
	CA-Nor2	this study
	CA-Tz	this study

	CA-PhN_3	this study
	CA-Vbn	this study
	CA-BCN	this study
	CA-SCO	this study
**SNAP substrates**	BG	Purchased from Santa Cruz Biotechnology, Dallas, TX, USA
	CP	this study
	BG-NH_2	Keppler *et al.,* (2003) [6]
	CP-NH_2	Srikun *et al.*, (2010) [7]
	BG-TMR	Keppler *et al.,* (2004) [8]
	CP-TMR	Correa *et al.*, (2013) [9]
	BG-Alexa488	Purchased from NEB as SNAP-Surface® Alexa Fluor® 488, Ipswitch, MA, USA
	CP-Alexa488	this study
	BG-Fluorescein	Keppler *et al.,* (2003) [6]
	CP-Fluorescein	this study
	BG-CPY	Hiblot *et al.,* (2017) [10]
	CP-CPY	this study
	BG-5-TMR	this study
	BG-5-CPY	this study
	BG-MaP555	Wang *et al.,* (2020) [5]
	BG-SiR	Lukinavicius *et al.,* (2013) [4]
	CP-SiR	this study
	BG-JF549	Grimm *et al.,* (2015) [11]
	BG-JF646	Grimm *et al.,* (2015) [11]
	BG-Cy3	this study
	BG-sulfo-Cy3	Gautier *et al.,* (2008) [12]
	BG-Cy5	this study
	BG-sulfo-Cy5	Gautier *et al.,* (2008) [12]
	BG-Atto565	Correa *et al.,* (2013) [9]
	BG-Atto590	Bottanelli *et al.,* (2016) [13]
	BG-N_3	this study
	CP-N_3	this study
	BG-Nor2	this study
	CP-Nor2	this study
	BG-Tz	this study
	CP-Tz	this study
	BG-PhN_3	this study
	CP-PhN_3	this study
	BG-Vbn	this study
	CP-Vbn	this study
	BG-BCN	this study
	CP-BCN	this study
	BG-Ac	this study
	CP-Ac	this study

	BG-SCO	this study
	CP-SCO	this study
**CLIP substrates**	$BC-NH_2$	Gautier *et al.,* (2008) [12]
	BC-TMR	Gautier *et al.,* (2008) [12]
	BC-Alexa488	Purchased from NEB as CLIP-Surface® Alexa Fluor® 488, Ipswitch, MA, USA
	BC-Fluorescein	Gautier *et al.,* (2008) [12]
	BC-CPY	this study

Chemical Synthesis

1.1 Synthesis of substrate amines

1.1.1 2-(2-((6-chlorohexyl)oxy)ethoxy)ethan-1-amine (CA-NH_2)

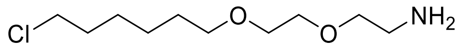

CA-NH_2 was synthesized according to the procedure from Zhang *et al*. 2006 [14].

1.1.2 6-((4-(aminomethyl)benzyl)oxy)-9*H*-purin-2-amine (BG-NH_2)

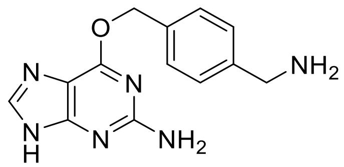

BG-NH_2 was synthesized according to the procedure from Keppler *et al*. 2003 [6].

1.1.3 4-((4-(aminomethyl)benzyl)oxy)-6-chloropyrimidin-2-amine (CP-NH_2)

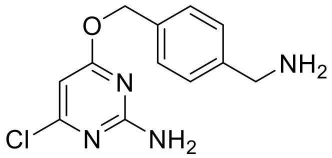

CP-NH_2 was synthesized according to the procedure from Srikun *et al*. 2010 [7].

1.1.4 2-((4-(aminomethyl)benzyl)oxy)pyrimidin-4-amine (BC-NH_2)

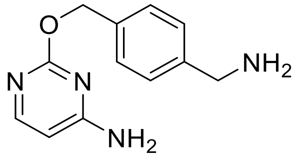

BC-NH_2 was synthesized according to the procedure from Gautier *et al*. 2008 [12].

1.2 General procedure A for peptide coupling reactions

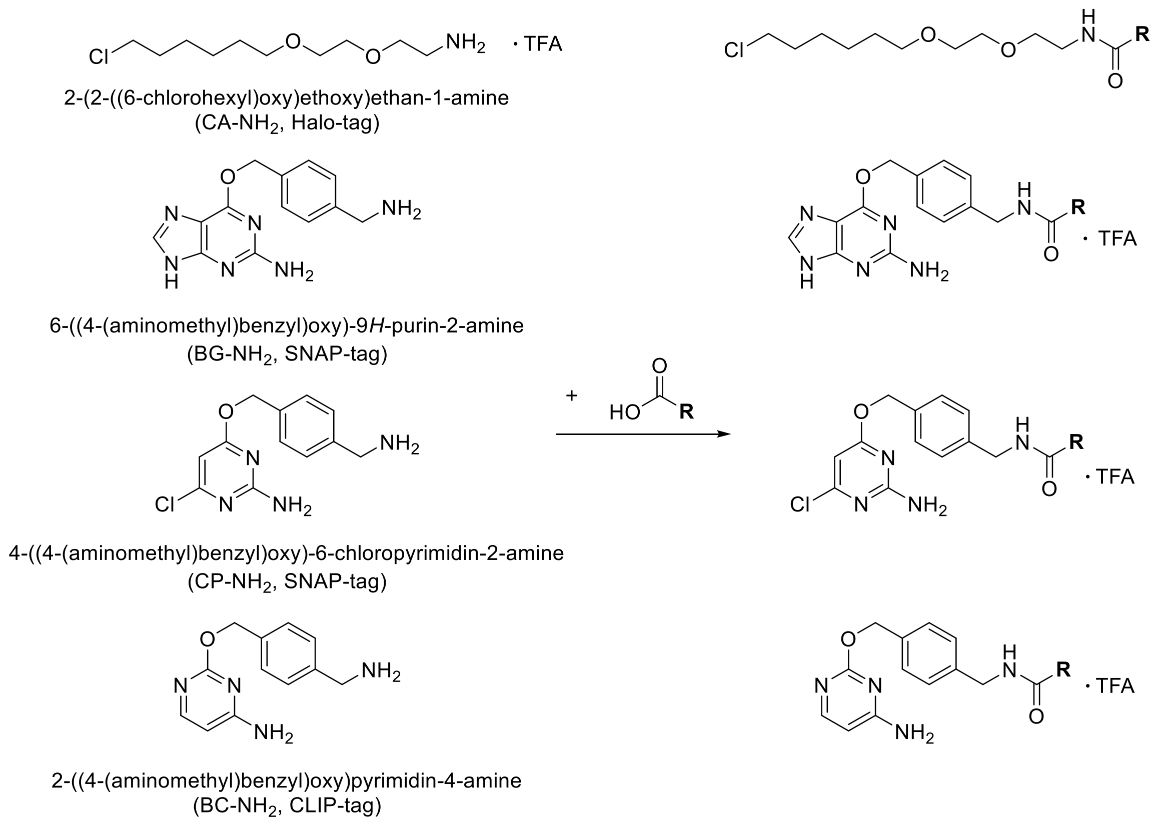

To a solution of TSTU (1.2 equiv.) in dry DMSO (0.3 mL), DIPEA (10.2 equiv. for Halo-tag-, 5.0 equiv. for SNAP-substrates) and different carboxylic acids (1.1 equiv.) were added. After 5 min., a solution of 10 mg of corresponding amine (1.0 equiv.) in dry DMSO (0.1 mL) was added and the reaction mixture was stirred at r.t. for 2 hours. The reaction mixture was quenched by addition of water (100 µL) and acidified with acetic acid (50 µL), then purified by semi-preparative HPLC, eluted with a gradient of $MeCN/H_2O$ + 0.1% TFA (equilibration at 15% MeCN for 5 min, then gradient of 15 - 100% MeCN over 25 min, followed by 100% MeCN for 10 min.). Fractions containing the desired product were combined and lyophilized. Final compounds were stored as DMSO stocks for biochemical testing.

1.3 HT7 substrates

1.3.1 2-azido-N-(2-(2-((6-chlorohexyl)oxy)ethoxy)ethyl)acetamide (CA-N_3)

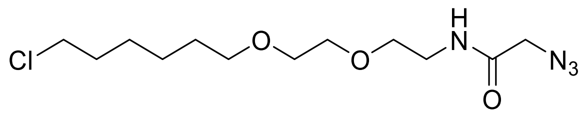

Reaction was conducted according to general procedure A using CA-NH_2 and 2-azidoacetic acid (4.6 µL, 32.6 µmol). The desired product (4.6 mg, 15.0 µmol) was obtained as a yellowish oil in 51% yield.

^{1}H NMR (400 MHz, DMSO-*d*$_6$) *δ* [ppm] = 8.15 (t, *J* = 5.8 Hz, 1H), 3.81 (s, 2H), 3.62 (t, *J* = 6.6 Hz, 2H), 3.53 – 3.40 (m, 6H), 3.37 (t, *J* = 6.6 Hz, 2H), 3.24 (dd, *J* = 5.7 Hz, *J* = 5.8 Hz, 2H), 1.75 – 1.65 (m, 2H), 1.54 – 1.43 (m, 2H), 1.43 – 1.25 (m, 4H).

^{13}C NMR (101 MHz, DMSO-*d*$_6$) *δ* [ppm] = 167.31, 70.17, 69.56, 69.40, 68.83, 50.69, 45.36, 38.67, 32.00, 29.04, 26.10, 24.91.

HRMS (ESI): calc. for $C_{12}H_{23}ClN_4NaO_3^+$ $[M+Na]^+$: 329.1351; found 329.1354.

1.3.2 (1R,4R)-N-(2-(2-((6-chlorohexyl)oxy)ethoxy)ethyl)bicyclo[2.2.1]hept-5-ene-2-carboxamide (CA-Nor1)

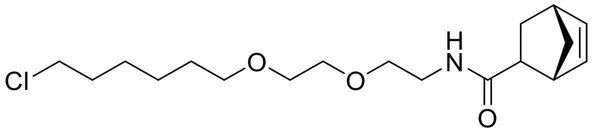

Reaction was conducted according to general procedure A using CA-NH_2 and (1R,4R)-bicyclo[2.2.1]hept-5-ene-2-carboxylic acid (6.2 µL, 32.6 µmol). The desired endo-isomer (5.6 mg, 16.3 µmol) of was obtained in 55% yield.

^{1}H NMR (400 MHz, DMSO-d_6) δ [ppm] = 7.59 (t, J = 5.7 Hz, 1H), 6.08 (dd, J = 5.8, 3.1 Hz, 1H), 5.80 (dd, J = 5.8, 3.0 Hz, 1H), 3.62 (t, J = 6.6 Hz, 2H), 3.54 – 3.30 (m, 8H), 3.23 – 3.02 (m, 3H), 2.84 – 2.71 (m, 2H), 1.77 – 1.63 (m, 3H), 1.55 – 1.43 (m, 2H), 1.42 – 1.18 (m, 7H).

^{13}C NMR (101 MHz, DMSO-d_6) δ [ppm] = 172.86, 136.76, 132.18, 70.18, 69.58, 69.45, 69.09, 49.35, 45.59, 45.37, 43.25, 42.08, 38.55, 32.02, 29.09, 28.35, 26.13, 24.94.

HRMS (ESI) calc. for $C_{18}H_{31}ClNO_3^+$ [M+H]$^+$: 344.1987; found 344.1989.

1.3.3 2-((1S,4S)-bicyclo[2.2.1]hept-5-en-2-yl)-N-(2-(2-((6-chlorohexyl)oxy)ethoxy)ethyl)acetamide (CA-Nor2)

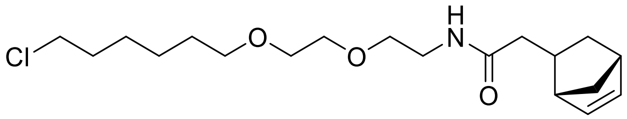

Reaction was conducted according to general procedure A using CA-NH_2 and 2-((1S,4S)-bicyclo[2.2.1]hept-5-en-2-yl)acetic acid (5.6 µL, 32.6 µmol) yielding 6.4 mg (17.9 µmol) of the desired product as a colorless oil in 60% yield.

^{1}H NMR (400 MHz, DMSO-d_6) δ [ppm] = 7.73 (t, J = 5.7 Hz, 1H), 6.15 (dd, J = 5.8, 3.0 Hz, 1H), 5.95 (dd, J = 5.8, 2.9 Hz, 1H), 3.62 (t, J = 6.6 Hz, 2H), 3.47 – 3.36 (m, 8H), 3.23 – 3.09 (m, 2H), 2.76 – 2.68 (m, 2H), 2.40 – 2.29 (m, 1H), 1.89 – 1.74 (m, 3H), 1.74 – 1.65 (m, 2H), 1.53 – 1.43 (m, 2H), 1.39 – 1.17 (m, 6H), 0.47 (m, J = 11.5, 4.4, 2.6 Hz, 1H).

^{13}C NMR (101 MHz, DMSO-d_6) δ [ppm] = 171.61, 136.88, 132.47, 70.15, 69.52, 69.42, 69.08, 49.03, 45.32, 45.13, 42.02, 40.58, 40.14, 39.93, 39.73, 39.51, 39.31, 39.10, 38.89, 38.35, 35.06, 31.99, 31.37, 29.04, 26.08, 24.89.

HRMS (ESI) calc. for $C_{19}H_{32}ClNNaO_3^+$ [M+Na]$^+$: 380.1963; found 380.1963.

1.3.4 N-(2-(2-((6-chlorohexyl)oxy)ethoxy)ethyl)-2-(4-(6-methyl-1,2,4,5-tetrazin-3-yl)phenyl)acetamide (CA-Tz)

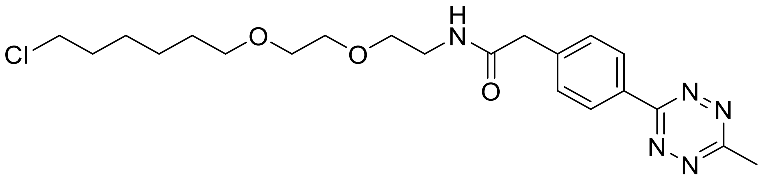

Reaction was conducted according to general procedure A using CA-NH_2 and 2-(4-(6-methyl-1,2,4,5-tetrazin-3-yl)phenyl)acetic acid (7.5 mg, 32.6 µmol) yielding 7.4 mg (17.0 µmol) of the desired product as a rose solid in 57% yield.

^{1}H NMR (400 MHz, DMSO-d_6) δ [ppm] = 8.44 – 8.36 (m, 2H), 8.23 (t, J = 5.6 Hz, 1H), 7.58 – 7.50 (m, 2H), 3.61 (t, J = 6.6 Hz, 2H), 3.56 (s, 2H), 3.53 – 3.40 (m, 6H), 3.36 (t, J = 6.6 Hz, 2H), 3.23 (q, J = 5.7 Hz, 2H), 2.99 (s, 3H), 1.75 – 1.62 (m, 2H), 1.53 – 1.42 (m, 2H), 1.42 – 1.25 (m, 4H).

^{13}C NMR (101 MHz, DMSO-d_6) δ [ppm] = 169.58, 167.05, 163.22, 141.29, 130.05, 130.00, 127.28, 70.20, 69.60, 69.45, 69.05, 45.37, 42.19, 38.79, 32.02, 29.07, 26.12, 24.93, 20.83.

HRMS (ESI) calc. for $C_{21}H_{31}ClN_5O_3^+$ [M+H]$^+$: 436.2110; found 436.2113.

1.3.5 4-azido-N-(2-(2-((6-chlorohexyl)oxy)ethoxy)ethyl)benzamide (CA-PhN$_3$)

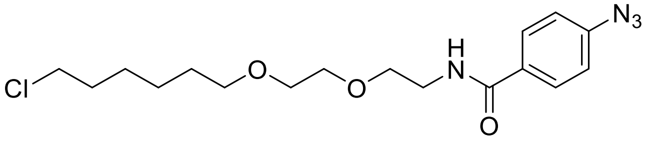

Reaction was conducted according to general procedure A using CA-NH$_2$ and 4-azidobenzoic acid (5.3 mg, 32.6 µmol) to obtain 6.1 mg (15.5 µmol) of the desired product as a colorless oil in 56% yield.

^{1}H NMR (400 MHz, DMSO-d_6) δ [ppm] = 8.52 (t, J = 5.6 Hz, 1H), 7.90 (d, J = 8.6 Hz, 2H), 7.20 (d, J = 8.6 Hz, 2H), 3.60 (t, J = 6.6 Hz, 2H), 3.56 – 3.49 (m, 4H), 3.50 – 3.44 (m, 2H), 3.44 – 3.30 (m, 4H), 1.74 – 1.61 (m, 2H), 1.51 – 1.39 (m, 2H), 1.40 – 1.20 (m, 4H).

^{13}C NMR (101 MHz, DMSO-d_6) δ [ppm] = 165.23, 142.19, 130.95, 129.06, 118.85, 70.17, 69.62, 69.40, 68.84, 45.35, 39.21, 32.00, 29.07, 26.12, 24.91.

HRMS (ESI) calc. for $C_{17}H_{25}ClN_4NaO_3^+$ [M+Na]$^+$: 391.1507; found 391.1511.

1.3.5.1 N-(2-(2-((6-chlorohexyl)oxy)ethoxy)ethyl)-4-vinylbenzamide (CA-Vbn)

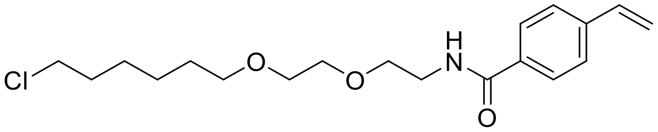

Reaction was conducted according to general procedure A using CA-NH$_2$ and 4-vinylbenzoic acid (4.8 mg, 32.6 µmol) to obtain 7.5 mg (21.2 µmol) of the desired product as a colorless oil in 72% yield.

^{1}H NMR (400 MHz, DMSO-d_6) δ [ppm] = 8.49 (t, J = 5.6 Hz, 1H), 7.83 (d, J = 8.2 Hz, 2H), 7.55 (d, J = 8.2 Hz, 2H), 6.78 (dd, J = 17.7, 10.9 Hz, 1H), 5.94 (d, J = 17.7 Hz, 1H), 5.36 (d, J = 10.9 Hz, 1H), 3.62 – 3.57 (m, 2H), 3.55 – 3.51 (m, 4H), 3.49 – 3.45 (m, 2H), 3.44 – 3.37 (m, 4H), 1.73 – 1.59 (m, 2H), 1.50 – 1.40 (m, 2H), 1.40 – 1.24 (m, 4H).

^{13}C NMR (101 MHz, DMSO-d_6) δ [ppm] = 166.28, 140.15, 136.39, 134.03, 127.99, 126.41, 116.59, 70.66, 70.11, 69.88, 69.33, 45.84, 39.67, 32.48, 29.55, 26.59, 25.39.

HRMS (ESI) calc. for $C_{19}H_{28}ClNNaO_3^+$ [M+Na]$^+$: 376.1650; found 376.1640.

1.3.6 ((1*R*,8*S*,9*s*)-bicyclo[6.1.0]non-4-yn-9-yl)methyl (2-(2-((6-chlorohexyl)oxy)ethoxy)ethyl)carbamate (CA-BCN)

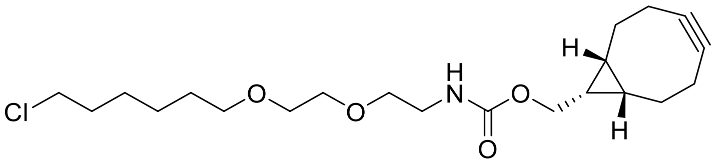

BCN-NHS (14.0 mg, 47.6 µmol, 1.1 eq) was dissolved in 500 µL DMSO. DIPEA (71.4 µL, 432 µmol, 10 equiv.) was added followed by CA-NH$_2$ (14.0 mg, 43.2 µmol, 1.0 equiv.) solubilized in DMSO. The solution was stirred for 30 min. The crude product was purified by preparative HPLC eluted with MeCN / H_2O (0.1% TFA) (50% - 90% MeCN over 60 min) to obtain 11.9 mg (29.8 µmol) of the product as a clear oil in 69% yield after lyophilization.

^{1}H NMR (400 MHz, DMSO-d6): δ = 7.07 (t, J=5.7, 1H), 4.03 (d, J=8.0, 2H), 3.62 (t, J=6.6, 2H), 3.52 – 3.44 (m, 4H), 3.38 (dt, J=11.3, 6.3, 4H), 3.11 (q, J=6.0, 2H), 2.30 – 2.06 (m, 6H), 1.78 – 1.64 (m, 2H), 1.59 – 1.42 (m, 4H), 1.41 – 1.19 (m, 4H), 0.95 – 0.78 (m, 2H).

^{13}C NMR (100 MHz, DMSO-d_6): δ = 156.4, 99.0, 70.2, 69.5, 69.4, 69.1, 61.3, 45.4, 40.1, 32.0, 29.1, 28.6, 26.1, 24.9, 20.8, 19.5, 17.6.

HRMS (ESI) calc. for [M+H]$^+$: 400.2249, found 400.2250.

1.3.6.1 Cyclooct-2-yn-1-yl (2-(2-((6-chlorohexyl)oxy)ethoxy)ethyl)carbamate (CA-SCO)

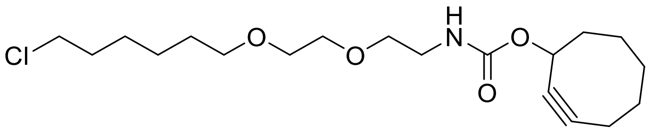

CA-NH$_2$ (15 mg, 44.4 µmol, 1.3 equiv.) was dissolved in dry DMSO (0.15 mL) and a solution of cyclooct-2-yn-1-yl (4-nitrophenyl) carbonate (10 mg, 34.2 µmol, 1.0 equiv.) in dry DMF (0.4 mL) was added followed by DIPEA (58 µL, 348 µmol: 10.2 equiv.). The reaction mixture was stirred at r.t. for 1h. The resulted mixture was acidified with 50 µL of acetic acid and afterwards purified by semi-preparative HPLC eluted with MeCN / H$_2$O (0.1% TFA) (15% MeCN for 2 min., then 15 - 100% MeCN over 25 min., followed by 100% MeCN for 15 min.) to give 8.7 mg (23.3 µmol) of the desired product as a colorless oil in 68% yield after lyophilization.

^{1}H NMR (400 MHz, DMSO-*d*$_6$) *δ* [ppm] = 7.18 (t, *J* = 5.9 Hz, 1H), 5.18 – 5.09 (m, 1H), 3.62 (t, *J* = 6.6 Hz, 2H), 3.50 – 3.43 (m, 4H), 3.40 – 3.34 (m, 4H), 3.09 (q, *J* = 5.9 Hz, 2H), 2.30 – 2.00 (m, 3H), 1.93 – 1.78 (m, 3H), 1.76 – 1.65 (m, 3H), 1.64 – 1.54 (m, 2H), 1.53 – 1.43 (m, 3H), 1.42 – 1.25 (m, 4H).

^{13}C NMR (101 MHz, DMSO-*d*$_6$) *δ* [ppm] = 155.29, 100.82, 91.79, 70.19, 69.53, 69.42, 68.99, 65.70, 45.38, 41.59, 40.07, 33.85, 32.03, 29.21, 29.06, 26.13, 25.78, 24.94, 19.95.

HRMS (ESI) calc. for $C_{19}H_{32}ClNNaO_4^+$ [M+Na]$^+$; 396.1912; found 396.1923.

1.3.7 *N*-(2-(2-((6-chlorohexyl)oxy)ethoxy)ethyl)acetamide (CA-Ac)

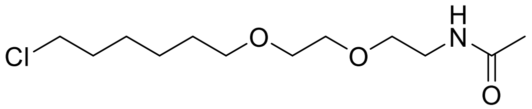

Tert-butyl (2-(2-((6-chlorohexyl)oxy)ethoxy)ethyl)carbamate (301 mg, 0.93 µmol, 1.0 equiv.) was deprotected by addition of TFA (2 mL) and afterwards dried under a stream of pressured air for 15 min. DIPEA (307 µL, 1.86 mmol, 2.0 equiv.) and DMSO (333 µL) were added followed by dropwise addition of acetic anhydride (131 µL, 1.39 mmol, 1.5 equiv.) while stirring. The reaction was stirred at r.t for 1 h. The mixture was quenched with saturated solution of NaHCO$_3$ (20 mL) and extracted with DCM (3 × 20 mL). The combined organic layers were washed with brine and dried over MgSO4. All volatiles were evaporated and the crude product was purified over normal phase flash chromatography (MeOH: DCM = 2% : 98% to 3% : 97%). The fractions containing the product were combined to give 238 mg (896 µmol) of the desired product as a colorless oil in 97% yield after evaporation.

^{1}H NMR (400 MHz, CDCl$_3$) *δ* [ppm] = 6.05 (s, 1H), 3.67 – 3.38 (m, 12H), 1.98 (s, 3H), 1.83 – 1.71 (m, 2H), 1.61 (p, *J* = 6.8 Hz, 2H), 1.52 – 1.31 (m, 4H).

^{13}C NMR (101 MHz, CDCl$_3$) *δ* [ppm] = 169.92, 71.09, 70.07, 69.83, 69.60, 44.84, 39.10, 32.32, 29.28, 26.49, 25.24, 23.10.

HRMS (ESI) calc. for $C_{12}H_{25}ClNO_3^+$ [M+H]$^+$: 266.1517; found 266.1518.

1.3.1 5-((2-(2-((6-chlorohexyl)oxy)ethoxy)ethyl)carbamoyl)-2-(6-(dimethylamino)-3-(dimethyliminio)-3H-xanthen-9-yl)benzoate (CA-5-TMR)

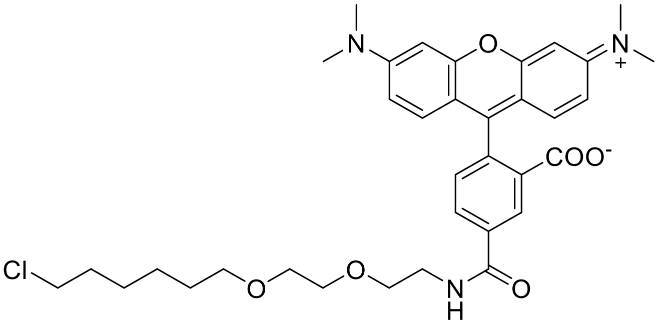

To a solution of TMR-5-COOH (2.5 mg, 5.81 µmol, 1.0 equiv.) in dry DMSO (500 µL), benzotriazolyloxytris(dimethylamino)-phosphonium hexafluorophosphat (BOP) (0.5 M in DMSO, 16.4 µL, 8.21 µmol, 1.5 equiv.) was added and the reaction was shaken at 500 rpm and r.t. for 5 min. DIPEA (3.84 µL, 23.2 µmol, 4.0 equiv.) and CA-NH$_2$ (1 M in DMSO, 8.71 µL, 8.71 µmol, 1.5 equiv.) were added and the reaction was shaken at 500 rpm and r.t. for 4 h. The crude product was acidified with acetic acid and purified over preparative HPLC eluted with MeCN / H$_2$O (0.1% FA) (10% - 90% MeCN over 50 min) to give 1.2 mg (1.89 µmol) of the desired product in 33% yield after lyophilization.

HRMS (ESI): calc. for $C_{36}H_{44}N_2O_6Cl^+$ [M+H]$^+$: 635.2887; found 635.2882.

1.3.2 5-((2-(2-((6-chlorohexyl)oxy)ethoxy)ethyl)carbamoyl)-2-(6-(dimethylamino)-3-(dimethyliminio)-10,10-dimethyl-3,10-dihydroanthracen-9-yl)benzoate (CA-5-CPY)

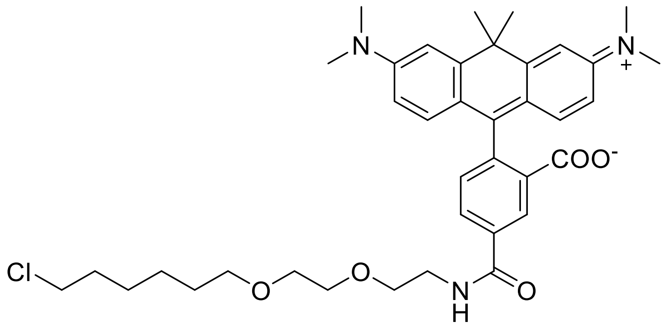

To a solution of CPY-5-COOH (2.5 mg, 5.48 µmol, 1.0 equiv.) in dry DMSO (1 mL), BOP (0.5 M in DMSO, 16.4 µL, 8.21 µmol, 1.5 equiv.) was added and the reaction was shaken at 500 rpm and r.t. for 5 min. DIPEA (3.62 µL, 21.9 µmol, 4.0 equiv.) and CA-NH_2 (1 M in DMSO, 8.21 µL, 8.21 µmol, 1.5 equiv.) were added and the reaction was shaken at 500 rpm and r.t. for 4 h. The crude product was acidified with acetic acid and purified over preparative HPLC eluted with MeCN / H_2O (0.1% FA) (10% - 90% MeCN over 50 min) to give 0.38 mg (0.57 µmol) of the desired product in 10% yield after lyophilization.

HRMS (ESI): calc. for $C_{38}H_{49}N_3O_5Cl^+$ $[M+H]^+$: 662.3360; found 662.3349.

1.3.3 1-(6-((2-(2-((6-chlorohexyl)oxy)ethoxy)ethyl)amino)-6-oxohexyl)-3,3-dimethyl-2-((E)-3-((Z)-1,3,3-trimethylindolin-2-ylidene)prop-1-en-1-yl)-3H-indol-1-ium (CA-Cy3)

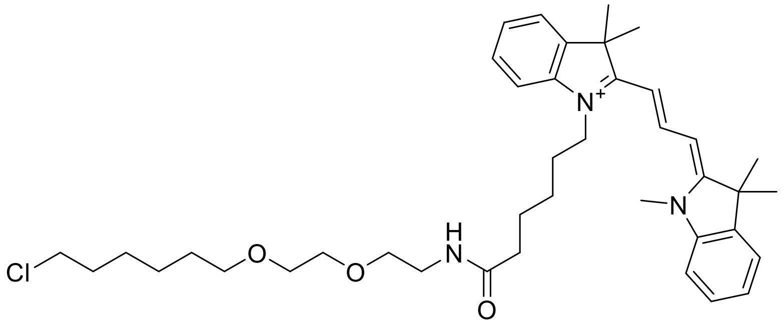

Cy3-COOH was synthesized according to Ueno et al. 2010 [3]. To a solution of Cy3-COOH (100 mg, 219 µmol, 1.0 equiv.) in dry DMSO (2 mL), DIPEA (217 µL, 1.3 mmol, 6.0 equiv.) and TSTU (92.1 mg, 306 µmol, 1.4 equiv.) were added and the reaction mixture was stirred for 10 min. at r.t. CA-NH_2 (58 mg, 262 µmol, 1.2 equiv.) in 0.5 mL DMSO was added and the reaction was stirred for 30 min, at r.t. The reaction was quenched by addition of acetic acid (230 µL) and 10% H_2O, followed by purification over preparative HPLC eluted with MeCN / H_2O (0.1% FA) (10% - 90% MeCN over 60 min) to give 102 mg (154 µmol) of the desired product in 70% yield after lyophilization.

HRMS (ESI): calc. for $C_{40}H_{57}N_3O_3Cl^+$ $[M]^+$: 662.4083; found 662.4084.

1.3.4 1-(6-((2-(2-((6-chlorohexyl)oxy)ethoxy)ethyl)amino)-6-oxohexyl)-3,3-dimethyl-2-((1E,3E)-5-((Z)-1,3,3-trimethylindolin-2-ylidene)penta-1,3-dien-1-yl)-3H-indol-1-ium (CA-Cy5)

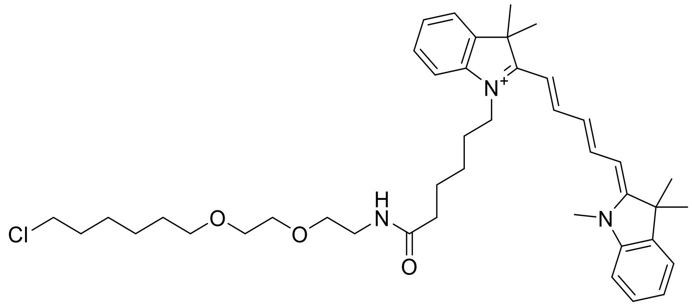

Cy5-COOH was synthesized according to Ueno et al. 2010 [3]. To a solution of Cy5-COOH (100 mg, 207 µmol, 1.0 equiv.) in dry DMSO (2 mL), DIPEA (205 µL, 1.24 mmol, 6.0 equiv.) and TSTU (87.1 mg, 289 µmol, 1.4 equiv.) were added and the reaction mixture was stirred for 10 min. at r.t. CA-NH_2 (55.5 mg, 248 µmol, 1.2 equiv.) in 0.5 mL DMSO was added and the reaction was stirred for 30 min, at r.t. The reaction was quenched by addition of acetic acid (291 µL) and 10% H_2O, followed by purification over preparative HPLC eluted with MeCN / H_2O (0.1% FA) (10% - 90% MeCN over 60 min) to give 98 mg (142 µmol) of the desired product in 69% yield after lyophilization.

HRMS (ESI): calc. for $C_{42}H_{59}N_3O_3Cl^+$ $[M]^+$: 688.4239; found 688.4239.

1.3.5 4-carboxy-2-(3-(dimethyliminio)-6-((4-methoxy-4-oxobutyl)(methyl)amino)-3H-xanthen-9-yl)benzoate (CA-TMR-biotin-1)

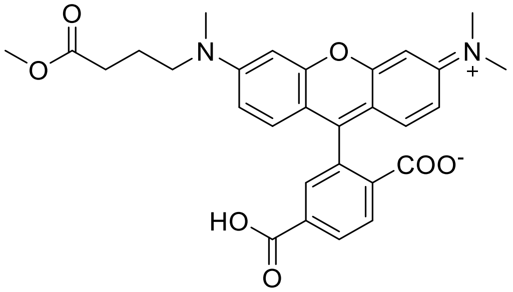

The compound was synthesized according to the procedure from Masharina et al. 2012 [15].

1.3.6 4-((2-(2-((6-chlorohexyl)oxy)ethoxy)ethyl)carbamoyl)-2-(3-(dimethyliminio)-6-((4-methoxy-4-oxobutyl)(methyl)amino)-3H-xanthen-9-yl)benzoate (CA-TMR-biotin-2)

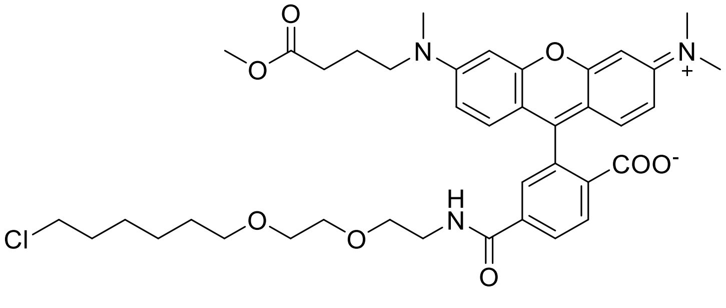

To a solution of CA-TMR-biotin-1 (17.0 mg, 32.9 µmol, 1.0 equiv.) in dry DMF, TSTU (11.9 mg, 39.5 µmol, 1.2 equiv.) and DIPEA (32.6 µL, 197 µmol, 6.0 equiv.) were added and the reaction was stirred at r.t. for 5 min. CA-NH_2 (14.7 mg, 65.8 µmol, 2.0 equiv.) was added and the reaction was stirred at r.t. for 2 h. The crude product was acidified with acetic acid and purified via preparative eluted with MeCN / H_2O (0.1% TFA) (10% - 90% MeCN over 50 min) to give 10 mg (13.8 µmol) of the desired product in 42% yield after lyophilization.

HRMS (ESI): calc. for $C_{39}H_{49}N_3O_8Cl^+$ [M+H]$^+$: 722.3208; found 722.3202.

1.3.7 2-(6-((3-carboxypropyl)(methyl)amino)-3-(dimethyliminio)-3H-xanthen-9-yl)-4-((2-(2-((6-chlorohexyl)oxy)ethoxy)ethyl)carbamoyl)benzoate (CA-TMR-biotin-3)

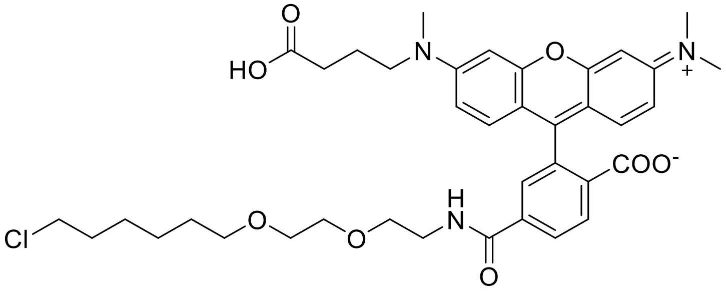

To a solution of CA-TMR-biotin-2 (8.0 mg, 11.1 µmol, 1.0 equiv.) in THF:H_2O (4:1), lithium hydroxide (1M in H_2O, 22.2 µL, 22.2 µmol, 2.0 equiv.) was added and the reaction was stirred at r.t. for 6 h. The crude product was acidified with acetic acid and purified via preparative HPLC eluted with MeCN / H_2O (0.1% TFA) (10% - 90% MeCN over 50 min) to give 6.3 mg (8.9 µmol) of the desired product in 80% yield after lyophilization.
HRMS (ESI): calc. for $C_{39}H_{49}N_3O_8Cl^+$ [M+H]$^+$: 708.3051; found 708.3049.

1.3.8 4-((2-(2-((6-chlorohexyl)oxy)ethoxy)ethyl)carbamoyl)-2-(3-(dimethyliminio)-6-((4,18-dioxo-22-((3aR,4R,6aS)-2-oxohexahydro-1H-thieno[3,4-d]imidazol-4-yl)-8,11,14-trioxa-5,17-diazadocosyl)(methyl)amino)-3H-xanthen-9-yl)benzoate (CA-TMR-biotin)

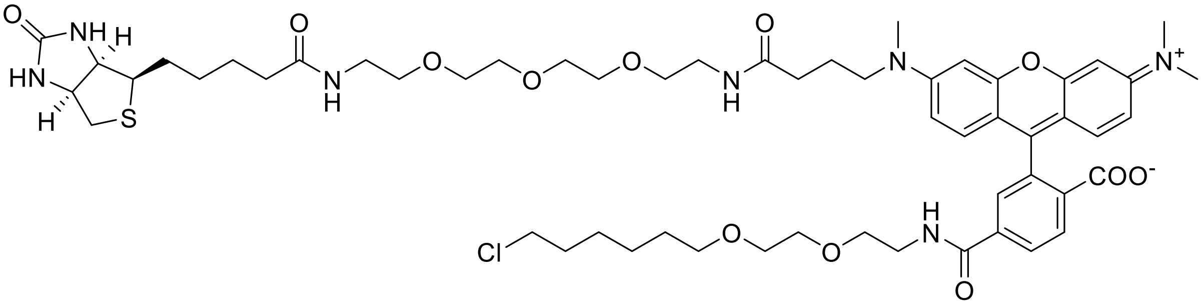

To a solution of CA-TMR-biotin-3 (6.0 mg, 8.47 µmol, 1.0 equiv.) in dry DMF, TSTU (3.06 mg, 10.2 µmol, 1.2 equiv.) and DIPEA (8.4 µL, 50.8 µmol, 6.0 equiv.) were added and the reaction was stirred at r.t. for 5 min. Biotin-PEG3-NH_2 (7.09 mg, 16.9 µmol, 2.0 equiv.) was added and the reaction was stirred at r.t. for another 2 h. The crude product was acidified with acetic acid and purified via preparative HPLC eluted with MeCN / H_2O (0.1% TFA) (10% - 90% MeCN over 50 min) to give 6.2 mg (5.6 µmol) of the desired product in 66% yield after lyophilization.
HRMS (ESI): calc. for $C_{56}H_{80}N_7O_{12}ClS^{2+}$ [M+2H]$^{2+}$: 554.7628; found 554.7632.

1.4 SNAP substrates based on benzylguanine (BG)

1.4.1 *N*-(4-(((2-amino-9*H*-purin-6-yl)oxy)methyl)benzyl)-2-azidoacetamide (BG-N_3)

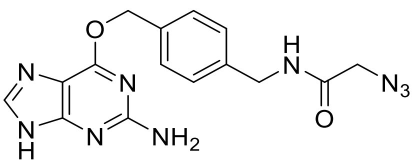

Reaction was conducted according to general procedure A using BG-NH_2 and 2-azidoacetic acid (40.7 µmol; 5.7 µL) and 11.1 mg (23.8 µmol) of the desired product were obtained as a colorless TFA-salt in 64% yield.

^{1}H NMR (400 MHz, DMSO-d_6) δ [ppm] = 8.65 (t, J = 5.9 Hz, 1H), 8.34 (s, 1H), 7.55 – 7.44 (m, 2H), 7.36 – 7.28 (m, 2H), 5.52 (s, 2H), 4.31 (d, J = 5.9 Hz, 2H), 3.89 (s, 2H).

HRMS (ESI) calc. for $C_{15}H_{16}N_9O_2^+$ [M+H]$^+$: 354.1421; found 354.1423.

1.4.2 *N*-(4-(((2-amino-9*H*-purin-6-yl)oxy)methyl)benzyl)-2-((1*S*,4*S*)-bicyclo[2.2.1]hept-5-en-2-yl)acetamide (BG-Nor2)

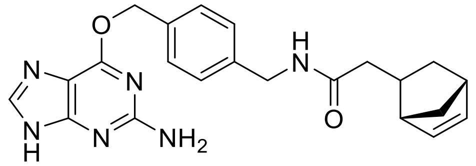

Reaction was conducted according to general procedure A with a reduced reaction time of 15 min. using BG-NH_2 and 2-((1*S*,4*S*)-bicyclo[2.2.1]hept-5-en-2-yl)acetic acid (40.7 µmol; 7.0 µL) resulting in 15.9 mg (30.7 µmol) of the desired product as a colorless TFA-salt in 83% yield.

^{1}H NMR (400 MHz, DMSO-d_6) δ [ppm] = 8.47 (s, 1H), 8.29 (t, J = 6.0 Hz, 1H), 7.49 (d, J = 8.1 Hz, 2H), 7.27 (d, J = 8.1 Hz, 2H), 6.16 (dd, J = 5.7, 3.0 Hz, 1H), 5.96 (dd, J = 5.7, 2.9 Hz, 1H), 5.53 (s, 2H), 4.25 (d, J = 6.0 Hz, 2H), 2.77 – 2.69 (m, 2H), 2.45 – 2.34 (m, 1H), 1.95 (dd, J = 13.8, 7.6 Hz, 1H), 1.90 – 1.77 (m, 2H), 1.33 – 1.26 (m, 1H), 1.25 – 1.19 (m, 1H), 0.50 (m, J = 11.4, 4.5, 2.5 Hz, 1H).

^{13}C NMR (101 MHz, DMSO-d_6) δ [ppm] = 171.71, 158.83, 158.03, 153.44, 140.89, 140.30, 137.07, 133.90, 132.45, 128.84, 127.13, 68.12, 49.10, 45.26, 42.09, 41.71, 40.67, 35.15, 31.47.

HRMS (ESI) calc. for $C_{22}H_{25}N_6O_2^+$ [M+H]$^+$: 405.2034; found 405.2034.

1.4.3 *N*-(4-(((2-amino-9*H*-purin-6-yl)oxy)methyl)benzyl)-2-(4-(6-methyl-1,2,4,5-tetrazin-3-yl)phenyl)acetamide (BG-Tz)

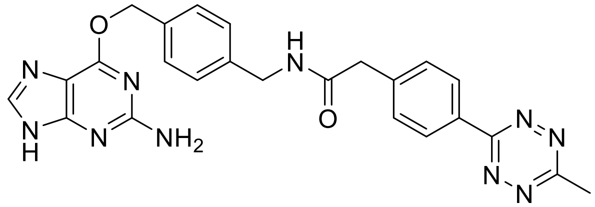

Reaction was conducted according to general procedure A using BG-NH_2 and 2-(4-(6-methyl-1,2,4,5-tetrazin-3-yl)phenyl)acetic acid (40.7 µmol, 9.4 mg) to give 12.4 mg (17.0 µmol) of the desired product as a rose TFA-salt in 56% yield.

^{1}H NMR (400 MHz, DMSO-d_6) δ [ppm] = 8.70 (t, J = 5.9 Hz, 1H), 8.45 – 8.39 (m, 2H), 8.37 (s, 1H), 7.60 – 7.53 (m, 2H), 7.52 – 7.44 (m, 2H), 7.33 – 7.26 (m, 2H), 5.51 (s, 2H), 4.31 (d, J = 5.9 Hz, 2H), 3.64 (s, 2H), 2.99 (s, 3H), 2.54 (s, 3H).

^{13}C NMR (101 MHz, DMSO-d_6) δ [ppm] = 170.04, 167.53, 163.69, 159.37, 158.85, 158.53, 154.36, 141.59, 140.96, 140.84, 140.19, 134.78, 130.62, 130.61, 129.34, 127.81, 68.28, 42.69, 40.90, 21.31.

HRMS (ESI) calc. for $C_{24}H_{23}N_{10}O_2^+$ [M+H]$^+$: 483.2000; found 483.2006.

1.4.4 *N*-(4-(((2-amino-9*H*-purin-6-yl)oxy)methyl)benzyl)-4-azidobenzamide (BG-PhN_3)

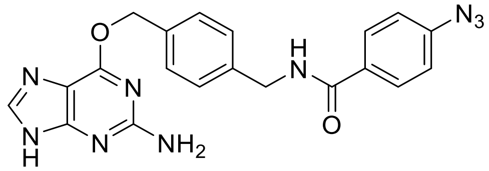

Reaction was conducted according to general procedure A with a reduced reaction time of 15 min. using BG-NH_2 and 4-azidobenzoic acid (6.6 mg, 40.7 µmol) to obtain 15.5 mg (29.3 µmol) of the desired product as a colorless TFA-salt in 79% yield.

^{1}H NMR (400 MHz, DMSO-*d_6*) *δ* [ppm] = 9.10 (t, *J* = 5.9 Hz, 1H), 8.38 (s, 1H), 7.99 – 7.89 (m, 2H), 7.55 – 7.46 (m, 2H), 7.38 – 7.33 (m, 2H), 7.25 – 7.16 (m, 2H), 5.52 (s, 2H), 4.48 (d, *J* = 5.9 Hz, 2H).

^{13}C NMR (101 MHz, DMSO-*d_6*) *δ* [ppm] = 165.27, 158.91, 158.61, 158.26, 153.77, 142.36, 140.57, 140.02, 134.19, 130.81, 129.15, 128.89, 127.36, 118.96, 67.94, 42.48.

HRMS (ESI) calc. for $C_{20}H_{18}N_9O_2^+$ $[M+H]^+$: 416.1578; found 416.1577.

1.4.5 *N*-(4-(((2-amino-9*H*-purin-6-yl)oxy)methyl)benzyl)-4-vinylbenzamide (BG-VBn)

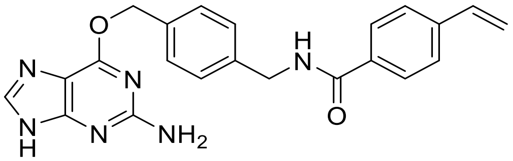

Reaction was conducted according to general procedure A with a reduced reaction time of 15 min. using BG-NH_2 and 4-vinylbenzoic acid (40.7 µmol; 6.5 mg) to obtain 14.7 mg (28.6 µmol) of the desired product as a colorless TFA-salt in 77% yield.

^{1}H NMR (400 MHz, DMSO-*d_6*) *δ* [ppm] = 9.09 (t, *J* = 6.0 Hz, 1H), 8.44 (s, 1H), 7.87 (d, *J* = 8.3 Hz, 2H), 7.57 (d, *J* = 8.3 Hz, 2H), 7.51 (d, *J* = 7.9 Hz, 2H), 7.36 (d, *J* = 7.9 Hz, 2H), 6.79 (dd, *J* = 17.7, 11.0 Hz, 1H), 5.95 (d, *J* = 17.7 Hz, 1H), 5.53 (s, 2H), 5.37 (d, *J* = 11.0 Hz, 1H), 4.49 (d, *J* = 6.0 Hz, 2H).

^{13}C NMR (101 MHz, DMSO-*d_6*) *δ* [ppm] = 165.81, 158.85, 158.62, 158.28, 153.55, 140.79, 140.13, 139.84, 135.91, 134.06, 133.43, 128.92, 127.61, 127.35, 126.03, 116.24, 68.09, 42.46.

HRMS (ESI) calc. for $C_{22}H_{21}N_6O_2^+$ $[M+H]^+$: 401.1721; found 401.1707.

1.4.6 ((1R,8S,9s)-bicyclo[6.1.0]non-4-yn-9-yl)methyl (4-(((2-amino-9*H*-purin-6-yl)oxy)methyl)benzyl)carbamate (BG-BCN)

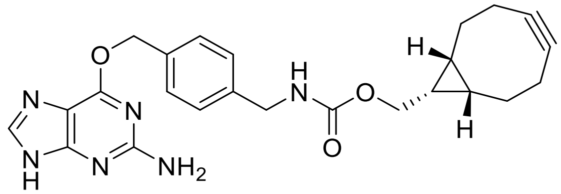

A solution of ((1*R*,8*S*,9*s*)-bicyclo[6.1.0]non-4-yn-9-yl)methyl (2,5-dioxopyrrolidin-1-yl) carbonate (10 mg, 34.3 µmol, 1.0 equiv.) in dry DMSO (0.4 mL) was added to a solution of 10.2 mg BG-NH_2 (37.8 µmol, 1.1 equiv.) in dry DMSO (0.1 mL) followed by 28.4 µL of DIPEA (172 µmol, 5 equiv.). The reaction was stirred at r.t. for 30 min. The resulted mixture was acidified with acetic acid (3 µL) and H_2O (53 µL), then purified by semi-preparative HPLC eluted with MeCN / H_2O (0.1% TFA) (10% MeCN for 10 min., then 10 - 90% MeCN over 55 min. followed by 99% MeCN for 5 min.) to give 14.0 mg (31.4 µmol) of the desired product as a colorless solid in 91% yield after lyophilization.

^{1}H NMR (400 MHz, DMSO-*d_6*) *δ* [ppm] = 8.36 (s, 1H), 7.70 (t, *J* = 6.2 Hz, 1H), 7.52 – 7.46 (m, 2H), 7.28 (d, *J* = 8.0 Hz, 2H), 5.51 (s, 2H), 4.18 (d, *J* = 6.0 Hz, 2H), 4.06 (d, *J* = 8.0 Hz, 2H), 2.28 – 2.07 (m, 6H), 1.52 (d, *J* = 12.4 Hz, 2H), 1.28 (dt, *J* = 18.3, 9.1 Hz, 1H), 0.86 (t, *J* = 9.8 Hz, 2H).

HRMS (ESI) calc. for $C_{24}H_{27}N_6O_3^+$ $[M+H]^+$: 447.2139; found 447.2135.

1.4.6.1 *N*-(4-(((2-amino-9*H*-purin-6-yl)oxy)methyl)benzyl)acetamide (BG-Ac)

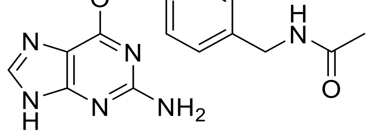

BG-NH_2 (300 mg, 1.11 mmol, 1.0 equiv.) was dissolved in dry DMSO (2.5 mL) and 367 µL of DIPEA (2.22 mmol, 2.0 equiv.) was added followed by dropwise addition of acetic anhydride (156 µL, 1.66 mmol, 1.5 equiv.) while stirring. The reaction mixture was stirred at r.t.

for 1 h. Afterwards, the reaction was quenched with acetic acid (387 µL) and H_2O (341 µL) followed by centrifugation at 3'000 rpm for 3 min. The pellet was washed twice with H_2O and afterwards lyophilized to obtain 190 mg (608 µmol) of the desired product as a colorless solid in 55% yield.

^{1}H NMR (400 MHz, DMSO-d_6) δ [ppm] = 8.30 (s, 1H), 7.45 (d, J = 8.1 Hz, 2H), 7.27 (d, J = 7.9 Hz, 2H), 6.82 (s, 2H), 5.48 (s, 2H), 4.24 (d, J = 5.9 Hz, 2H), 1.86 (s, 3H).

HRMS (ESI) calc. for $C_{15}H_{17}N_6O_2^+$ $[M+H]^+$: 313.1408; found 313.1406.

1.4.7 Cyclooct-2-yn-1-yl (4-(((2-amino-9*H*-purin-6-yl)oxy)methyl)benzyl)carbamate (BG-SCO)

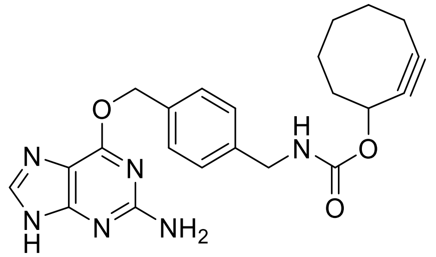

BG-NH_2 (10 mg, 37.0 µmol, 1.0 equiv.) was dissolved in dry DMSO (0.5 mL) and a solution of cyclooct-2-yn-1-yl (4-nitrophenyl) carbonate (10.7 mg, 37 µmol, 1.0 equiv.) in dry DMF (0.4 mL) was added followed by DIPEA (30.6 µL, 142 µmol: 5.0 equiv.). The reaction mixture was stirred at r.t. for 1 h. The resulted mixture was acidified with acetic acid (25 µL) and afterwards purified by semi-preparative HPLC eluted with MeCN / H_2O (0.1% TFA) (15% MeCN for 5 min., then 15 - 100% MeCN over 25 min., followed by 100% MeCN for 15 min.) to give 16 mg (23.3 µmol) of the desired product as a colorless TFA-salt in 81% yield after lyophilization.

^{1}H NMR (400 MHz, DMSO-d_6) δ [ppm] = 8.44 (s, 1H), 7.81 (t, J = 6.1 Hz, 1H), 7.50 (d, J = 8.0 Hz, 2H), 7.28 (d, J = 8.0 Hz, 2H), 5.52 (s, 2H), 5.20 – 5.11 (m, 1H), 4.17 (d, J = 6.1 Hz, 2H), 2.30 – 2.02 (m, 3H), 1.96 – 1.85 (m, 1H), 1.89 – 1.76 (m, 2H), 1.76 – 1.64 (m, 1H), 1.64 – 1.53 (m, 2H), 1.55 – 1.41 (m, 1H).

^{13}C NMR (101 MHz, DMSO-d_6) δ [ppm] = 158.86, 158.10, 155.53, 153.58, 140.80, 140.06, 134.15, 128.90, 127.16, 107.66, 100.97, 91.76, 68.06, 65.95, 43.50, 41.58, 33.85, 29.21, 25.79, 19.95.

HRMS (ESI) calc. for $C_{22}H_{24}N_6NaO_3^+$ $[M+Na]^+$: 443.1802; found 443.1797.

1.4.8 5-((4-(((2-amino-9H-purin-6-yl)oxy)methyl)benzyl)carbamoyl)-2-(6-(dimethylamino)-3-(dimethyliminio)-2,3,4,4a-tetrahydro-1H-xanthen-9-yl)benzoate (BG-5-TMR)

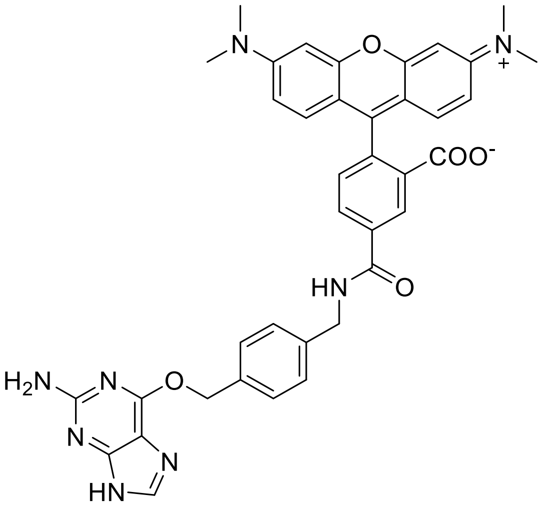

TSTU (1.45 mg, 4.82 µmol, 1.2 equiv.) was dissolved in dry DMSO-d_6 (500 µL). TMR-5-COOH (1.15 mg, 2.68 µmol, 1.0 equiv.) was dissolved in the TSTU solution and DIPEA (1.77 µL, 10.7 µmol, 4.0 equiv.) was added. The mixture was stirred at r.t. for 10 min. BG-NH_2 (1.08 mg, 4.01 µmol, 1.5 equiv.) was dissolved in dry DMSO-d_6 (200 µL) and added to the reaction. The reaction mixture was

stirred at r.t. for 1 h. The compound was purified over preparative HPLC eluted with MeCN / H_2O (0.1% TFA) (10% MeCN for 10 min., then 10 - 90% MeCN over 40 min., followed by 90% MeCN for 5 min.) to give after lyophilization 378 µg (554 nmol) of the desired product in 21% yield.

HRMS (ESI): calc. for $C_{38}H_{37}N_8O_5$ $[M+2H]^{2+}$: 342.1399; found 342.1394.

^{1}H NMR (TMR-5-COOH) (400 MHz, DMSO-*d*$_6$) *δ* [ppm] = 8.39 (s, *J* = 1.5 Hz, 1H), 8.28 (dd, *J* = 8.1, 1.5 Hz, 1H), 7.33 (d, *J* = 8.0 Hz, 1H), 6.58 – 6.45 (m, 6H), 2.95 (s, 12H).

^{13}C NMR (TMR-5-COOH) (101 MHz, DMSO-*d*$_6$) *δ* [ppm] = 168.31, 166.09, 152.03, 135.96, 132.76, 128.50, 109.05, 97.95, 40.15, 39.99, 39.79.

1.4.9 5-((4-(((2-amino-9H-purin-6-yl)oxy)methyl)benzyl)carbamoyl)-2-(6-(dimethylamino)-3-(dimethyliminio)-10,10-dimethyl-1,2,3,4,4a,10-hexahydroanthracen-9-yl)benzoate (BG-5-CPY)

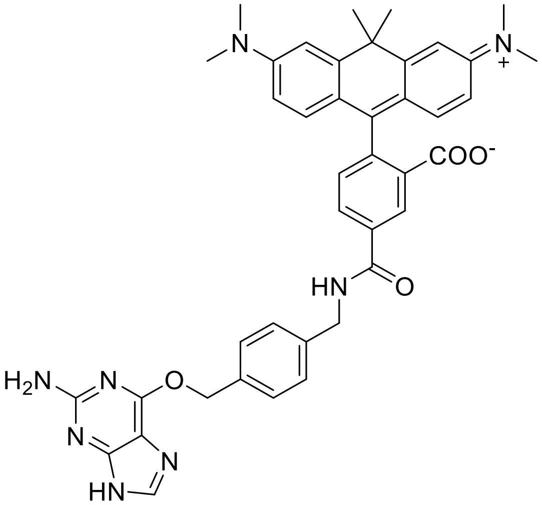

TSTU (1.44 mg, 4.78 µmol, 1.2 equiv.) was dissolved in dry DMSO-*d*$_6$ (500 µL). CPY-5-COOH (2.0 mg, 4.38 µmol, 1.1 equiv.) was dissolved in the TSTU solution and DIPEA (2.63 µL, 15.9 µmol, 4 equiv.) was added. The mixture was stirred at r.t. for 10 min. BG-NH_2 (1.08 mg, 3.98 µmol, 1.5 equiv.) was dissolved in dry DMSO-*d*$_6$ (200 µL) and added to the reaction. The reaction mixture was stirred at r.t. for 1 h. The compound was purified over preparative HPLC eluted with MeCN / H_2O (0.1% TFA) (10% MeCN for 10 min., then 10 - 90% MeCN over 40 min., followed by 90% MeCN for 5 min.) to give 346 µg (488 nmol) of the desired product in 18% yield after lyophilization.

HRMS (ESI): calc. for $C_{41}H_{42}N_8O_4$ $[M+2H]^{2+}$: 355.1659; found 355.1659.

1.4.9.1 1-(6-((4-(((2-amino-9H-purin-6-yl)oxy)methyl)benzyl)amino)-6-oxohexyl)-3,3-dimethyl-2-((E)-3-((Z)-1,3,3-trimethylindolin-2-ylidene)prop-1-en-1-yl)-3H-indol-1-ium (BG-Cy3)

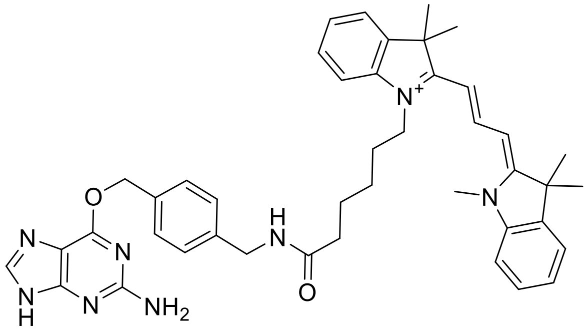

Cy3-COOH was synthesized according to Ueno et al. 2010 [3]. To a solution of Cy3-COOH (100 mg, 219 µmol, 1.0 equiv.) in dry DMSO (1.5 mL), DIPEA (217 µL, 1.3 mmol, 6.0 equiv.) and TSTU (92.1 mg, 306 µmol, 1.4 equiv.) were added and the reaction mixture was stirred for 10 min. at r.t. BG-NH_2 (70.9 mg, 262 µmol, 1.2 equiv.) was added and the reaction was stirred for 30 min. at r.t. The reaction

was quenched by addition of acetic acid (230 µL) and 10% H_2O, followed by purification over preparative HPLC eluted with MeCN / H_2O (0.1% FA) (10% - 90% MeCN over 60 min.) to give. 28.5 mg (40.1 µmol) of the desired product in 18% yield after lyophilization.
HRMS (ESI): calc. for $C_{43}H_{50}N_8O_2^{2+}$ $[M+H]^{2+}$: 355.2023; found 355.2022.

1.4.10 1-(6-((4-(((2-amino-9H-purin-6-yl)oxy)methyl)benzyl)amino)-6-oxohexyl)-3,3-dimethyl-2-((1E,3E)-5-((Z)-1,3,3-trimethylindolin-2-ylidene)penta-1,3-dien-1-yl)-3H-indol-1-ium (BG-Cy5)

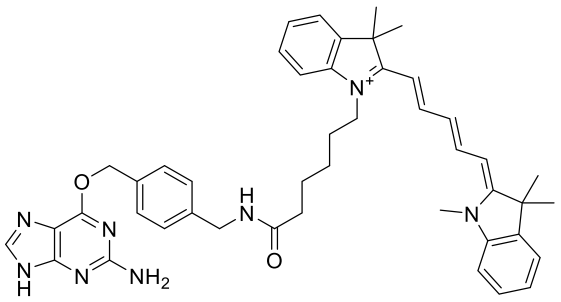

Cy5-COOH was synthesized according to Ueno et al. 2010[3]. To a solution of Cy5-COOH (50.0 mg, 103 µmol, 1.0 equiv.) in dry DMSO (1.5 mL), DIPEA (103 µL, 620 µmol, 6.0 equiv.) and TSTU (43.6 mg, 145 µmol, 1.4 equiv.) were added and the reaction mixture was stirred for 10 min. at r.t. BG-NH_2 (33.5 mg, 124 µmol, 1.2 equiv.) was added and the reaction was stirred for 30 min. at r.t. The reaction was quenched by addition of acetic acid (109 µL) and 10% H_2O, followed by purification over preparative HPLC eluted with MeCN / H_2O (0.1% FA) (10% - 90% MeCN over 60 min.) to give 45 mg (61.1 µmol) of the desired product in 59% yield after lyophilization.
HRMS (ESI): calc. for $C_{45}H_{52}N_8O_2^{2+}$ $[M+H]^{2+}$: 368.2101; found 368.2102.

1.5 SNAP substrates based on chloropyrimidine (CP)

1.5.1 *N*-(4-(((2-amino-6-chloropyrimidin-4-yl)oxy)methyl)benzyl)-2-azidoacetamide (CP-N_3)

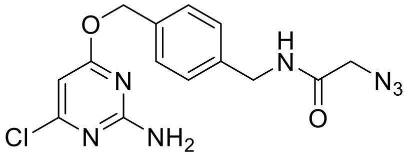

Reaction was conducted according to general procedure A using CP-NH_2 and 2-azidoacetic acid (5.8 µL, 41.6 µmol) to obtain 10.1 mg (21.9 µmol) of the desired product as a colorless TFA-salt in 58% yield.
^{1}H NMR (400 MHz, DMSO-*d*$_6$) *δ* [ppm] = 8.62 (t, *J* = 5.8 Hz, 1H), 7.40 (d, *J* = 7.7 Hz, 2H), 7.28 (d, *J* = 7.7 Hz, 2H), 6.13 (s, 1H), 5.29 (s, 2H), 4.30 (d, *J* = 5.8 Hz, 2H), 3.88 (s, 2H).
^{13}C NMR (101 MHz, DMSO-*d*$_6$) *δ* [ppm] = 170.28, 167.32, 162.77, 160.01, 138.90, 134.90, 128.44, 127.46, 94.42, 67.21, 50.78, 42.01.
HRMS (ESI) calc. for $C_{14}H_{15}ClN_7O_2^+$ $[M+H]^+$: 348.0970; found 348.0971.

1.5.2 *N*-(4-(((2-amino-6-chloropyrimidin-4-yl)oxy)methyl)benzyl)-2-((1*S*,4*S*)-bicyclo[2.2.1]hept-5-en-2-yl)acetamide (CP-Nor2)

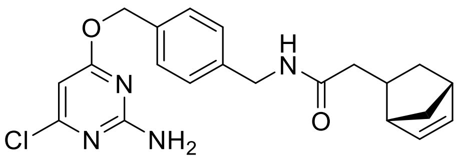

Reaction was conducted according to general procedure A with a reduced reaction time of 15 min. using CP-NH_2 and 2-((1*S*,4*S*)-bicyclo[2.2.1]hept-5-en-2-yl)acetic acid (7.1 µL, 41.6 µmol) resulting in 14.5 mg (28.3 µmol) of the desired product as a colorless TFA-salt in 75% yield.
^{1}H NMR (400 MHz, DMSO-*d*$_6$) *δ* [ppm] = 8.25 (t, *J* = 5.9 Hz, 1H), 7.38 (d, *J* = 8.1 Hz, 2H), 7.23 (d, *J* = 8.1 Hz, 2H), 7.10 (brs, 2H), 6.16 (dd, *J* = 5.7, 3.0 Hz, 1H), 6.13 (s, 1H), 5.96 (dd, *J* = 5.7, 2.9 Hz, 1H), 5.28 (s, 2H), 4.24 (d, *J* = 5.9 Hz, 2H), 2.77 – 2.69 (m, 2H), 2.46 –

2.35 (m, 1H), 1.94 (dd, *J* = 13.8, 7.6 Hz, 1H), 1.90 – 1.76 (m, 2H), 1.35 – 1.26 (m, 1H), 1.26 – 1.18 (m, 1H), 0.50 (m, *J* = 11.4, 4.3, 2.5 Hz, 1H).

^{13}C NMR (101 MHz, DMSO-d_6) *δ* [ppm] = 171.61, 170.28, 162.75, 159.97, 139.81, 137.01, 134.53, 132.42, 128.31, 127.11, 94.39, 67.23, 49.07, 45.24, 42.06, 41.69, 40.64, 35.11, 31.45.

HRMS (ESI) calc. for $C_{21}H_{23}ClN_4NaO_2^+$ [M+Na]$^+$; 421.1402; found 421.1403.

1.5.3 *N*-(4-(((2-amino-6-chloropyrimidin-4-yl)oxy)methyl)benzyl)-2-(4-(6-methyl-1,2,4,5-tetrazin-3-yl)phenyl)acetamide (CP-Tz)

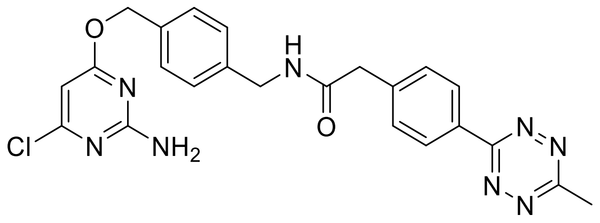

Reaction was conducted according to general procedure A using CP-NH_2 and 2-(4-(6-methyl-1,2,4,5-tetrazin-3-yl)phenyl)acetic acid (9.6 mg, 41.6 µmol). The product was purified by preparative HPLC eluted with MeCN / H_2O (0.1% TFA) (10% MeCN for 10 min., then 10 - 90% MeCN over 40 min., followed by 90% MeCN for 10 min.) to give 2.6 mg (4.4 µmol) of the desired product as a rose TFA-salt in 12% yield after lyophilization.

^{1}H NMR (400 MHz, DMSO-d_6) *δ* [ppm] = 8.66 (t, *J* = 5.9 Hz, 1H), 8.41 (d, *J* = 8.3 Hz, 2H), 7.56 (d, *J* = 8.3 Hz, 2H), 7.38 (d, *J* = 7.9 Hz, 2H), 7.26 (d, *J* = 7.9 Hz, 2H), 7.10 (s, 2H), 6.13 (s, 1H), 5.28 (s, 2H), 4.29 (d, *J* = 5.9 Hz, 2H), 3.63 (s, 2H), 2.99 (s, 3H).

^{13}C NMR (101 MHz, DMSO-d_6) *δ* [ppm] = 170.28, 169.51, 167.04, 163.21, 162.76, 159.99, 141.13, 139.32, 134.75, 130.14, 130.07, 128.42, 127.34, 94.40, 67.22, 42.21, 42.09, 20.83.

HRMS (ESI) calc. for $C_{23}H_{22}ClN_8O_2^+$ [M+H]$^+$: 477.1549; found 477.1553.

1.5.4 *N*-(4-(((2-amino-6-chloropyrimidin-4-yl)oxy)methyl)benzyl)-4-azidobenzamide (CP-PhN$_3$)

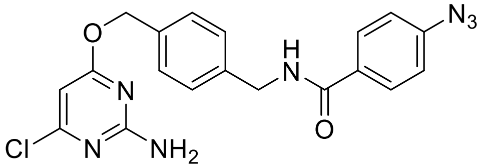

Reaction was conducted according to general procedure A with a reduced reaction time of 15 min. using BG-NH_2 and 4-azidobenzoic acid (6.8 mg, 41.6 µmol) to obtain 12.0 mg (22.9 µmol) of the desired product as a colorless TFA-salt in 61% yield.

^{1}H NMR (400 MHz, DMSO-d_6) *δ* [ppm] = 9.06 (t, *J* = 5.9 Hz, 1H), 7.94 (d, J = 8.4 Hz, 2H), 7.39 (d, *J* = 7.9 Hz, 2H), 7.32 (d, *J* = 7.9 Hz, 2H), 7.21 (d, *J* = 8.4 Hz, 2H), 7.10 (s, 2H), 5.29 (s, 2H), 4.47 (d, *J* = 5.9 Hz, 2H).

^{13}C NMR (101 MHz, DMSO-d_6) *δ* [ppm] = 170.27, 165.20, 162.75, 159.97, 142.30, 139.59, 134.66, 130.83, 129.12, 128.36, 127.29, 118.90, 94.38, 67.23, 42.44.

HRMS (ESI) calc. for $C_{19}H_{16}ClN_7NaO_2^+$ [M+Na]$^+$: 432.0946; found 432.0942.

1.5.5 *N*-(4-(((2-amino-6-chloropyrimidin-4-yl)oxy)methyl)benzyl)-4-vinylbenzamide (CP-Vbn)

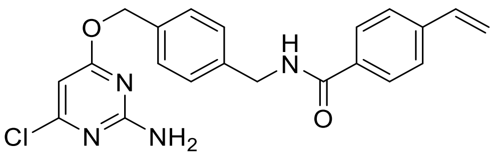

Reaction was conducted according to general procedure A with a reduced reaction time of 15 min. using CP-NH_2 and 4-vinylbenzoic acid (41.6 µmol; 6.2 mg) to obtain 11.6 mg (22.8 µmol) of the desired product as a colorless TFA-salt in 60% yield.

^{1}**H NMR** (400 MHz, DMSO-d_6) *δ* [ppm] = 9.04 (t, *J* = 6.0 Hz, 1H), 7.87 (d, *J* = 8.3 Hz, 2H), 7.57 (d, *J* = 8.3 Hz, 2H), 7.40 (d, *J* = 8.1 Hz, 2H), 7.34 (d, *J* = 8.1 Hz, 2H), 6.79 (dd, *J* = 17.7, 10.9 Hz, 1H), 7.10 (brs, 2H), 6.12 (s, 1H), 5.95 (d, *J* = 17.7 Hz, 1H), 5.37 (d, *J* = 10.9 Hz, 1H), 5.29 (s, 2H), 4.47 (d, *J* = 6.0 Hz, 2H).
^{13}**C NMR** (101 MHz, DMSO-d_6) *δ* [ppm] = 170.29, 165.77, 162.77, 159.99, 139.80, 139.68, 135.91, 134.66, 133.45, 128.41, 127.61, 127.31, 126.00, 116.20, 94.40, 67.27, 42.43.
HRMS (ESI) calc. for $C_{21}H_{20}ClN_4O_2^+$ $[M+H]^+$: 395.1269; found 395.1258.

1.5.6 4-(Benzyloxy)-6-chloropyrimidin-2-amine (CP)

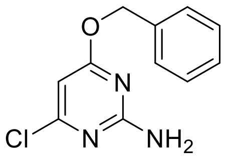

2-Amino-4,6-dichloropyrimidine (200 mg, 1.22 mmol, 1.0 equiv.) was dissolved in dry DMF (2 mL). Benzyl alcohol (63 µL, 1.22 mmol, 1.0 equiv.), KO^tBu (342.2 mg, 3.04 mmol, 2.5 equiv.) and KI (20.2 mg, 0.122 mmol, 0.1 equiv.) were added and the reaction mixture was stirred at room temperature for 4 h. Afterwards, the reaction was quenched with water and extracted with EtOAc (3 ×). The combined organic layers were washed with brine and dried over $MgSO_4$. The volatiles were evaporated and the crude product was purified over normal phase flash chromatography (hexane:DCM = 50% : 50% to 100% DCM). The fractions containing the product were combined, volatiles were evaporated and 134 mg (0.569 mmol) of the desired product was obtained as a yellowish solid in 47% yield.
^{1}**H NMR** (400 MHz, $CDCl_3$): *δ* = 7.43–7.30 (m, 5H), 6.01 (d, J = 0.7 Hz, 1H), 5.31 (s, 2H), 2.26 (s, 3H) ppm.
^{13}**C NMR** (101 MHz, $CDCl_3$): *δ* = 170.6, 168.4, 162.6, 136.7, 128.7, 128.5, 128.0, 127.4, 97.2, 93.0, 77.4, 77.1, 76.7, 67.5, 123.7 ppm.
HRMS (ESI) calc. for $C_{11}H_{11}ClN_3O^+$ $[M+H]^+$: 236.0585; found 236.0583.

1.5.7 ((1*R*,8*S*,9*s*)-bicyclo[6.1.0]non-4-yn-9-yl)methyl(4-(((2-amino-6-chloropyrimidin-4-yl)oxy)methyl)benzyl)carbamate (CP-BCN)

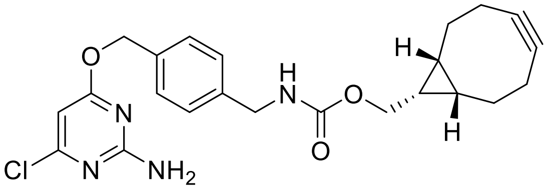

((1*R*,8*S*,9*s*)-bicyclo[6.1.0]non-4-yn-9-yl)methyl (2,5-dioxopyrrolidin-1-yl) carbonate (10.0 mg, 34.3 µmol; 1.0 equiv.) was dissolved in dry DMSO (0.5 mL) and DIPEA (28.4 µL, 172 µmol, 5 equiv.) followed by CP-NH_2 (10.0 mg, 37.8 µmol, 1.1 equiv.) were added. The reaction was stirred at r.t. for 30 min. The resulted mixture was acidified with acetic acid (3 µL) and H_2O (53 µL), then purified by preparative HPLC eluted with MeCN / H_2O (0.1% TFA) (10% MeCN for 10 min., then 10 - 90% MeCN over 55 min., followed by 99% MeCN for 5 min.) to give 1.4 mg (3.11 µmol) of the desired product as a colorless solid in 9% yield after lyophilization.
^{1}**H NMR** (400 MHz, DMSO-d_6) *δ* [ppm] = 7.68 (q, *J* = 6.4 Hz, 1H), 7.38 (d, *J* = 8.0 Hz, 2H), 7.25 (d, *J* = 7.8 Hz, 2H), 6.12 (s, 1H), 5.28 (s, 2H), 4.17 (d, *J* = 6.1 Hz, 2H), 4.06 (d, *J* = 8.0 Hz, 2H), 2.29 – 1.72 (m, 6H), 1.71 – 1.38 (m, 2H), 1.35 – 0.60 (m, 3H).
HRMS (ESI) calc. for $C_{23}H_{26}ClN_4O_3^+$ $[M+H]^+$: 441.1688; found 441.1688.

1.5.8 *N*-(4-(((2-amino-6-chloropyrimidin-4-yl)oxy)methyl)benzyl)acetamide (CP-Ac)

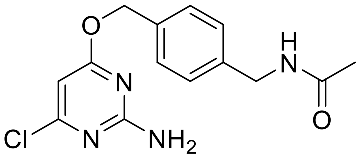

CP-NH_2 (300 mg, 1.13 mmol, 1.0 equiv.) was dissolved in dry DMSO (1.5 mL) and DIPEA (375 µL, 2.27 mmol, 2.0 equiv.) was added followed by dropwise addition of acetic anhydride (160 µL, 1.70 mmol, 1.5 equiv.) while stirring. The reaction mixture was stirred at r.t. for 1 h. Afterwards, the reaction was quenched with acetic acid (387 µL) and H_2O (341 µL) followed by purification over preparative HPLC eluted with MeCN / H_2O (0.1% TFA) (30% MeCN for 10 min., then 30 - 90% MeCN over 55 min., followed by 99% MeCN for 5 min.) to give 201 mg (655 µmol) of the desired product as a colorless solid in 58% yield after lyophilization.

^{1}H NMR (400 MHz, DMSO-*d*$_6$) *δ* [ppm] = 8.33 (t, *J* = 6.0 Hz, 1H), 7.41 – 7.35 (m, 2H), 7.28 – 7.22 (m, 2H), 7.09 (s, 2H), 6.13 (s, 1H), 5.29 (s, 2H), 4.24 (d, *J* = 5.9 Hz, 2H), 1.86 (s, 3H).

HRMS (ESI) calc. for $C_{14}H_{16}ClN_4O_2^+$ $[M+H]^+$: 307.0956; found 307.0957.

1.5.9 Cyclooct-2-yn-1-yl (4-(((2-amino-6-chloropyrimidin-4-yl)oxy)methyl)benzyl)carbamate (CP-SCO)

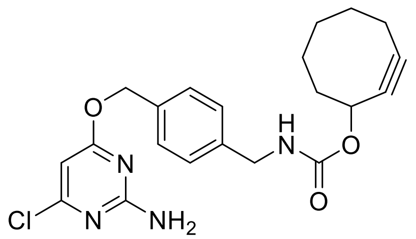

CP-NH_2 (10 mg; 37.8 µmol, 1.3 equiv.) was dissolved in dry DMF (0.3 mL) and a solution of 8.4 mg cyclooct-2-yn-1-yl (4-nitrophenyl) carbonate (29.1 µmol, 1.0 equiv.) in dry DMF (0.2 mL) was added followed by DIPEA (24.0 µL, 145 µmol: 5.0 equiv.). The reaction mixture was stirred at r.t. for 2 h. The resulted mixture was acidified with acetic acid (25 µL) and afterwards purified by semi-preparative HPLC eluted with MeCN / H_2O (0.1% TFA) (15% MeCN for 2 min., then 15 - 100% MeCN over 25 min., followed by 100% MeCN for 15 min.) to give 12.0 mg (22.7 µmol) of the desired product as a colorless TFA-salt in 78% yield after lyophilization.

^{1}H NMR (400 MHz, DMSO-*d*$_6$) *δ* [ppm] = 7.78 (t, *J* = 6.2 Hz, 1H), 7.38 (d, *J* = 8.1 Hz, 2H), 7.24 (d, *J* = 8.1 Hz, 2H), 6.13 (s, 1H), 5.28 (s, 2H), 5.21 – 5.11 (m, 1H), 4.15 (d, *J* = 6.2 Hz, 2H), 2.29 – 2.02 (m, 3H), 1.95 – 1.85 (m, 1H), 1.85 – 1.78 (m, 2H), 1.76 – 1.65 (m, 1H), 1.65 – 1.54 (m, 2H), 1.54 – 1.42 (m, 1H).

^{13}C NMR (101 MHz, DMSO-*d*$_6$) *δ* [ppm] = 170.30, 162.78, 159.99, 155.51, 139.64, 134.73, 128.40, 127.11, 100.94, 94.41, 91.76, 67.25, 65.93, 43.50, 41.58, 33.84, 29.21, 25.79, 19.96.

HRMS (ESI) calc. for $C_{21}H_{23}ClN_4NaO_3^+$ $[M+Na]^+$: 437.1351; found 437.1358.

1.5.10 2-(6-amino-3-iminio-4,5-disulfonato-3*H*-xanthen-9-yl)-4-((4-(((2-amino-6-chloropyrimidin-4-yl)oxy)methyl)benzyl)carbamoyl)benzoate (CP-Alexa488)

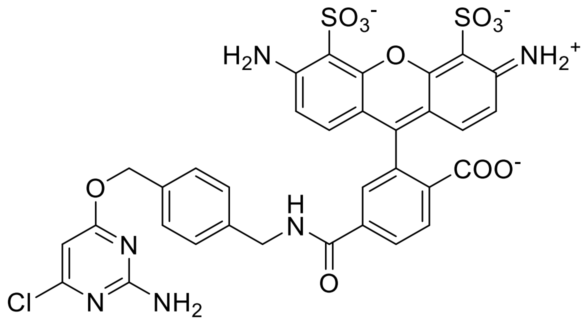

In an Eppendorf tube, CP-NH_2 (0.34 µg, 1.27 µmol, 2.0 equiv.) was dissolved in dry DMSO (100 µL) followed by addition of DIPEA (885 µL, 5.1 µmol, 8.0 equiv.) and a solution of 2-(6-amino-3-iminio-4,5-disulfonato-3H-xanthen-9-yl)-4-(((2,5-dioxopyrrolidin-1-yl)oxy)carbonyl)benzoate (0.4 mg, 0.64 µmol, 1.0 equiv.) in dry DMSO (100 µL). The reaction was kept at r.t. for 1 h. The compound was purified over preparative HPLC eluted with MeCN / H_2O (0.1% TFA) (10% MeCN for 10 min., then 10 - 90% MeCN over 40 min., followed by 90% MeCN for 5 min.) to give 195 µg (252 nmol) of the desired product as a yellow solid in 79% yield after lyophilization.

HRMS (ESI) calc. for $C_{33}H_{25}ClN_6O_{11}S_2$ $[M+3H]^+$: 781.0784; found 781.0772.

1.5.11 4-((4-(((2-amino-6-chloropyrimidin-4-yl)oxy)methyl)benzyl)carbamoyl)-2-(6-hydroxy-3-oxo-3H-xanthen-9-yl)benzoate (CP-Fluorescein)

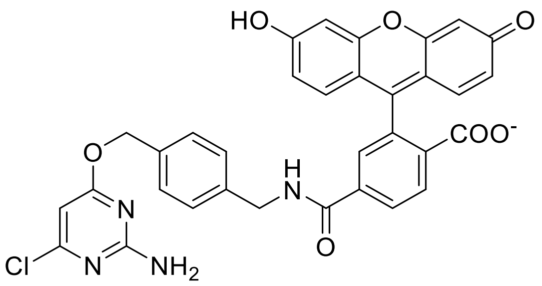

Fluorescein-6-COOH (25.0 mg, 66.4 µmol, 1.0 equiv.) was dissolved in dry DMSO (1.25 mL) and DIPEA (22.0 µL, 133 µmol, 2.0 equiv.) as well as TSTU (24.0 mg, 79.7 µmol, 1.2 equiv.) were added and the mixture was stirred at r.t. for 30 min. Afterwards, CP-NH_2 (26.4 mg, 99.7 µmol, 1.5 equiv.) was added and the reaction mixture was stirred at r.t. for 1 h. The resulted mixture was quenched with acetic acid (22.0 µL) and 10% H_2O, then the compound was purified over preparative HPLC eluted with MeCN / H_2O (0.1% TFA) (10% MeCN for 10 min., then 10 - 90% MeCN over 40 min., followed by 90% MeCN for 5 min.) to give 31 mg (49.8 µmol) of the desired product in 75% yield after lyophilization.

HRMS (ESI) calc. for $C_{33}H_{24}ClN_4O_7^+$ $[M+H]^+$: 623.1328; found 623.1327.

1.5.12 4-((4-(((2-amino-6-chloropyrimidin-4-yl)oxy)methyl)benzyl)carbamoyl)-2-(6-(dimethylamino)-3-(dimethyliminio)-10,10-dimethyl-3,10-dihydroanthracen-9-yl)benzoate (CP-CPY)

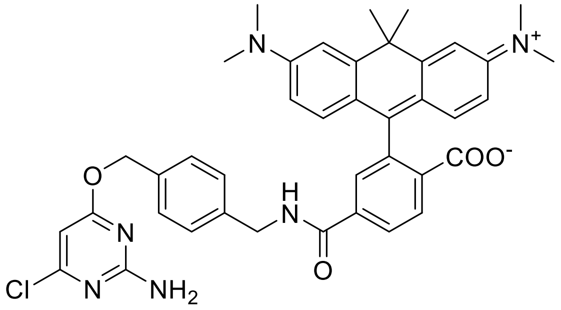

CPY-6-COOH[1] (250 mg, 530 µmol, 1.0 equiv.) was dissolved in dry DMSO (2 mL) and DIPEA (362 µL, 2.19 mmol, 4.0 equiv.) as well as TSTU (231 mg, 767 µmol, 1.4 equiv.) were added and the mixture was stirred at r.t. for 5 min. Afterwards, CP-NH_2 (217 mg, 821 µmol, 1.5 equiv.) was added and the reaction mixture was stirred at r.t. for 35 min. The resulted mixture was acidified with acetic acid (362 µL) and H_2O (500 µL), then the compound was purified over preparative HPLC eluted with MeCN / H_2O (0.1% TFA) (10% MeCN for 10 min., then 10 - 90% MeCN over 40 min., followed by 90% MeCN for 5 min.) to give 130 mg (184.9 µmol) of the desired product in 34% yield after lyophilization.

^{1}H NMR (400 MHz, acetone-*d*$_6$) *δ* [ppm] = 8.51 (t, J = 6.4 Hz, 1H), 8.23 (d, J = 8.1 Hz, 1H), 8.12 (d, J = 8.7 Hz, 1H), 7.67 (s, 1H), 7.39 – 7.30 (m, 4H), 7.11 (s, 2H), 6.67 (s, 4H), 6.36 (s, 1H), 6.07 (m, J = 10.7, 2.5 Hz, 1H), 5.30 (m, J = 11.2, 2.5 Hz, 2H), 4.55 (d, J = 5.9 Hz, 2H), 3.11 (s, 12H), 1.89 (d, J = 2.5 Hz, 3H), 1.76 (d, J = 2.4 Hz, 3H).

^{13}C NMR (101 MHz, acetone-*d*$_6$) *δ* [ppm] = 171.72, 165.87, 161.60, 140.12, 136.37, 134.01, 129.34, 129.25, 128.85, 120.23, 113.03, 110.69, 96.16, 68.31, 44.02, 40.62, 35.59, 33.04, 30.42, 30.22, 30.03, 29.84, 29.65, 29.45, 29.26, 26.13.

HRMS (ESI) calc. for $C_{40}H_{39}ClN_6O_4^+$ $[M+H]^+$: 703.2794; found 703.2792.

1.5.13 4-((4-(((2-amino-6-chloropyrimidin-4-yl)oxy)methyl)benzyl)carbamoyl)-2-(7-(dimethylamino)-3-(dimethyliminio)-5,5-dimethyl-3,5-dihydrodibenzo[b,e]silin-10-yl)benzoate (CP-SiR)

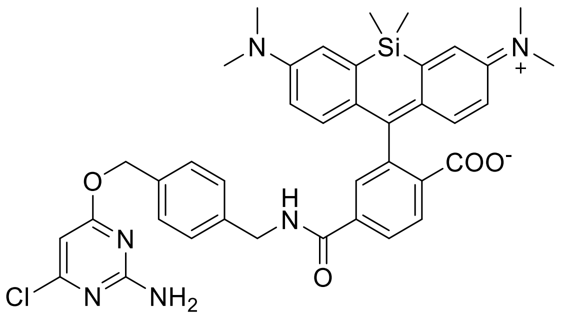

SiR-6-COOH[4] (481 mg, 1.02 mmol, 1.1 equiv.) was dissolved in dry DMSO (4 mL) and DIPEA (919 µL, 5.56 mmol, 6.0 equiv.) was added. The mixture was sonicated until complete solution and TSTU (391 mg, 1.30 mmol, 1.4 equiv.) were added and the mixture was stirred at r.t. for 5 min. Afterwards, CP-NH_2 (294 mg, 1.11 mmol, 1.2 equiv.) was added and the reaction mixture was stirred at r.t. for 2h. The resulted mixture was quenched by addition of acetic acid (973 µL) and 10% H_2O, followed by purification over preparative HPLC eluted with MeCN / H_2O (0.1% FA) (10% - 90% MeCN over 60 min) to give. 355 mg (494 µmol) of the desired product in 53% yield after lyophilization.

HRMS (ESI): calc. for $C_{39}H_{39}N_6O_4Si^+$ $[M+H]^+$: 719.2563; found 719.2561.

1.6 CLIP substrates

1.6.1 4-((4-(((4-aminopyrimidin-2-yl)oxy)methyl)benzyl)carbamoyl)-2-(6-(dimethylamino)-3-(dimethyliminio)-10,10-dimethyl-3,10-dihydroanthracen-9-yl)benzoate (BC-CPY)

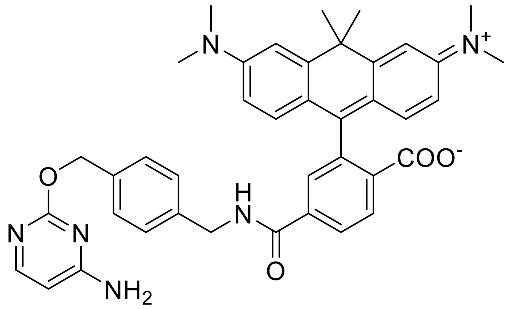

CPY-6-COOH[1] (250 mg, 530 µmol, 1.0 equiv.) was dissolved in dry DMSO (2 mL). DIPEA (362 µL, 2.19 mmol, 4.0 equiv.) and TSTU (231 mg, 767 µmol, 1.4 equiv.) were added and the mixture was stirred at r.t. for 5 min. Afterwards, BC-NH_2 (189 mg, 821 µmol, 1.5 equiv.) was added and the reaction mixture was stirred at r.t. for 35 min. The resulted mixture was acidified with acetic acid (362 µL) and H_2O (500 µL), then compound was purified over preparative HPLC eluted with MeCN / H_2O (0.1% TFA) (10% MeCN for 10 min., then 10 - 90% MeCN over 40 min., followed by 90% MeCN for 5 min.) to give 180 mg (269.1 µmol) of the desired product in 49% yield after lyophilization.

^{1}H NMR (400 MHz, acetone-d_6) δ [ppm] = 8.51 (t, J = 6.9 Hz, 1H), 8.21 (d, J = 8.1 Hz, 1H), 8.10 – 8.01 (m, 2H), 7.61 (s, 1H), 7.38 (d, J = 7.4 Hz, 2H), 7.32 (d, J = 7.8 Hz, 2H), 7.27 (s, 1H), 7.05 (s, 2H), 6.61 (s, 4H), 6.40 (d, J = 6.6, 2.4 Hz, 1H), 5.36 (s, 2H), 4.56 – 4.50 (m, 2H), 3.04 (s, 12H), 1.88 (d, J = 2.5 Hz, 3H), 1.76 (d, J = 2.6 Hz, 3H).

^{13}C NMR (101 MHz, acetone-d_6) δ [ppm] = 169.57, 165.97, 162.80, 152.54, 152.01, 148.78, 141.22, 140.20, 136.01, 130.47, 129.26, 128.83, 126.23, 124.12, 120.13, 112.83, 110.38, 100.42, 69.65, 44.02, 43.89, 40.54, 39.65, 35.58, 33.19, 30.42, 30.23, 30.03, 29.84, 29.65, 29.46, 29.26.

HRMS (ESI): calc. for $C_{40}H_{42}N_6O_4^{2+}$ $[M+2H]^{2+}$: 335.1628; found 335.1629.

1.7 Additional substrates

1.7.1 2-(6-(dimethylamino)-3-(dimethyliminio)-3H-xanthen-9-yl)-4-(methylcarbamoyl)benzoate (meAm-6-TMR)

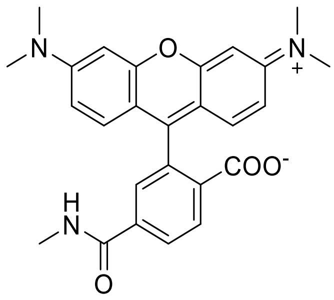

To a solution of TMR-6-COOH (1.0 mg, 2.32 µmol, 1.1 equiv.) in dry DMSO (500 µL), TSTU (763 µg, 2.53 µmol, 1.2 equiv.) was added and the mixture was stirred at r.t. for 5 min. Afterwards, DIPEA (1.4 µL, 8.45 µmol, 4 equiv.) and methylamine (2 M, 1.06 µL, 2.11 µmol, 1 equiv.) were added and the reaction mixture was stirred at r.t. overnight. The compound was purified over preparative HPLC eluted with MeCN / H_2O (0.1% TFA) (10% MeCN for 10 min., then 10 - 90% MeCN over 40 min., followed by 90% MeCN for 5 min.) to give 91.1 µg (205.4 nmol) of the desired product in 10% yield after lyophilization.

^{1}H NMR (TMR-6-COOH) (400 MHz, DMSO-d_6) δ [ppm] = 8.21 (dd, J = 8.0, 1.4 Hz, 1H), 8.17 – 7.99 (m, 1H), 7.61 – 7.56 (m, 1H), 6.58 – 6.45 (m, 6H), 2.95 (s, 12H).

^{13}C NMR (TMR-6-COOH) (101 MHz, DMSO-d_6) δ [ppm] = 168.56, 166.53, 152.67, 152.47, 131.16, 128.91, 109.56, 105.91, 98.43, 40.46, 40.26.

HRMS (ESI): calc. for $C_{26}H_{26}N_3O_4^+$ $[M+H]^+$: 444.1918; found 444.1914.

1.7.2 2-(6-(dimethylamino)-3-(dimethyliminio)-10,10-dimethyl-3,10-dihydroanthracen-9-yl)-4-(methylcarbamoyl)benzoate (meAm-6-CPY)

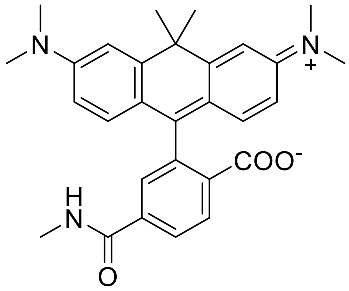

To a solution of CPY-6-COOH (1.0 mg, 2.19 µmol, 1.1 equiv.) in dry DMSO (500 µL), TSTU (719 µg, 2.39 µmol, 1.2 equiv.) was added and the mixture was stirred at r.t. for 5 min. Afterwards, DIPEA (1.32 µL, 7.97 µmol, 4.0 equiv.) and methylamine (2 M, 0.996 µL, 1.99 µmol, 1.0 equiv.) were added and the reaction mixture was stirred at r.t. overnight. The compound was purified over preparative HPLC eluted with MeCN / H_2O (0.1% TFA) (10% MeCN for 10 min., then 10 - 90% MeCN over 40 min., followed by 90% MeCN for 5 min.) to give 97.7 µg (208.1 nmol) of the desired product in 10% yield after lyophilization.

HRMS (ESI): calc. for $C_{29}H_{32}N_3O_3$ $[M+H]^+$: 470.2438; found 470.2434.

1.7.3 2-(6-(dimethylamino)-3-(dimethyliminio)-3H-xanthen-9-yl)-5-(methylcarbamoyl)benzoate (meAm-5-TMR)

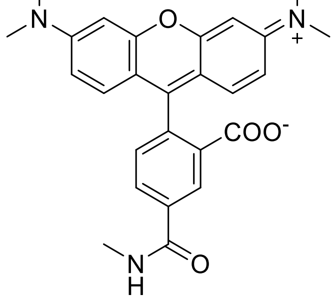

To a solution of TMR-5-COOH (2.5 mg, 5.81 μmol, 1.0 equiv.) in dry DMSO (500 μL), BOP (2.59 mg, 8.71 μmol, 1.5 equiv.) was added and the reaction was shaken at r.t. and 500 rpm for 5 min. DIPEA (3.84 μL, 23.2 μmol, 4.0 equiv.) and methylamine (2M in THF, 4.36 μL, 8.71 μmol, 1.5 equiv.) were added and the reaction was shaken at r.t. and 500 rpm for 4 h. The crude product was acidified with acetic acid and purified over preparative HPLC eluted with MeCN / H_2O (0.1% FA) (10% - 90% MeCN over 50 min) to give 0.97 mg (2.19 μmol) of the desired product in 38% yield after lyophilization.

^{1}H NMR (TMR-5-COOH) (400 MHz, DMSO-*d*$_6$) *δ* [ppm] = 8.39 (s, *J* = 1.5 Hz, 1H), 8.28 (dd, *J* = 8.1, 1.5 Hz, 1H), 7.33 (d, *J* = 8.0 Hz, 1H), 6.58 – 6.45 (m, 6H), 2.95 (s, 12H).

^{13}C NMR (TMR-5-COOH) (101 MHz, DMSO-*d*$_6$) *δ* [ppm] = 168.31, 166.09, 152.03, 135.96, 132.76, 128.50, 109.05, 97.95, 40.15, 39.99, 39.79.

HRMS (ESI): calc. for $C_{26}H_{26}N_3O_4^+$ $[M+H]^+$: 444.1923; found 444.1914.

1.7.4 2-(6-(dimethylamino)-3-(dimethyliminio)-10,10-dimethyl-3,10-dihydroanthracen-9-yl)-5-(methylcarbamoyl)benzoate (meAm-5-CPY)

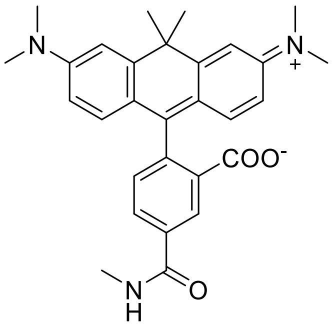

To a solution of CPY-5-COOH (2.5 mg, 5.48 μmol, 1.0 equiv.) in dry DMSO (1 mL), BOP (0.5 M in DMSO, 17.4 μL, 8.71 μmol, 1.5 equiv.) was added and the reaction was shaken at r.t and 500 rpm for 5 min. DIPEA (3.62 μL, 21.9 μmol, 4.0 equiv.) and methylamine (2 M in THF, 4.11 μL, 8.21 μmol, 1.5 equiv.) were added and the reaction was shaken at 500 rpm, r.t. for 4 h. The crude product was acidified with acetic acid and purified over preparative HPLC eluted with MeCN / H_2O (0.1% FA) (10% - 90% MeCN over 50 min) to give 0.77 mg (1.64 μmol) of the desired product in 30% yield after lyophilization.

HRMS (ESI): calc. for $C_{29}H_{32}N_3O_3^+$ $[M+H]^+$: 470.2443; found 470.2437.

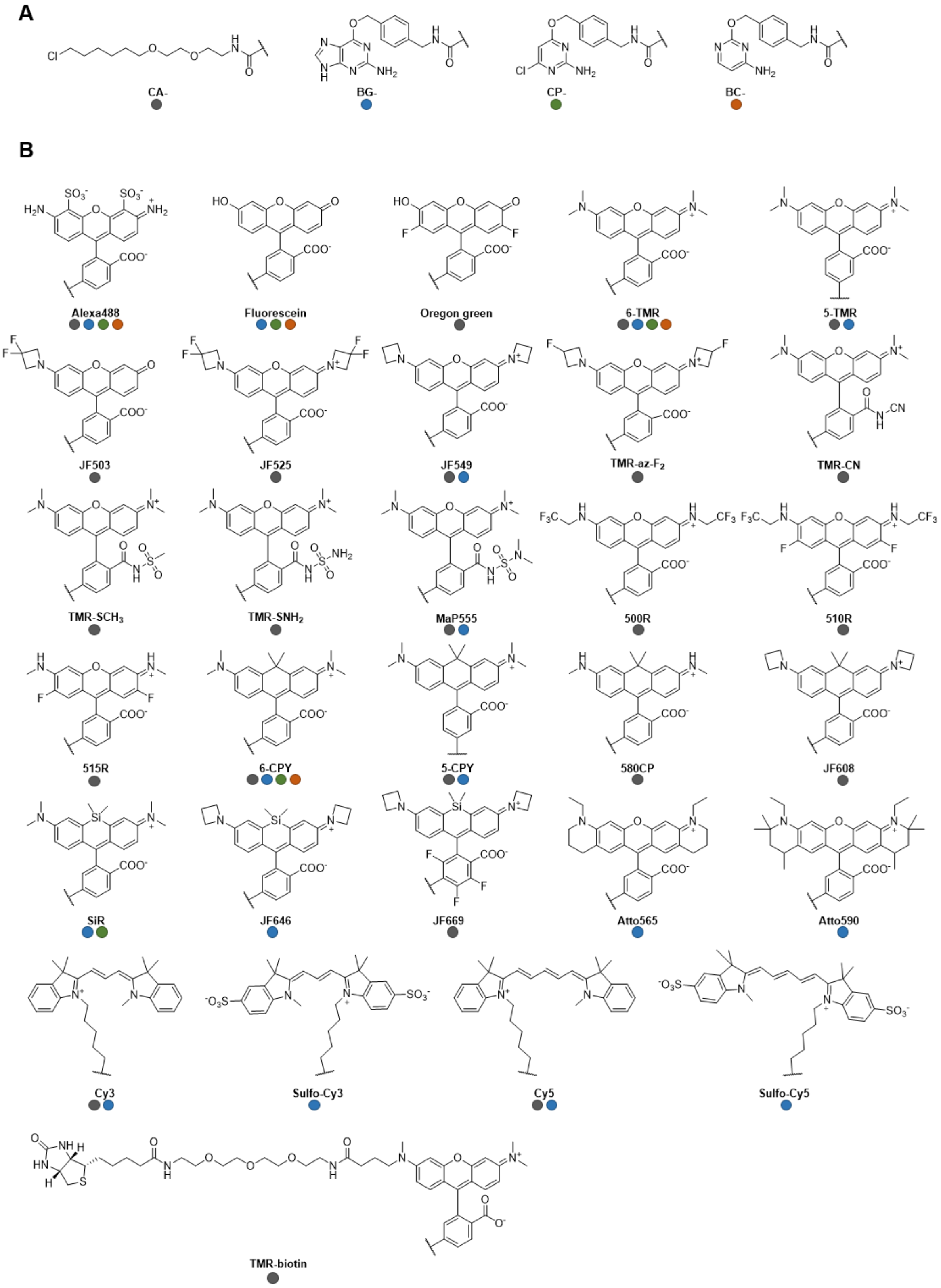

Figure S1: Chemical structures of SLP substrates. (continued on the next page)

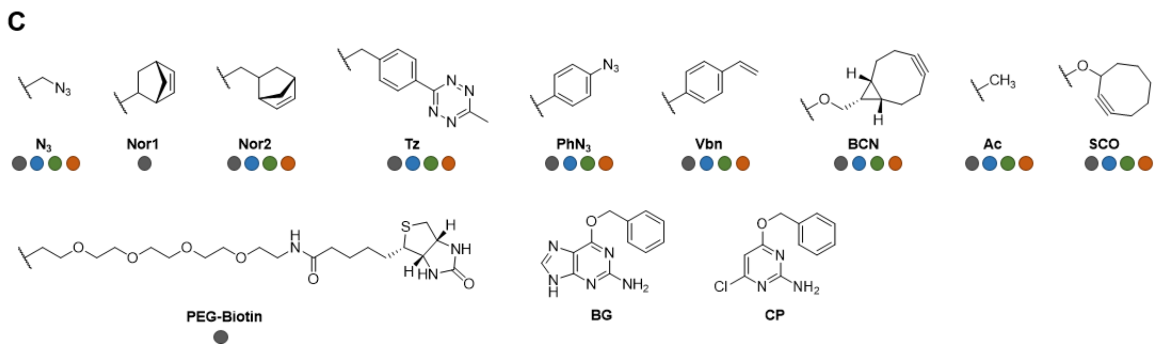

Figure S1 (continued): Chemical structures of SLP substrates.

A. Chemical structures of HT7 (CA), SNAP (BG and CP) and CLIP (BC) core substrates. **B**. Chemical structures of fluorescent substituents. **C**. Chemical structures of non-fluorescent substituents. Colored dots indicate the tested substrates for the corresponding SLPs (grey = CA, blue = BG, green = CP and orange = BC)**.**

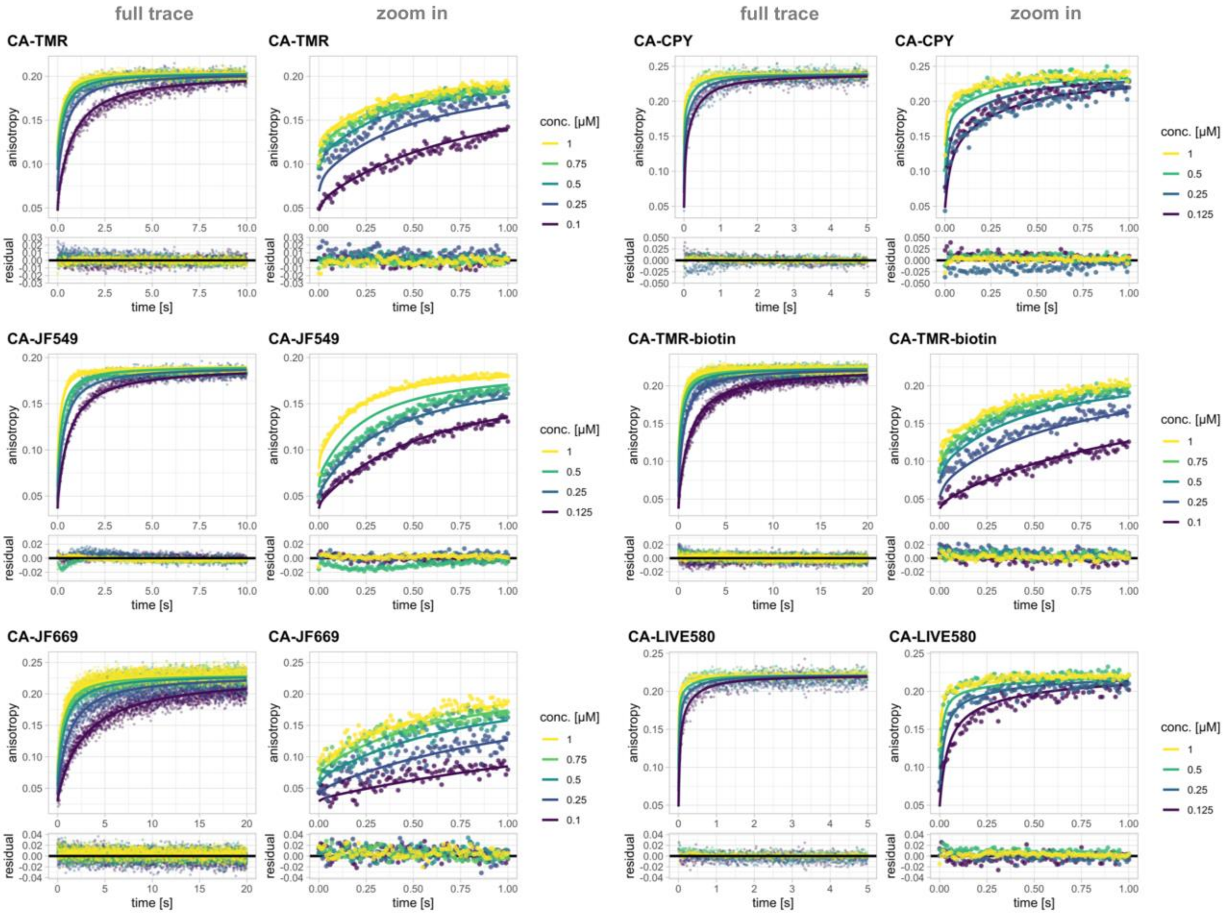

Figure S2: Labeling kinetics of HT7 with fluorescent CA substrates.

Full anisotropy traces (points) and predications of fits based on model 2 (lines) along with zoom on the first second are represented on the top panels. Residuals from the fits are depicted in the bottom panels. Kinetics were recorded by following fluorescence anisotropy changes over time using a stopped flow device. All conditions are 1:1 mixtures of protein and substrate at the given concentrations (conc.). For structures of CA substrates see **Fig. S1.**

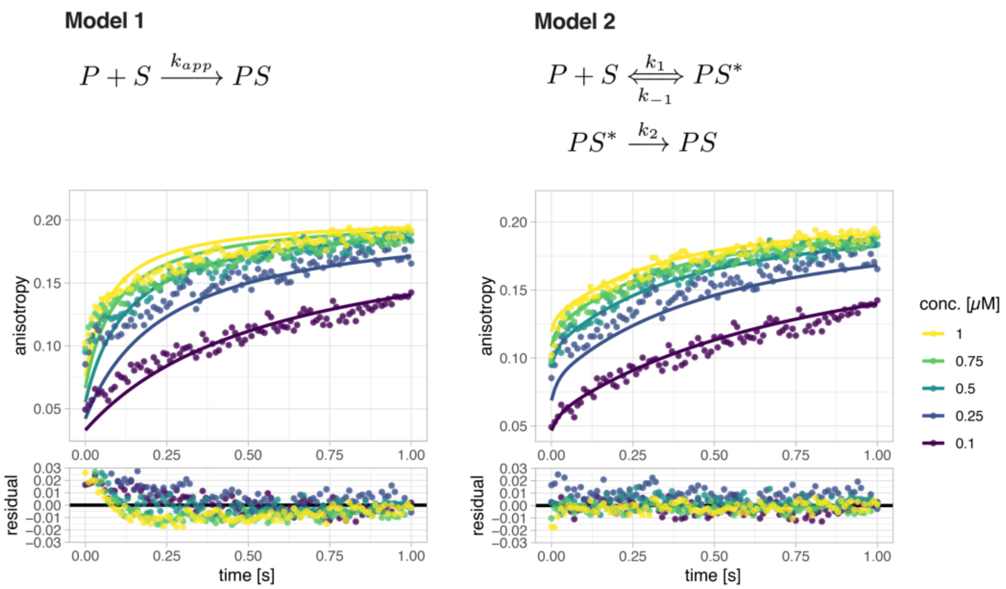

Figure S3: Comparison of model 1 and model 2 fitted to HT7 labeling kinetics.

Anisotropy traces (points) and predications of fits based on either model 1 or model 2 (lines) of the labeling reaction between HT7 and CA-TMR are represented in the top panels. Residuals from the fits are depicted in the bottom panels. Kinetics were recorded by following fluorescence anisotropy changes over time using a stopped flow device. All conditions are 1:1 mixtures of protein and substrate at the given concentrations (conc.). Model 2 describes the data better than the simplified model 1. For structures of CA substrates see **Fig. S1.**

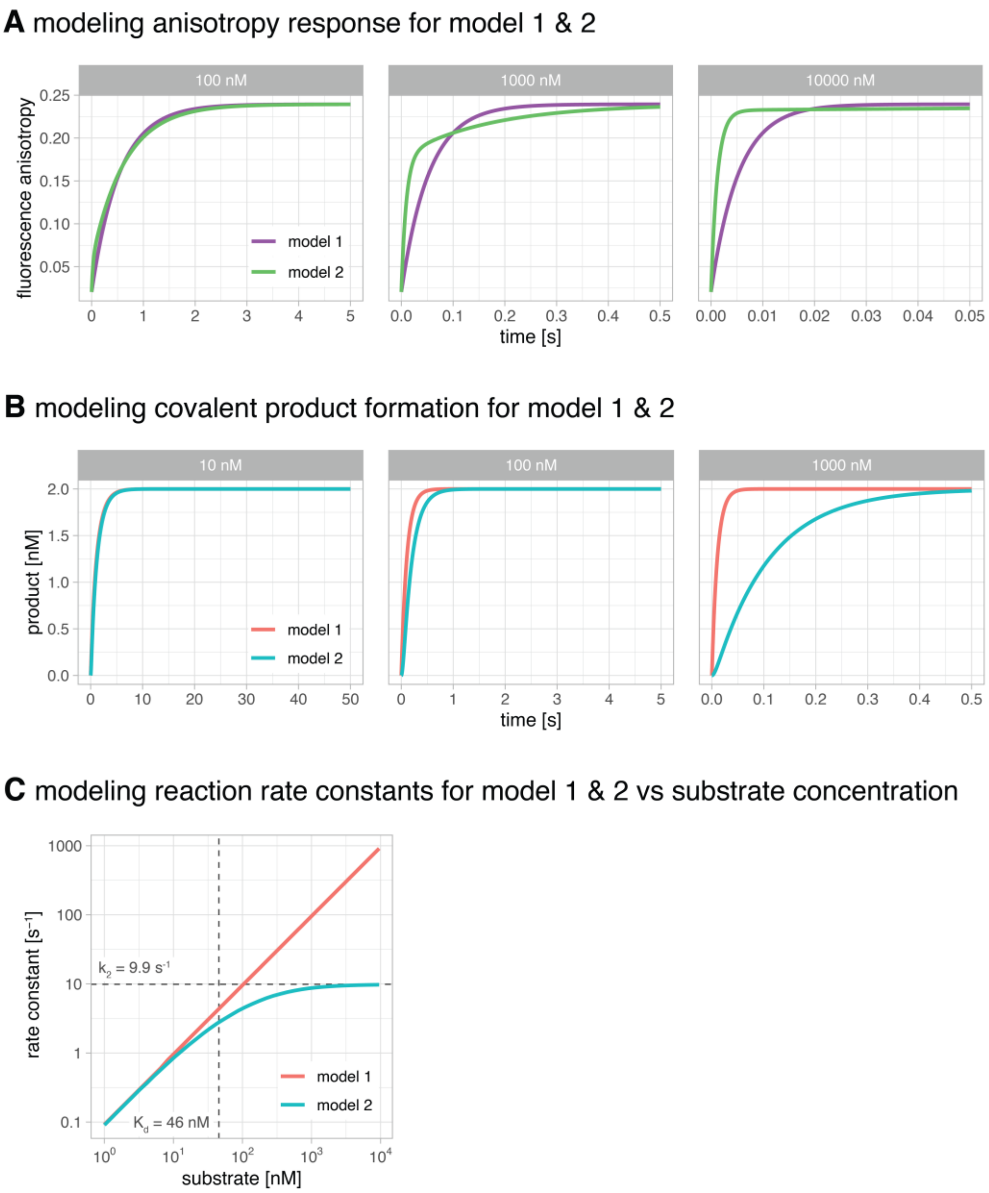

Figure S4: Modeling of HT7 labeling kinetics using measured parameters to compare the kinetic models 1 and 2.

A. Modeling of the fluorescence anisotropy response at different reactant concentrations using model 1 and 2 with parameters determined for HT7 labeling with CA-TMR. At concentrations below K_d (327 nM for CA-TMR) both models yield a rather similar response. At concentrations higher than K_d (1000 nM) the response for model 2 shows a strong biphasic character as observed in the measured data, which is not matching the monoexponential behavior of model 1. At very high concentrations (10000 nM) the response for model 2 is again close to a monoexponential curve but the kinetic is much faster than the model 1 curve. This happens since the rise in fluorescence anisotropy for model 2 in the first milliseconds is not due to covalent reaction but mostly binding (k_1). The binding rate constant k_1 is faster than k_{app} if k_{-1} is not zero ($k_{app} = k_1 * k_2 / (k_2 + k_{-1})$). Hence directly estimating k_{app} from fluorescence anisotropy traces by fitting model 1 to the data is only valid for concentrations below K_d or if $k_{-1} << k_2$. **B.** Modeling the formation of covalently labeled product at different reactant concentrations using model 1 and 2 with parameters determined for HT7 labeling with CA-CPY. At concentrations below K_d (46 nM for CA-CPY) both models yield a rather similar behavior. At higher concentrations model 1 predicts a much faster product formation than model 2 since it does not account for enzyme saturation. **C.** Plot of the apparent first order reaction rate constant for product formation against substrate concentration for model 1 and 2 with parameters for CA-CPY. In contrast to model 1, model 2 accounts for enzyme saturation leading to a maximum reaction rate of $k_{max} = k_2 = 9.9\ s^{-1}$. The models start do diverge significantly once the substrate concentration exceeds K_d (46 nM). As a consequence, model 2 should be used for predicting formation of labeled HT7 if labeling is performed at high concentrations.

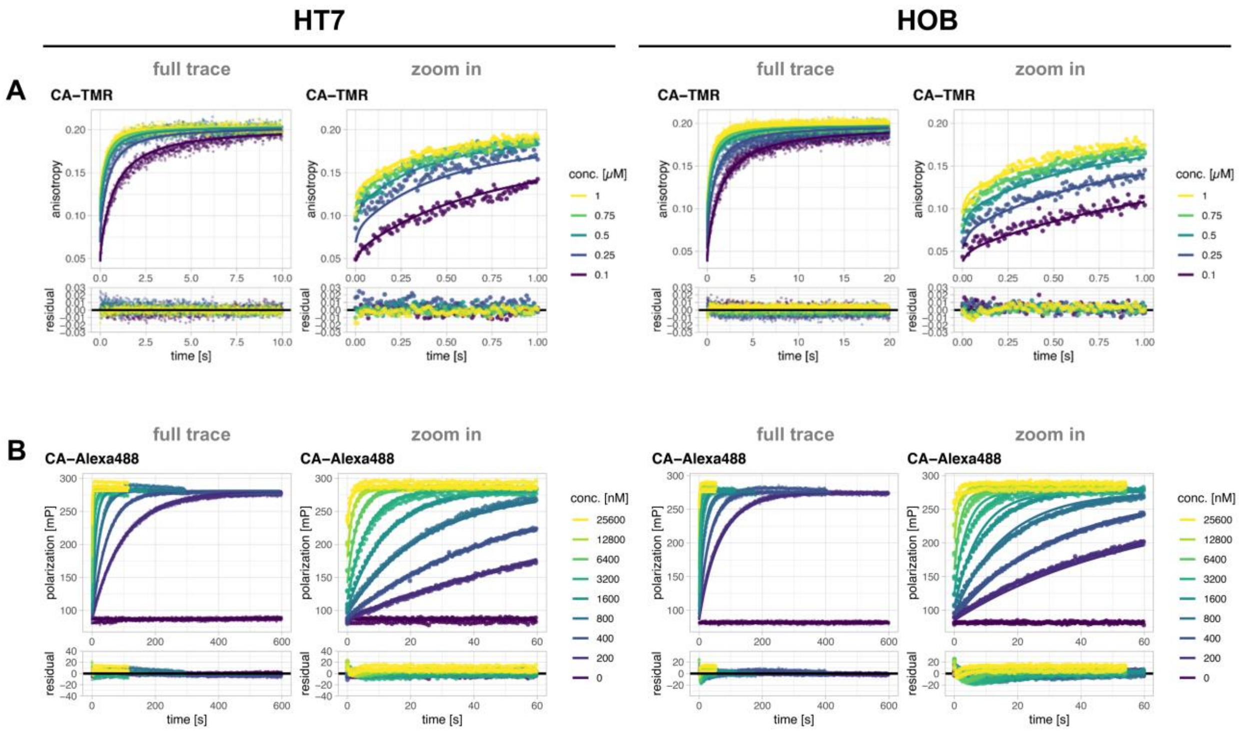

Figure S5: Labeling kinetics of HT7 and HOB with CA-TMR (**A**) and CA-Alexa488 (**B**).

A: Labeling kinetics of HT7 and HOB with CA-TMR. Full anisotropy traces (points) and predications of fits based on model 2 (lines) along with zoom on the initial part are represented on the top panels. Residuals from the fits are depicted in the bottom panels. Kinetics were recorded by following fluorescence anisotropy changes over time using a stopped flow device. All conditions are 1:1 mixtures of protein and substrate at the given concentrations (conc.). **B:** Labeling kinetics of HT7 and HOB with CA-Alexa488. Full fluorescence polarization traces (points) and predications of fits based on model 1 (lines) along with zoom on the initial part are represented on the top panels. Residuals from the fits are depicted in the bottom panels. Kinetics were recorded by following fluorescence polarization changes over time using a plate reader. All experiments were performed at a fixed substrate concentration of 50 nM with varying protein concentrations. For structures of CA substrates see **Fig. S1.**

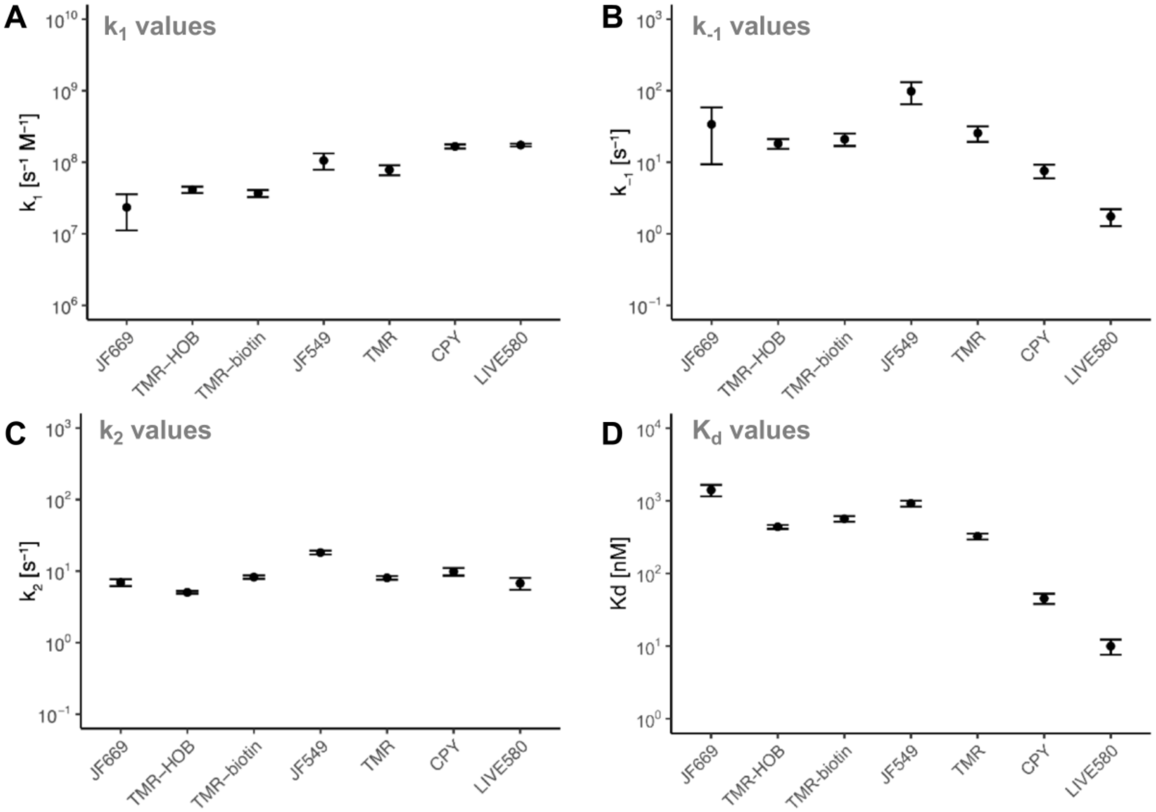

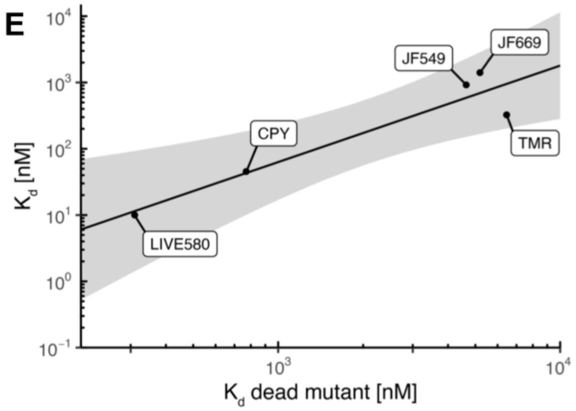

F

Substrate	K_d (95% CI) [μM] calc. from kinetic	K_d (95% CI) [μM] from HT^{D106A} titration
**CA-TMR**	0.33 (0.30 to 0.36)	6.24 (5.25 to 7.41)
**CA-JF549**	0.92 (0.84 to 1.01)	4.48 (3.61 to 5.55)
**CA-JF669**	1.41 (1.16 to 1.66)	5.16 (4.59 to 5.79)
**CA-CPY**	0.045 (0.038 to 0.053)	0.74 (0.65 to 0.85)
**CA-LIVE580**	0.010 (0.008 to 0.012)	0.31 (0.27 to 0.35)

Figure S6: Rate and equilibrium constants of HT7 labeling with various fluorescent CA substrates.

Rate constants k_1 (**A**), k_{-1} (**B**), k_2 (**C**) and the calculated dissociation constants ($K_d = k_{-1}/k_1$, **D.**) obtained from fitting model 2 to stopped flow labeling experiments of HT7 and HOB. The catalytic rate constant (k_2) is rather constant among these substrates, while there are significant differences in the dissociation constant (K_d). The K_d variations are due to large differences in k_{-1} and minor differences in k_1. As a result, differences in k_{app} can be mostly explained by affinity differences of HT7 towards its substrates. **E.** Correlation between the calculated K_d from the stopped flow kinetic experiments and the K_d obtained from titration experiments performed with the dead mutant $HT7^{D106A}$. Log transformed values were fitted to a linear model ($\log(y) = 1.455 * \log(x) - 2.567$; black line, 95% confidence bands in grey, depicting the area in which the true regression line lies with 95% confidence). The linear correlation in logarithmic space suggests that the K_d of CA rhodamine substrates with $HT7^{D106A}$ could represent a valid proxy to estimate their K_d with the native HT7. **F** K_d values of the tested substrates calculated from the kinetics (k_1/k_{-1}) and measured by fluorescence polarization titration against the dead mutant HT^{D106A}.

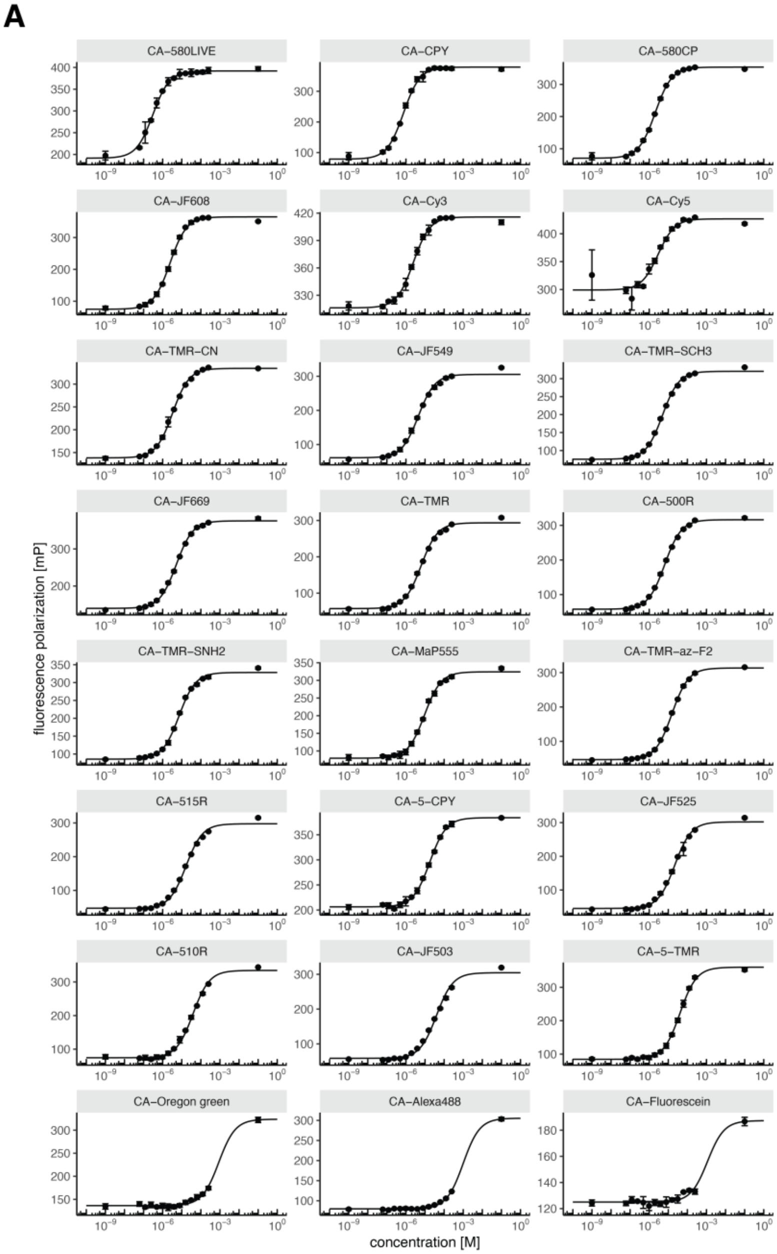

B

substrate	K_d [μM] (95%CI)
CA−580LIVE	0.31 (0.27 to 0.35)
CA−CPY	0.74 (0.65 to 0.85)
CA−580CP	2.08 (1.94 to 2.23)
CA−JF608	2.43 (2.16 to 2.73)
CA−Cy3	2.44 (2.08 to 2.87)
CA−Cy5	2.92 (1.70 to 5.08)
CA−TMR−CN	3.29 (2.98 to 3.62)
CA−JF549	4.48 (3.61 to 5.55)
CA−TMR−SCH3	4.94 (4.36 to 5.61)
CA−JF669	5.16 (4.59 to 5.79)
CA−TMR	6.24 (5.25 to 7.41)
CA−500R	6.51 (5.92 to 7.16)
CA−TMR−SNH2	7.23 (6.20 to 8.43)
CA−MaP555	9.36 (8.06 to 10.9)
CA−TMR−az−F2	15.5 (14.8 to 16.3)
CA−515R	16.7 (13.5 to 20.8)
CA−5−CPY	17.6 (15.9 to 19.6)
CA−JF525	21.8 (17.9 to 26.5)
CA−510R	38.4 (32.1 to 45.9)
CA−JF503	39.7 (30.1 to 52.0)
CA−5−TMR	40.9 (36.9 to 45.4)
CA−Oregon green	866 (697 to 1109)
CA−Alexa488	992 (894 to 1109)
CA−Fluorescein	1135 (736 to 2098)

Figure S7: Affinity of the dead mutant HT7^{D106A} to fluorescent CA substrates**.**

A. Titration curves of fluorescent CA substrates against HT7^{D106A} measured via fluorescence polarization. The FP value of each dye fully bound to native HT7 was added at c = 0.1 M to improve fitting of the upper plateau. (See corresponding methods section for more details). **B.** Table summarizing fitted K_d values with 95% confidence intervals. For structures of CA substrates see **Fig. S1.**

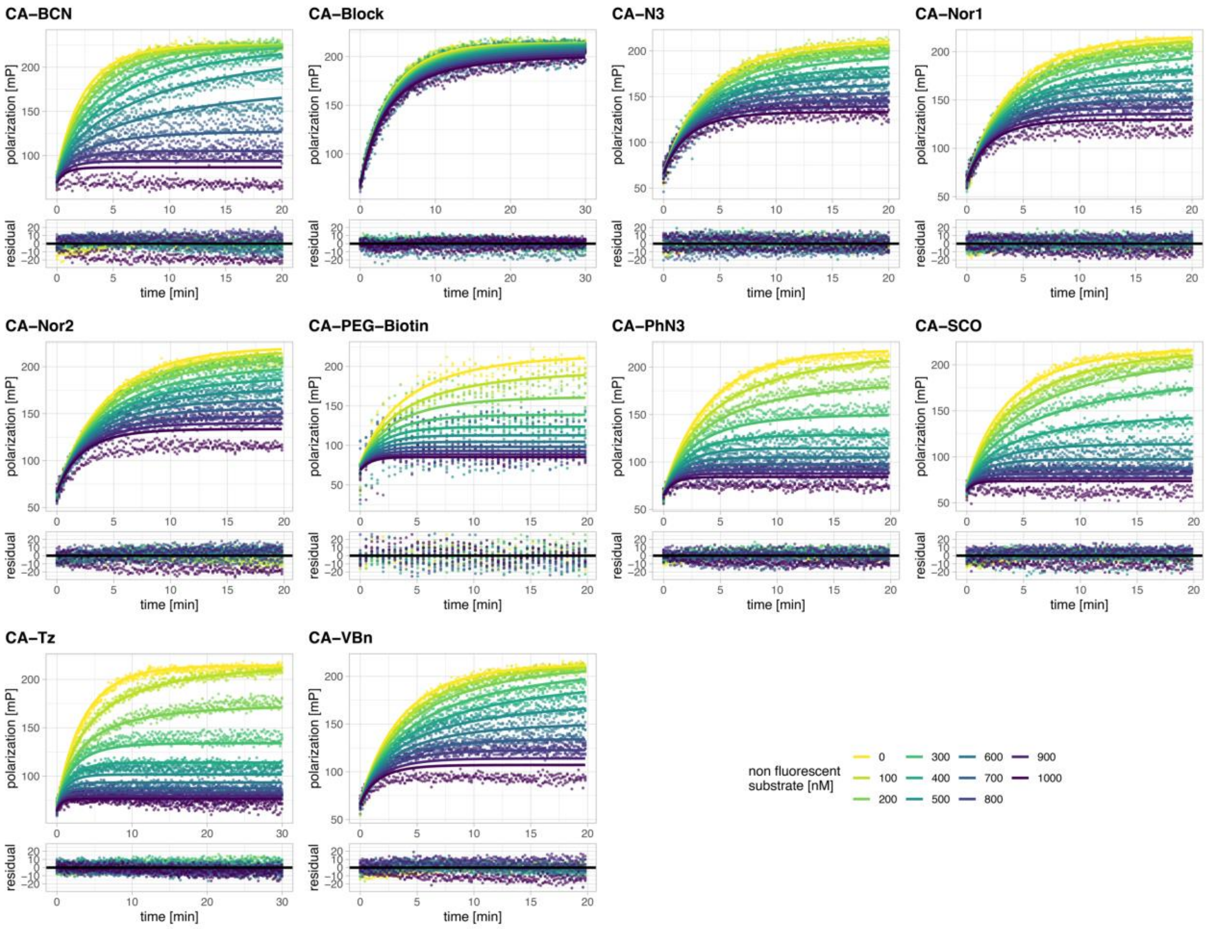

Figure S8: Labeling kinetics of HT7 with non-fluorescent CA substrates.

Fluorescence polarization traces (points) of kinetic competition assays and predications of fits (lines) based on a simple competitive model (see methods section for details) of HT7 labeling with CA-Alexa488 in the presence of different concentrations of non-fluorescent CA substrates are represented on the top panels. Residuals from the fits are depicted in the bottom panels. Kinetics were recorded by following fluorescence polarization changes over time using a plate reader. For structures of CA substrates see **Fig. S1.**

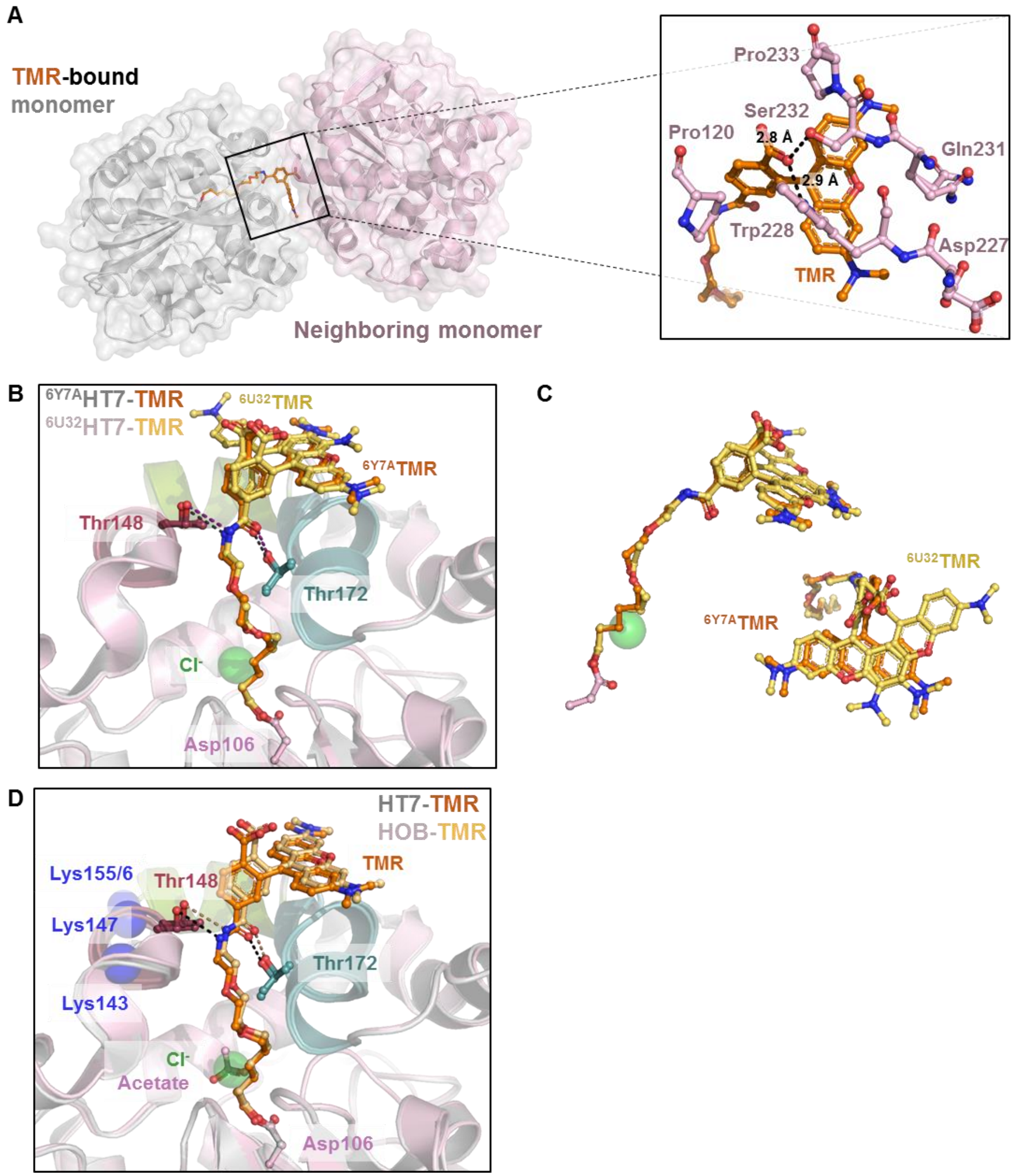

Figure S9: Additional structural information on HaloTag proteins.

A. Alkane-TMR constraints by the crystal packing. Two monomers of HT7-TMR are displayed in grey and light-pink. The alkane-TMR (orange sticks) conformation is constrained by the light-pink monomer that was generated as a symmetry mate. A zoom is shown on the right panel. **B**. Structural comparison between ^{6U32}HT7-TMR (previously published [16]) and ^{6Y7A}HT7-TMR (PDB ID 6Y7A, this study). A zoom into the binding site with hydrogen bonds between ^{6Y7A}HT7-TMR and ^{6U32}HT7-TMR and their respective reacted substrates are represented as black and dark-purple lines, respectively. **C**. Zoom into isolated catalytic aspartate and alkane-TMR substrate from both ^{6Y7A}HT7-TMR and ^{6U32}HT7-TMR crystal structures. **D**. Structural comparison between HT7-TMR and HOB-TMR. Hydrogen bonds between HT7 and HOB and their respective reacted substrates are represented as black and gold dashed lines, respectively. The blue spheres represent differences between HT7 and HOB caused by the mutations.

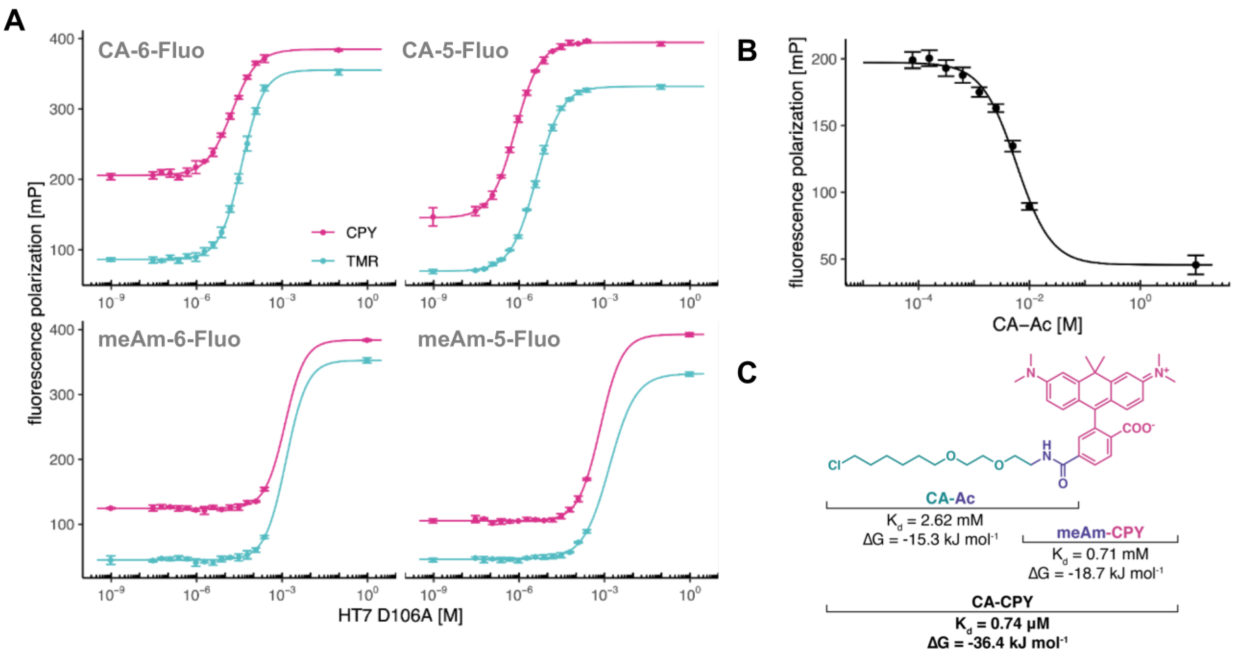

D

dye	position	ligand	K_d [µM]	95% CI [µM]
TMR	5 position	CA	39.4	(35.7 to 43.4)
		meAm	1930	(1660 to 2291)
	6 position	CA	6.24	(5.25 to 7.41)
		meAm	1512	(1402 to 1640)
CPY	5 position	CA	16.8	(15.2 to 18.6)
		meAm	1987	(1688 to 2398)
	6 position	CA	0.74	(0.65 to 0.85)
		meAm	707	(497 to 785)

Figure S10: Biochemical study of the interaction of HT7 with CA-fluorophores.

A. Affinity of the dead mutant HT7^{D106A} towards different fluorophore derivatives measured via fluorescence polarization assay. The FP value of each dye as CA substrate fully bound to native HT7 was added at c = 0.1 M or c = 1 M in order to improve fitting of the upper plateau. **B.** Affinity of HT7^{D106A} to CA-Ac measured via fluorescence polarization competition assay against CA-TMR. **C.** Summary of dissociation constants (K_d) and calculated free binding energies (ΔG) of HT7^{D106A} with CA-Ac, mAm-5-CPY and CA-5-CPY. The representation highlights the additive nature of the binding energies from the chloroalkane and the CPY moieties for the binding energy of the full substrate. **D.** Table summarizing values and confidence intervals (95%) of the fits.

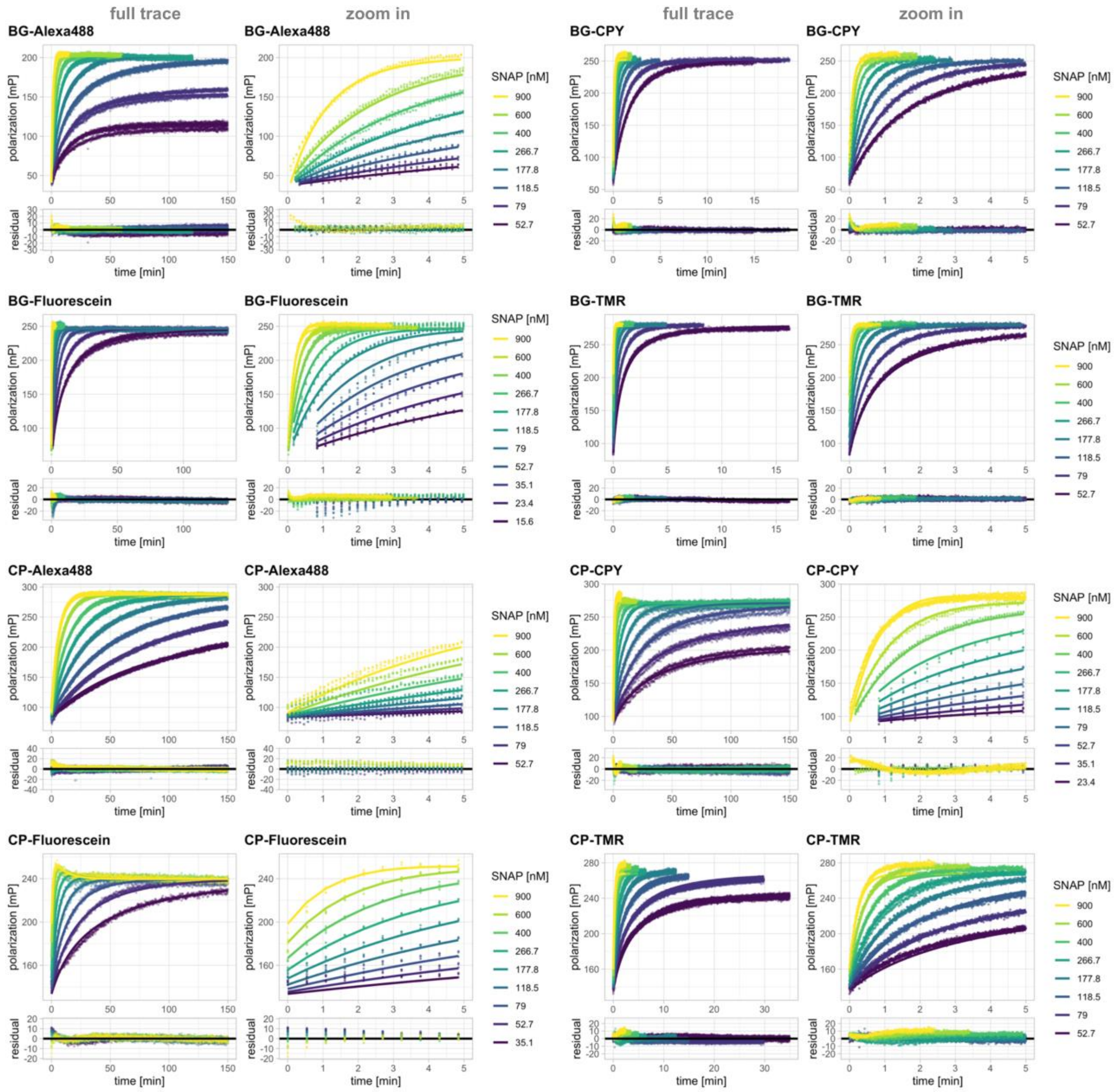

Figure S11: Labeling kinetics of SNAP with fluorescent BG and CP substrates.

Full fluorescence polarization traces (points) and predications of fits based on model 1 or 1.2 (lines) along with zoom on the initial 5 minutes are represented on the top panels. Most substrates were fitted to model 1 except CP-Fluorescein and CP-CPY, which showed an additional phase (model 1.2). Residuals from the fits are depicted in the bottom panels. Kinetics were recorded by following fluorescence polarization changes over time using a plate reader. Labeling was performed at different concentrations of SNAP protein. Substrate concentrations were aimed at 20 nM based on the dyes extinction coefficient but fitted in the model since significant deviations from the expected stoichiometry were observed. For structures of BG and CP substrates see **Fig. S1.**

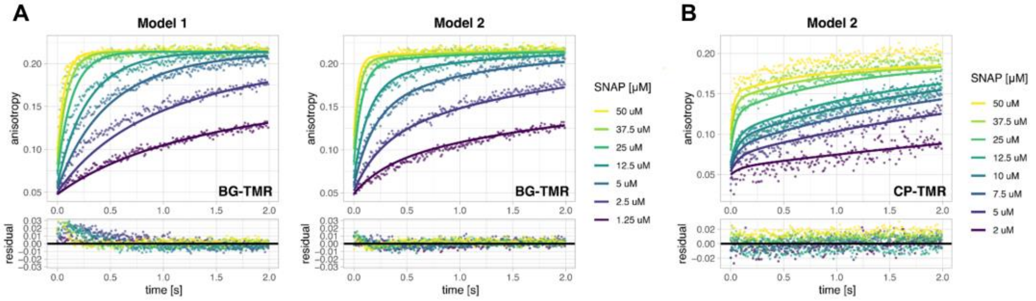

C

Substrate	K_d (95% CI) [µM]		k_{app} (± SD) [$M^{-1}s^{-1}$]	
	calc. from stopped flow kinetics	from SNAP^{C456A} titrations	calc. from stopped flow kinetics	fitted from plate reader kinetics
**BG-TMR**	2.06 (1.98 to 2.14)	0.77 (0.66 to 0.90)	2.714 (± 0.008) × 10^5	4.292 (± 0.007) × 10^5
**CP-TMR**	16.69 (15.89 to 17.49)	5.64 (5.04 to 6.30)	0.805 (± 0.004) × 10^5	0.769 (± 0.001) × 10^5

Figure S12: Labeling kinetics of SNAP measured by stopped flow fluorescence anisotropy.

A. Comparative data analysis of SNAP labeling kinetics with BG-TMR. Anisotropy traces (points) and predications of fits based on either model 1 or model 2 (lines) of the labeling reaction between SNAP and BG-TMR are represented in the top panels. Residuals from the fits are depicted in the bottom panels. Labeling was performed at different concentrations of SNAP protein and a constant substrate concentration of 1 µM. Model 2 describes the data better than the simplified model 1. (for model description see **Fig. 1**). **B.** Kinetic traces of SNAP labeling with CP-TMR represented as previously explained and fit with model 2. For structures of BG and CP substrates see **Fig. S1. C.** K_d and k_{app} values calculated from parameters obtained by fitting model 2 to stopped flow anisotropy data ($K_d = k_{-1}/k_1$, $k_{app} = k_1*k_2/(k_{-1}+k_2)$) compared to values directly fitted to fluorescence polarization assay with SNAP^{C145A} (K_d) and plate reader kinetics at lower SNAP concentrations fitted with model 1 (k_{app}).

A

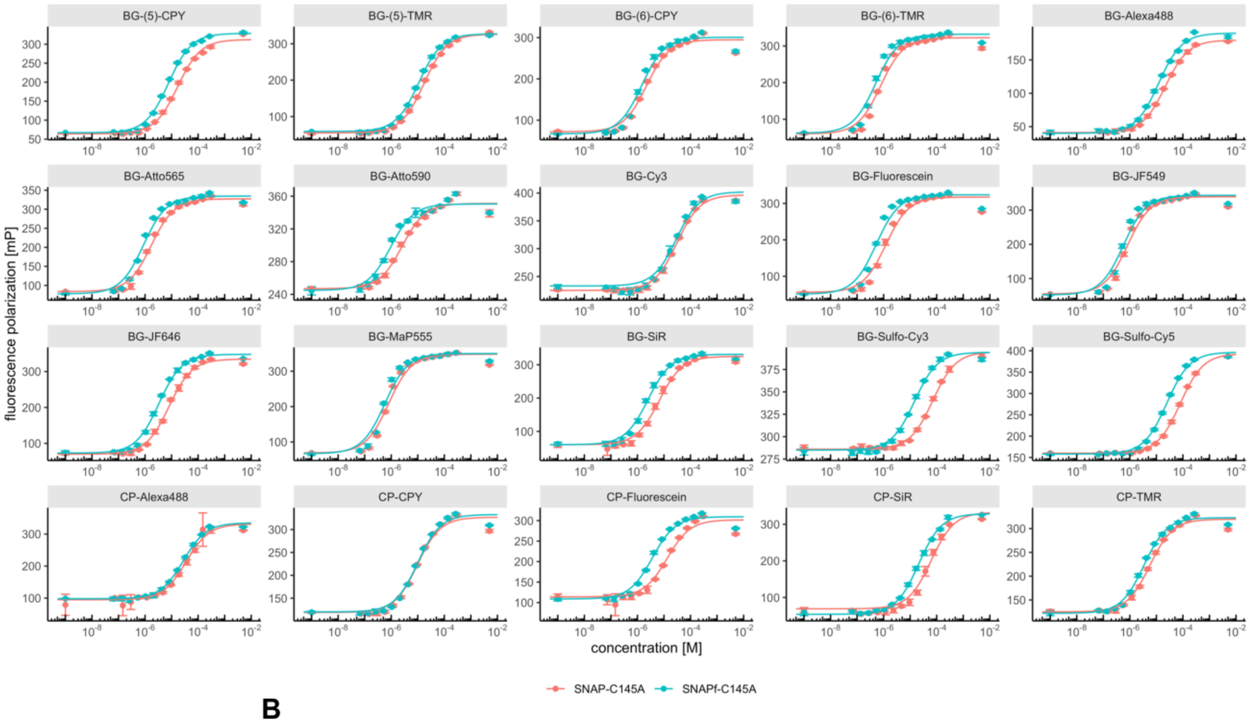

B

Substrate	K_d SNAP^{C145A} (95% CI) [μM]	K_d SNAPf^{C145A} (95% CI) [μM]	ΔK_d
BG-(5)-CPY	17.0 (15.7 - 18.3)	7.71 (7.24 - 8.21)	2.20
BG-(5)-TMR	17.2 (16.5 - 17.9)	10.9 (10.4 - 11.6)	1.58
BG-(6)-CPY	2.20 (1.90 - 2.54)	1.44 (1.23 - 1.69)	1.53
BG-(6)-TMR	0.77 (0.66 - 0.90)	0.50 (0.43 - 0.58)	1.54
BG-Alexa488	21.8 (20.8 - 22.8)	12.2 (11.4 - 13.1)	1.79
BG-Atto565	1.73 (1.55 - 1.94)	0.88 (0.78 - 0.99)	1.97
BG-Atto590	2.60 (2.22 - 3.05)	0.95 (0.80 - 1.12)	2.74
BG-Cy3	31.0 (28.3 - 34.0)	29.3 (24.6 - 34.8)	1.06
BG-Fluorescein	1.21 (1.04 - 1.41)	0.52 (0.44 - 0.62)	2.33
BG-JF549	0.82 (0.71 - 0.95)	0.58 (0.50 - 0.67)	1.41
BG-JF646	7.97 (7.38 - 8.59)	3.32 (3.06 - 3.61)	2.40
BG-MaP555	0.80 (0.69 - 0.92)	0.58 (0.50 - 0.67)	1.38
BG-SiR	6.64 (5.87 - 7.51)	2.52 (2.29 - 2.76)	2.63
BG-Sulfo-Cy3	71.3 (66.4 - 76.5)	14.6 (13.1 - 16.3)	4.88
BG-Sulfo-Cy5	79.5 (76.2 - 83.0)	24.8 (23.3 - 26.3)	3.21
CP-Alexa488	35.1 (26.3 - 46.4)	27.5 (25.4 - 29.8)	1.28
CP-CPY	9.61 (8.30 - 11.1)	9.77 (8.66 - 11.0)	0.98
CP-Fluorescein	13.2 (10.5 - 16.5)	3.65 (3.18 - 4.18)	3.62
CP-SiR	61.9 (52.5 - 72.7)	20.9 (19.5 - 22.4)	2.96
CP-TMR	5.64 (5.04 - 6.30)	3.37 (3.08 - 3.70)	1.67

Figure S13: Comparison of fluorophore substrate affinities between the dead mutants SNAP^{C145A} and SNAPf^{C145A}.

A. Titration curves obtained for the dead mutants SNAP^{C145A} and SNAPf^{C145A} measured via fluorescence polarization. The FP value of each dye fully bound to native SNAP/SNAPf was added at c = 0.005 M to improve fitting of the upper plateau. (See corresponding methods section for more details). **B**. Table summarizing fitted K_d values with 95% confidence intervals. For structures of BG and CP substrates see **Fig. S1.**

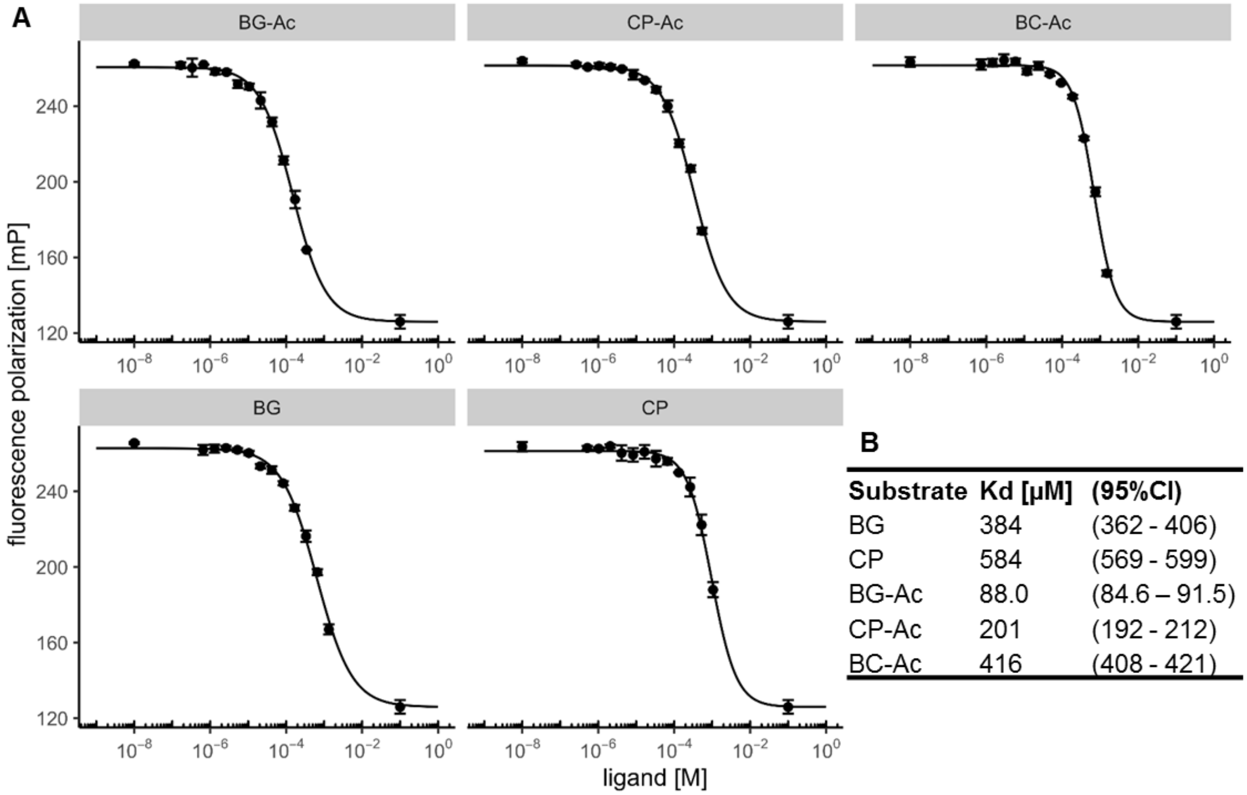

Substrate	Kd [µM]	(95%CI)
BG	384	(362 - 406)
CP	584	(569 - 599)
BG-Ac	88.0	(84.6 – 91.5)
CP-Ac	201	(192 - 212)
BC-Ac	416	(408 - 421)

Figure S14: Comparison of non-derivatized core substrate affinities with the dead mutant SNAP^{C145}.

A. Titration curves obtained for the dead mutant SNAP^{C145A} measured via competitive fluorescence polarization. The FP value of free dye was added at c = 0.1 M to improve fitting of the lower plateau. (See corresponding methods section for more details) **B**. Table summarizing fitted K_d values with 95% confidence intervals. For structures of substrates see **Fig. S1.**

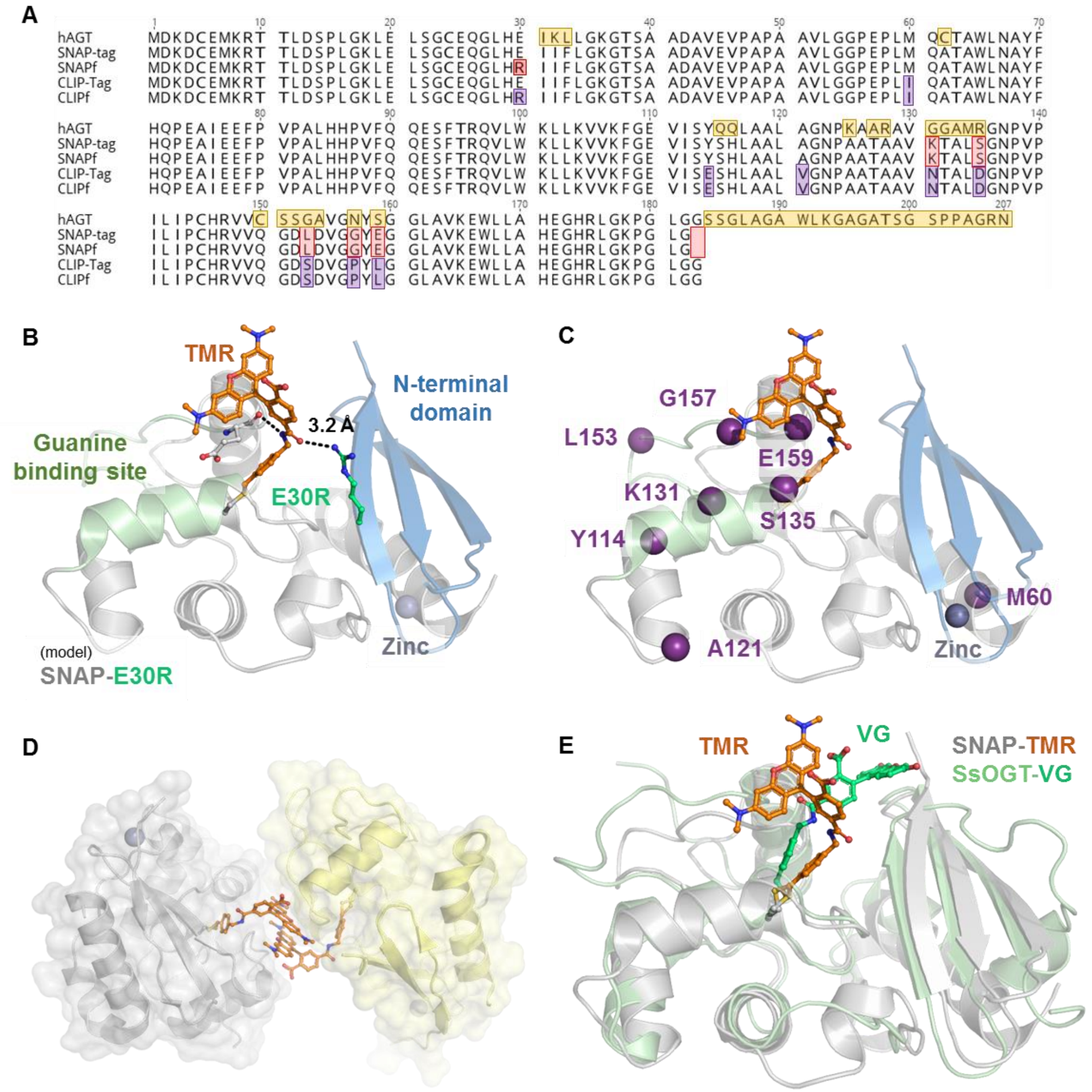

Figure S15: Sequence and additional structural information related to SNAP.

A. Sequence alignment of hAGT, SNAP, SNAPf, CLIP and CLIPf. Differences are highlighted in yellow, red and violet in the hAGT, SNAP(f) and CLIP(f) sequences, respectively. **B.** Modeling of the E30R mutation in the SNAP-TMR crystal structure. SNAP is represented as cartoon, the fluorophore substrate and residues as sticks. Putative hydrogen bonds and corresponding distances are indicated by black dashes. **C.** Crystal structure of SNAP labeled with TMR with α-carbons of the residues that differ between SNAP and CLIP represented as purple spheres. **D.** Benzyl-TMR constraints by the crystal packing. Two monomers of SNAP-TMR are represented as grey and yellow cartoons. The conformation of the benzyl-TMR (orange sticks) of both monomers is constrained by the other monomer. Symmetry mates were generated within 4 Å radius and selected to highlight the packing constraints. **E.** Structural alignment of SNAP-TMR with *Ss*OGT-H^{5}-VG structure (PDB ID 6GA0) [17].

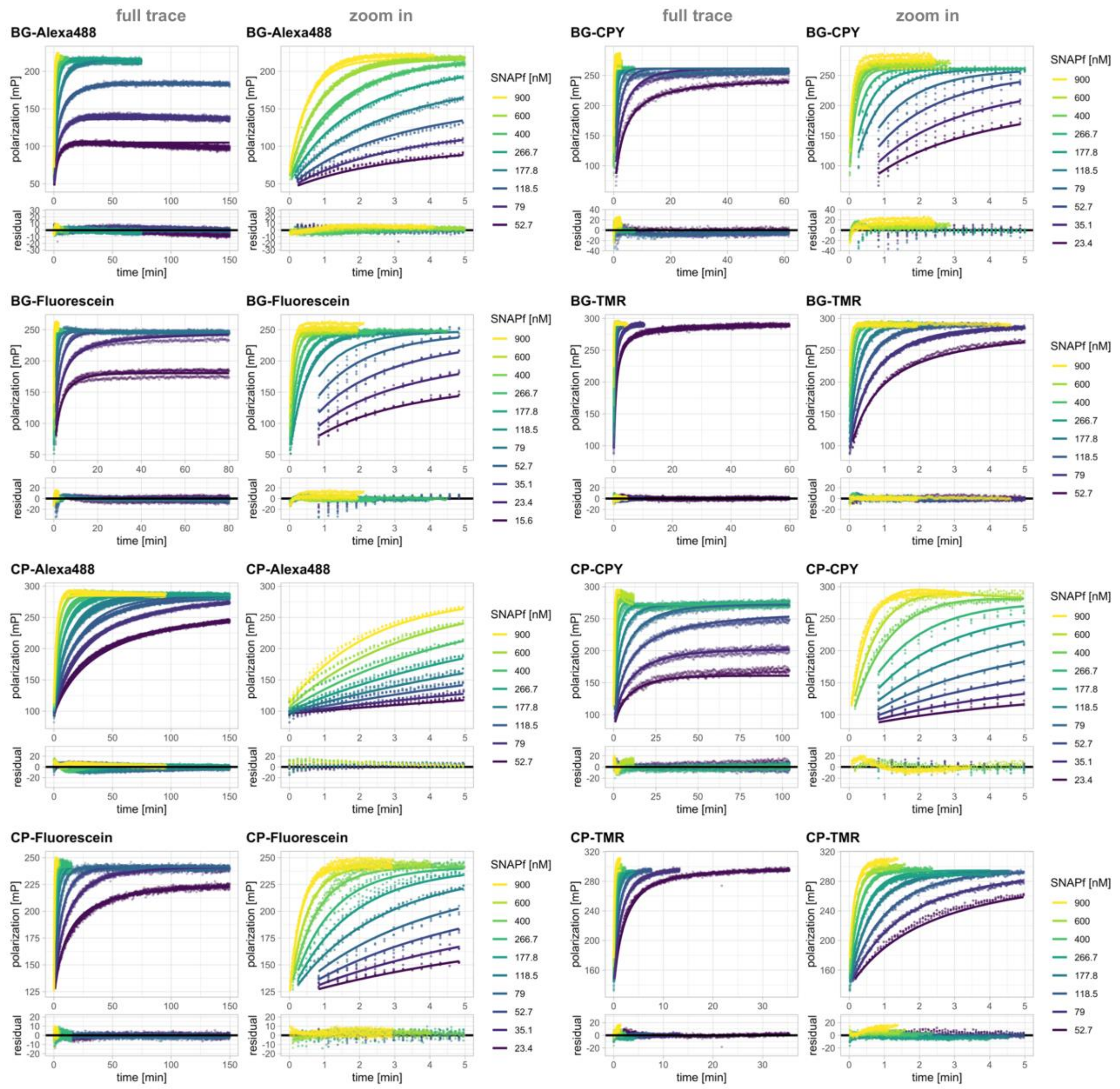

Figure S16: Labeling kinetics of SNAPf with fluorescent BG and CP substrates.

Full fluorescence polarization traces (points) and predications of fits based on model 1 or 1.2 (lines) along with zoom on the initial 5 minutes are represented on the top panels. All substrates were fitted to model 1 except CP-CPY, which showed an additional phase (model 1.2). Residuals from the fits are depicted in the bottom panels. Kinetics were recorded by following fluorescence polarization changes over time using a plate reader. Labeling was performed at different concentrations of SNAPf protein. Substrate concentrations were aimed at 20 nM based on the dyes extinction coefficient but fitted in the model since significant deviations from the expected stoichiometry were observed. For structures of BG and CP substrates see **Fig. S1.**

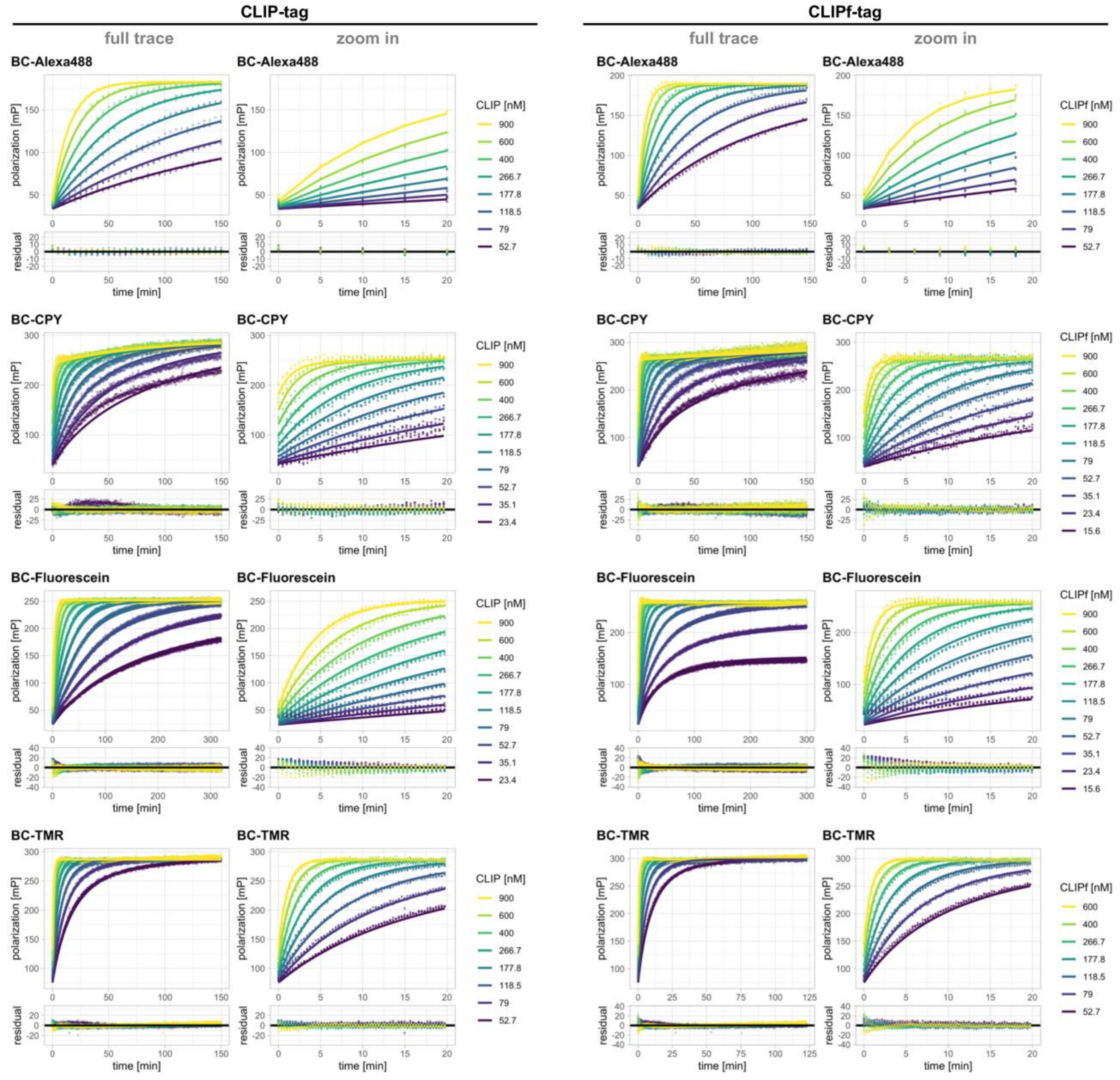

Figure S17: Labeling kinetics of CLIP and CLIPf with fluorescent BC substrates.

Full fluorescence polarization traces (points) and predications of fits based on model 1 or 1.2 (lines) along with zoom on the initial 20 minutes are represented on the top panels. All substrates were fitted to model 1 except BC-CPY, which showed an additional phase (model 1.2). Residuals from the fits are depicted in the bottom panels. Kinetics were recorded by following fluorescence polarization changes over time using a plate reader. Labeling was performed at different concentrations of CLIP and CLIPf protein. Substrate concentrations were aimed at 20 nM based on the dyes extinction coefficient but fitted in the model since significant deviations from the expected stoichiometry were observed. For structures of BC substrates see **Fig. S1**.

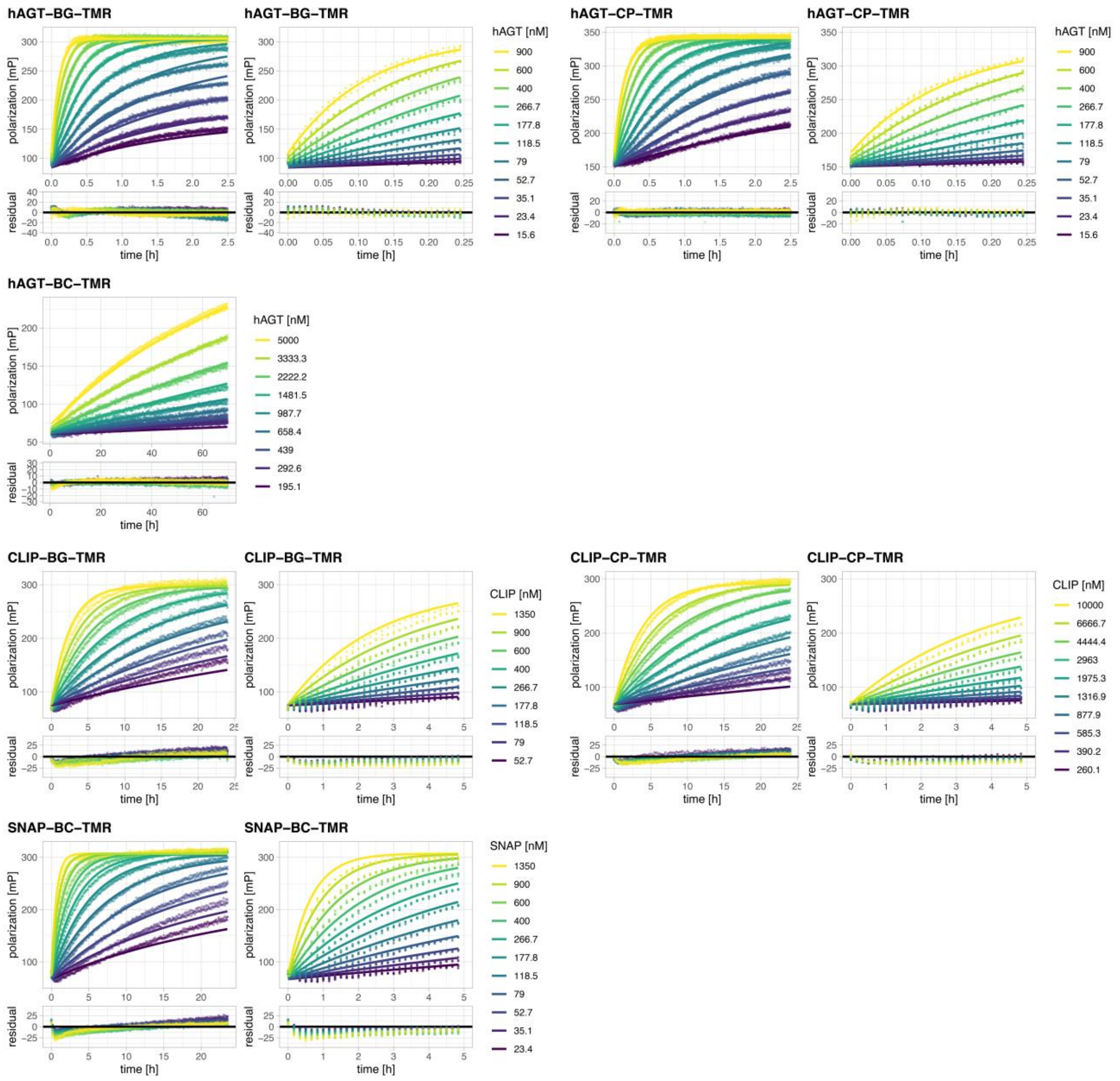

Figure S18: Labeling kinetics of hAGT, SNAP and CLIP with the non-respective BG-, CP- and BC-TMR substrates.

Full fluorescence polarization traces (points) and predications of fits based on model 1 along with zoom on the initial part (except for BC-TMR and hAGT) are represented on the top panels. Residuals from the fits are depicted in the bottom panels. Kinetics were recorded by following fluorescence polarization changes over time using a plate reader. Labeling was performed at different concentrations of hAGT, SNAP and CLIP proteins. Substrate concentrations were aimed at 20 nM based on the dyes extinction coefficient but fitted in the model since significant deviations from the expected stoichiometry were observed. For structures of BG, CP and BC substrates see **Fig. S1**.

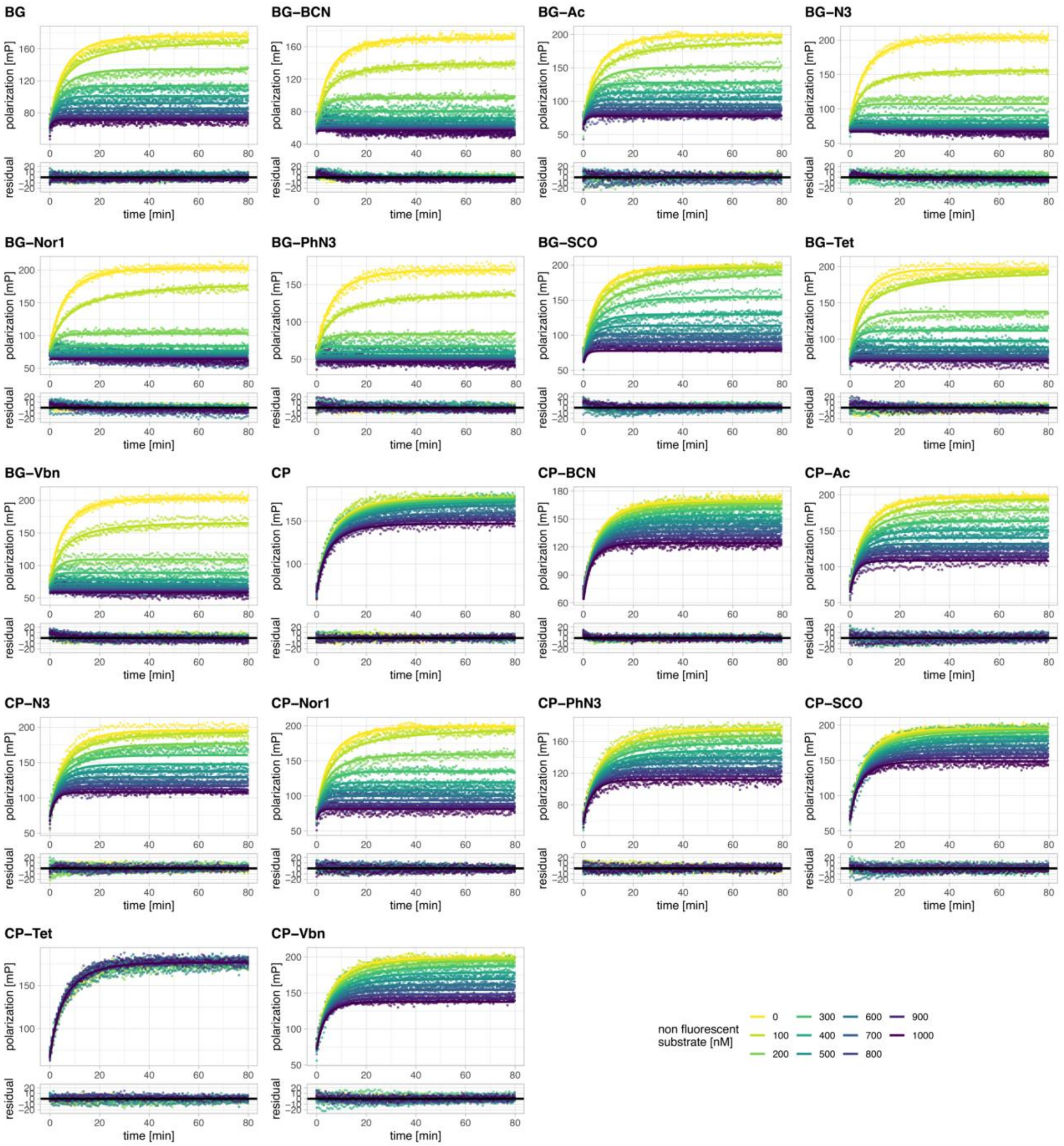

Figure S19: Labeling kinetics of SNAP with non-fluorescent BG and CP substrates.

Fluorescence polarization traces (points) of kinetic competition assays and predications of fits based on a simple competitive model (lines, see methods section for details) of SNAP labeling with BG-Alexa488 in the presence of different concentrations of non-fluorescent BG/CP substrates are represented on the top panels. Residuals from the fits are depicted in the bottom panels. Kinetics were recorded by following fluorescence polarization changes over time using a plate reader. For structures of BG and CP substrates see **Fig. S1.**

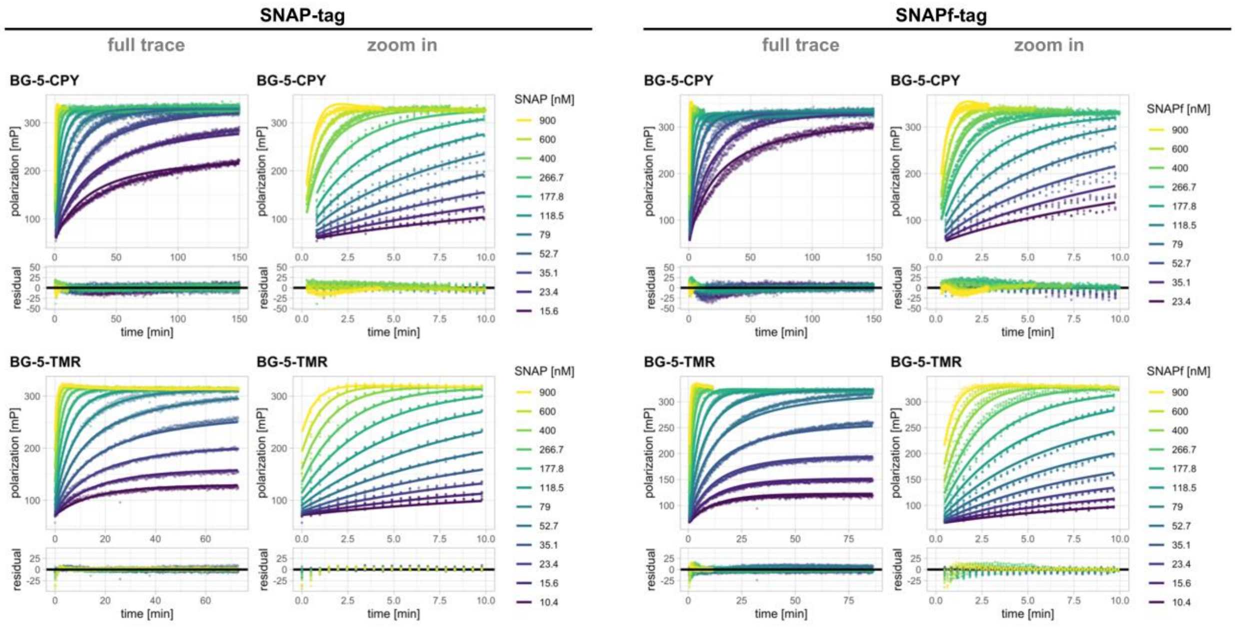

Figure S20: Labeling kinetics of SNAP and SNAPf with BG-5-TMR and BG-5-CPY.

Full fluorescence polarization traces (points) and predications of fits based on model 1.2 (lines) along with zoom on the initial 10 minutes are represented on the top panels. Residuals from the fits are depicted in the bottom panels. Kinetics were recorded by following fluorescence polarization changes over time using a plate reader. Labeling was performed at different concentrations of SNAP and SNAPf protein. Substrate concentrations were aimed at 20 nM based on the dyes extinction coefficient but fitted in the model since significant deviations from the expected stoichiometry were observed. For structures of BG substrates see **Fig. S1**.

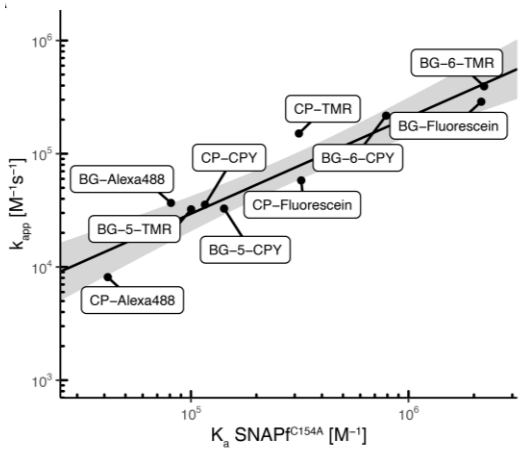

Figure S21: SNAPf kinetic and affinity correlations.

Correlation between SNAPf labeling kinetics (k_{app}) and affinity ($K_a = 1/K_d$) for different fluorophore substrates. Affinities were obtained with the catalytically dead mutant SNAPf^{C145A}. Log transformed values were fitted to a linear model (black line, $\log(k_{app}) = 0.2568 + \log(K_a) * 1.0697$, 95% confidence bands in grey, depicting the area in which the true regression line lies with 95% confidence). The linear correlation in logarithmic space suggests that the K_d of fluorescent SNAP substrates towards SNAPf^{C145A} could represent a valid proxy to estimate their K_{app} towards native SNAPf.

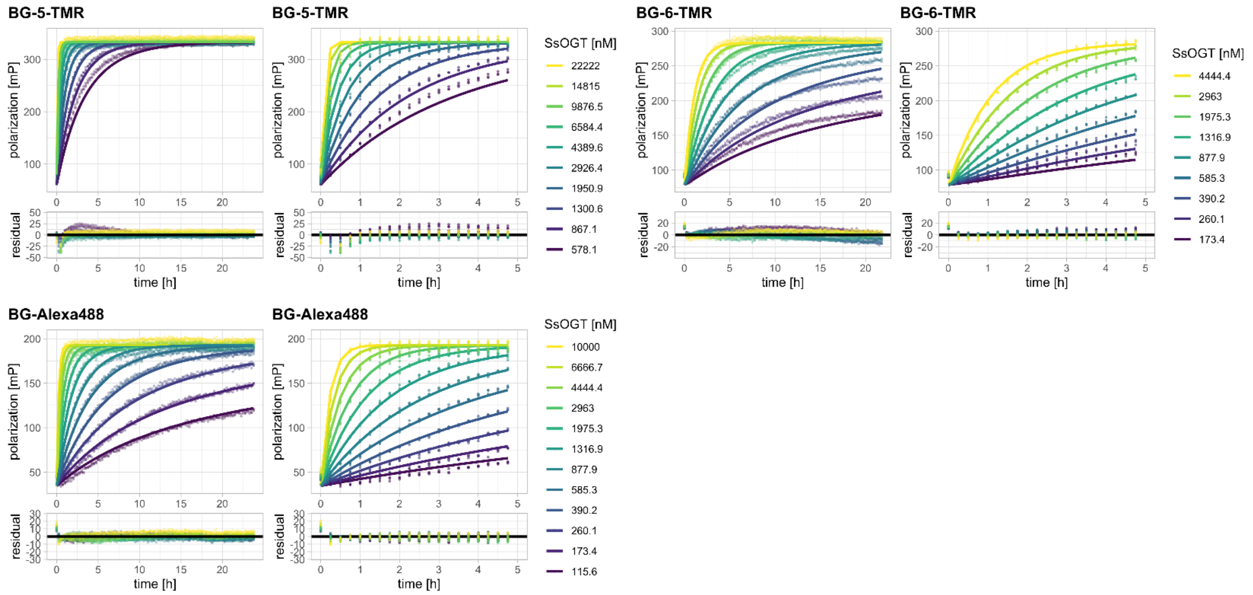

Figure S22: Labeling kinetics of *Ss*OGT-H[5] with BG-Alexa488 and BG-TMR.

Full fluorescence polarization traces (points) and predications of fits based on model 1 (lines) along with zoom on the initial 5 hours are represented on the top panels. Residuals from the fits are depicted in the bottom panels. Kinetics were recorded by following fluorescence polarization changes over time using a plate reader. Labeling was performed at different concentrations of *Ss*OGT-H[5] protein. Substrate concentrations were aimed at 20 nM based on the dyes extinction coefficient but fitted in the model since significant deviations from the expected stoichiometry were observed. For structures of BG substrates see **Fig. S1**.

Table S1: Data collection and refinement statistics the X-ray crystal structures.

**Data collections**	**SNAP-TMR**	**HT7-TMR**	**HT7-CPY**	**HOB-TMR**
**PDB ID**	6Y8P	6Y7A	6Y7B	6ZCC
**Beamline**	ESRF ID29	PXII-X10SA, SLS	PXII-X10SA, SLS	PXII-X10SA, SLS
**Wavelength (A°)**	0.976	1.00001	1.00006	0.99984
**Resolution (A°)**<br>**(last bin)**	36.88 - 2.3<br>(2.382 - 2.3)	50-1.40<br>(1.50-1.40)	50-3.10<br>(3.20-3.10)	50-1.50<br>(1.60-1.50)
**Space group**	$P3_2\,21$	$P12_11$	$P321$	$P2_12_12_1$
		**Unit cell dimensions**		
**a (A°)**	65.5148	44.00	161.27	52.21
**b (A°)**	65.5148	78.14	161.27	64.77
**c (A°)**	97.067	45.24	124.66	78.85
**No. observed reflections**	119190 (12210)	160637 (29978)	231609 (21528)	228695 (8515)
**No. unique reflections**	11152 (1087)	50448 (9451)	34294 (3081)	38699 (3579)
**Completeness (%)**	99.94 (100.00)	96.5 (97.1)	99.8 (99.9)	88.9 (47.5)
**Rmerge**	0.1015 (0.8636)	0.063 (0.410)	0.196 (0.596)	0.042 (0.241)
**I/σ(I)**	13.48 (2.82)	9.59 (2.83)	8.53 (3.10)	18.87 (2.39)
**CC ½ (%)**	99.9 (19.3)	99.7 (86.4)	98.8 (85.7)	99.9 (93.4)
**Redundancy**	10.7 (11.2)	3.18 (3.17)	6.75 (6.99)	5.91 (2.38)
**Wilson B**	47.75	21.39	37.99	32.28
		**Refinement statistics**		
**Resolution range (A)**	36.88-2.3	39.19-1.40	49.32-3.10	43.53-1.52
**No. Reflections**	8878	50435	34290	38697
**Rwork (%)**	0.2385	0.1558	0.2074	0.1887
**Rfree (%)**	0.2694	0.1868	0.2594	0.2238
**No. protein atoms**	1231	2397	11750	2348
**No. water atoms**	50	348	0	312
**No. ligand atoms**	45	51	235	52
**Average B factor (A°2)**	73.06	18.93	51.23	31.74
		**RMSD from ideal**		
**Bond lengths (A°)**	0.007	0.013	0.004	0.009
**Bond angles (°)**	1.24	1.247	0.788	1.014

Table S2: Kinetic parameters of HT7 labeling with fluorescent CA substrates.

Substrate	k_1 (± S.D.) [M^{-1} s^{-1}]	k_{-1} (± S.D.) [s^{-1}]	k_2 (± S.D.) [s^{-1}]	k_{app} (± S.D.) [M^{-1} s^{-1}]
CA-TMR	7.84 (± 0.76) × 10^7	2.56 (± 0.38) × 10^1	8.06 (± 0.29)	1.88 (± 0.01) × 10^7
CA-JF549	1.60 (± 0.16) × 10^8	9.83 (± 2.04) × 10^1	1.82 (± 0.07) × 10^1	1.66 (± 0.01) × 10^7
CA-JF669	2.35 (± 0.75) × 10^7	3.39 (± 1.49) × 10^1	6.94 (± 0.47)	4.03 (± 0.02) × 10^6
CA-CPY	1.6.7 (± 0.067) × 10^8	7.60 (± 0.98)	9.86 (± 0.73)	9.44 (± 0.18) × 10^7
CA-LIVE580	1.74 (± 0.05) × 10^8	1.75 (± 0.28)	6.77 (± 0.77)	1.39 (± 0.03) × 10^8
CA-TMR-biotin	3.69 (± 0.25) × 10^7	2.10 (± 0.25) × 10^1	8.24 (± 0.28)	1.04 (± 0.01) × 10^7

Data analyzed using model 2.

Table S3: Comparison k_{app} of HT7 labeling kinetics analyzed using models 1 and 2.

Substrate	k_{app} (± S.D.) [M^{-1} s^{-1}]	
	**Model 1**	**Model 2**
CA-TMR	1.79 (± 0.01) × 10^7	1.88 (± 0.01) × 10^7
CA-JF549	1.46 (± 0.01) × 10^7	1.66 (± 0.01) × 10^7
CA-JF669	3.95 (± 0.02) × 10^6	4.03 (± 0.02) × 10^6
CA-CPY	1.10 (± 0.02) × 10^8	9.44 (± 0.18) × 10^7
CA-LIVE580	1.58 (± 0.02) × 10^8	1.39 (± 0.03) × 10^8
CA-TMR-biotin	9.00 (± 0.04) × 10^6	1.04 (± 0.01) × 10^7

Table S4: Comparison of HT7 and HOB labeling kinetics with fluorescent CA substrates.

Protein	Substrate	k_1 (± S.D.) [M^{-1} s^{-1}]	k_{-1} (± S.D.) [s^{-1}]	k_2 (± S.D.) [s^{-1}]	k_{app} (± S.D.) [M^{-1} s^{-1}]
**HT7**	CA-TMR	7.84 (± 0.76) × 10^7	2.56 (± 0.38) × 10^1	8.06 (± 0.29)	1.88 (± 0.01) × 10^7
	CA-Alexa488	-	-	-	2.57 (± 0.01) × 10^4
**HOB**	CA-TMR	4.15 (± 0.26) × 10^7	1.83 (± 0.17) × 10^1	5.05 (± 0.13)	8.99 (± 0.04) × 10^6
	CA-Alexa488	-	-	-	8.04 (± 0.02) × 10^4

Table S5: Kinetic parameters of SNAP and CLIP labeling with fluorescent substrates analyzed using model 1.2.

Substrate	k_{app} (± S.D.) [$s^{-1}M^{-1}$]	k_3 (± S.D.) [s^{-1}]	k_{app} (± S.D.) [$s^{-1}M^{-1}$]	k_3 (± S.D.) [s^{-1}]
SNAP CP-Fluorescein	1.42 (± 0.01) × 10^4	1.61 (± 0.04) × 10^{-3}	-	-
SNAP CP-CPY	1.59 (± 0.01) × 10^4	1.26 (± 0.01) × 10^{-2}	3.55 (± 0.02) × 10^4	6.22 (± 0.13) × 10^{-3}
CLIP BC-CPY	1.26 (± 0.01) × 10^4	2.16 (± 0.09) × 10^{-4}	2.65 (± 0.01) × 10^4	9.02 (± 0.48) × 10^{-7}

Table S6: Kinetic parameters of SNAP labeling with TMR substrates measured via stopped flow.

Substrate	k_1 (± S.D.) [M^{-1} s^{-1}]	k_{-1} (± S.D.) [s^{-1}]	k_2 (± S.D.) [s^{-1}]	k_{app} (± S.D.) [M^{-1} s^{-1}]
BG-TMR	4.93 (± 0.04) × 10^5	1.02 (± 0.03)	1.24 (± 0.02)	2.71 (± 0.01) × 10^5
CP-TMR	5.36 (± 0.30) × 10^5	8.96 (± 0.71)	1.58 (± 0.04)	0.81 (± 0.01) × 10^5

Data analyzed using model 2

Table S7: Comparison of SNAP/CLIP with SNAPf/CLIPf labeling kinetics with fluorescent substrates.

Substrate		k_{app} (± S.D.) [$s^{-1}M^{-1}$]	
		Original	Fast variant
SNAP BG substrates	BG-Alexa488	1.22 (± 0.01) × 10^4	3.68 (± 0.64) × 10^4
	BG-Fluorescein	1.17 (± 0.01) × 10^5	2.88 (± 0.01) × 10^5
	BG-CPY	2.17 (± 0.01) × 10^5	2.17 (± 0.02) × 10^5
	BG-TMR	4.29 (± 0.01) × 10^5	3.94 (± 0.01) × 10^5
SNAP CP substrates	CP-Alexa488	3.12 (± 0.003) × 10^3	8.13 (± 0.01) × 10^3
	CP-Fluorescein	1.42 (± 0.01) × 10^4 (*)	5.81 (± 0.01) × 10^4
	CP-CPY*	1.59 (± 0.01) × 10^4 (*)	3.55 (± 0.02) × 10^4 (*)
	CP-TMR	7.69 (± 0.01) × 10^4	1.51 (± 0.01) × 10^5
CLIP BC substrates	BC-Alexa488	1.26 (± 0.01) × 10^3	3.10 (±0.02) × 10^3
	BC-Fluorescein	4.36 (± 0.01) × 10^3	1.62 (± 0.01) × 10^4
	BC-TMR	1.85 (± 0.01) × 10^4	3.37 (± 0.01) × 10^4
	BC-CPY*	1.26 (± 0.01) × 10^4 (*)	2.65 (± 0.01) × 10^4 (*)

Data analyzed using model 1 or 1.2 (*) which included an additional phase (see Table S5).

Table S8: Comparison of SNAP labeling kinetics with 5- and 6-fluorophores.

Substrate	SNAP		SNAPf	
	k_{app} (± S.D.) [$s^{-1}M^{-1}$]	k_3 (± S.D.) [s^{-1}]	k_{app} (± S.D.) [$s^{-1}M^{-1}$]	k_3 (± S.D.) [s^{-1}]
BG-6-TMR	4.29 (± 0.01) × 10^5	-	3.94 (± 0.01) × 10^5	-
BG-5-TMR (*)	2.67 (± 0.01) x 10^4	1.53 (± 0.12) x 10^{-3}	3.23 (± 0.01) x 10^4	2.18 (± 0.18) x 10^{-3}
BG-6-CPY	2.17 (± 0.01) × 10^5	-	2.17 (± 0.02) × 10^5	-
BG-5-CPY (*)	2.51(± 0.01) x 10^4	2.11 (± 0.04) x 10^{-2}	3.28 (± 0.01) x 10^4	1.42 (± 0.03) x 10^{-2}

Data analyzed using model 1 or 1.2 (*) which included an additional phase.

Table S9: Kinetic parameters of *Ss*OGT-H[5] labeling.

Substrate	k_{app} (± S.D.) [$s^{-1}M^{-1}$]
BG-6-TMR	6.78 (± 0.67) x 10^1
BG-5-TMR	1.45 (± 0.92) x 10^2
BG-6-Alexa488	1.24 (± 0.01) x 10^2

Data analyzed using model 1.

Protein sequences:

General color code: Hisx10-tag – TEV cleavage site – Protein sequence – **Fast mutation** – **Catalytic residue**

>HT7
MHHHHHHHHHHENLYFQ|GIGTGFPFDPHYVEVLGERMHYVDVGPRDGTPVLFLHGNPTSSYVWRNIIPHVAPTHRCIAPDLIGMGKS
DKPDLGYFFDDHVRFMDAFIEALGLEEVVLVIH**D**WGSALGFHWAKRNPERVKGIAFMEFIRPIPTWDEWPEFARETFQAFRTTDVGRK
LIIDQNVFIEGTLPMGVVRPLTEVEMDHYREPFLNPVDREPLWRFPNELPIAGEPANIVALVEEYMDWLHQSPVPKLLFWGTPGVLIPP
AEAARLAKSLPNCKAVDIGPGLNLLQEDNPDLIGSEIARWLSTLEI

>HOB
MHHHHHHHHHHENLYFQ|GIGTGFPFDPHYVEVLGERMHYVDVGPRDGTPVLFLHGNPTSSYVWRNIIPHVAPTHRCIAPDLIGMGKS
DKPDLGYFFDDHVRFMDAFIEALGLEEVVLVIH**D**WGSALGFHWAKRNPERVKGIAFMEFIRPIPTWDEWP**K**FAR**K**TFQAFRT**KK**VGR
KLIIDQNVFIEGTLPMGVVRPLTEVEMDHYREPFLNPVDREPLWRFPNELPIAGEPANIVALVEEYMDWLHQSPVPKLLFWGTPGVLIP
PAEAARLAKSLPNCKAVDIGPGLNLLQEDNPDLIGSEIARWLSTLEISG
Color code: **mutations as compared to HT7**

>SNAP
MHHHHHHHHHHENLYFQ|GMDKDCEMKRTTLDSPLGKLELSGCEQGLHEIIFLGKGTSAADAVEVPAPAAVLGGPEPLMQATAWLNA
YFHQPEAIEEFPVPALHHPVFQQESFTRQVLWKLLKVVKFGEVISYSHLAALAGNPAATAAVKTALSGNPVPILIP**C**HRVVQGDLDVGG
YEGGLAVKEWLLAHEGHRLGKPGLG

>SNAPf
MHHHHHHHHHHENLYFQ|GMDKDCEMKRTTLDSPLGKLELSGCEQGLH**R**IIFLGKGTSAADAVEVPAPAAVLGGPEPLMQATAWLNA
YFHQPEAIEEFPVPALHHPVFQQESFTRQVLWKLLKVVKFGEVISYSHLAALAGNPAATAAVKTALSGNPVPILIP**C**HRVVQGDLDVGG
YEGGLAVKEWLLAHEGHRLGKPGLG

>SNAP^{cx}
MHHHHHHHHHHENLYFQ|GDCEMKRTTLDSPLGKLELSGCEQGLHEIIFLGKGTSAADAVEVPAPAAVLGGPEPLMQATAWLNAYFH
QPEAIEEFPVPALHHPVFQQESFTRQVLWKLLKVVKFGEVISYSHLAALAGNPAATAAVKTALSGNPVPILIP**C**HRVVQGDLDVGGYEG
GLAVKEWLLAHEGHRLGKR

>CLIP
MHHHHHHHHHHENLYFQ|GMDKDCEMKRTTLDSPLGKLELSGCEQGLHEIIFLGKGTSAADAVEVPAPAAVLGGPEPLIQATAWLNAY
FHQPEAIEEFPVPALHHPVFQQESFTRQVLWKLLKVVKFGEVISESHLAALVGNPAATAAVNTALDGNPVPILIP**C**HRVVQGDSDVGPY
LGGLAVKEWLLAHEGHRLGKPGLGG

>CLIPf
MHHHHHHHHHHENLYFQ|GMDKDCEMKRTTLDSPLGKLELSGCEQGLH**R**IIFLGKGTSAADAVEVPAPAAVLGGPEPLIQATAWLNA
YFHQPEAIEEFPVPALHHPVFQQESFTRQVLWKLLKVVKFGEVISESHLAALVGNPAATAAVNTALDGNPVPILIP**C**HRVVQGDSDVGP
YLGGLAVKEWLLAHEGHRLGKPGLGG

>hAGT
*MAS*WSHPQFEK*GA*DDDDK*VPH*MDKDCEMKRTTLDSPLGKLELSGCEQGLHEIKLLGKGTSAADAVEVPAPAAVLGGPEPLMQCTA
WLNAYFHQPEAIEEFPVPALHHPVFQQESFTRQVLWKLLKVVKFGEVISYQQLAALAGNPKAARAVGGAMRGNPVPILIPCHRVVCSS
GAVGNYSGGLAVKEWLLAHEGHRLGKPGLGGSSGLAGAWLKGAGATSGSPPAGRN*APGFSSISA*HHHHHHHHHH
Color code: Strep-Tag II, Enterokinase cleavage site, *linkers*

>*Ss*OGT-H^{5}

*MAS*WSHPQFEK*GA*DDDDK*VPH*MLVYGLYKSPLGYITVAKDDKGFIMLDFCDCVEGNSRDDSSFTEFFHKLDLYFEGKPINLREPINLKTYPFRLSVFKEVMKIPWGKVMTYKQIADSLGTAPAAVKTALSENPILLIIPCHRVIAENGIGGYERGVKLKRALLELEGVKIPEL*APGFSSISA*HHHHHHHHHH

Color code: Strep-Tag II, Enterokinase cleavage site, *linkers*

Example DynaFit scripts:

HT7 stopped flow labeling kinetics model 2

```
[task]
   data  = progress
   task  = fit
   confidence = monte-carlo

[mechanism]
   P + S <===> P.S      :   k1   k-1
   P.S ----> Z          :   k2

[constants] ; units: uM, sec
   k1   = 10 ?
   k-1  = 10 ?
   k2   = 10 ?

[parameters]
   R    = 0.2 ?

[data]
   Delay      0.022
   offset     0.0262
   directory  path/to/data
   sheet      data.csv
   column 6  | conc P =   1     | conc S =   1     | response Z = 1     * R  |
response P.S = 1     * R |  label c=1
   column 5  | conc P =   0.75  | conc S =   0.75  | response Z = 1.333 * R  |
response P.S = 1.333 * R |  label c=0.75
   column 4  | conc P =   0.5   | conc S =   0.5   | response Z = 2     * R  |
response P.S = 2     * R |  label c=0.5
   column 3  | conc P =   0.25  | conc S =   0.25  | response Z = 4     * R  |
response P.S = 4     * R |  label c=0.25
   column 2  | conc P =   0.1   | conc S =   0.1   | response Z = 10    * R  |
response P.S = 10    * R |  label c=0.1

[output]
   directory path/to/output/folder

[settings]
   {ConfidenceIntervals}
      LevelPercent = 95
   {Output}
      XAxisLabel = time [s]
      YAxisLabel = anisotropy

[end]
```

SNAP stopped flow labeling kinetics model 2

```
[task]
   data  = progress
   task  = fit
   confidence = monte-carlo

[mechanism]
   P + S <===> P.S      :   k1   k-1
   P.S ----> Z          :   k2

[constants] ; units: uM, sec
   k1   = 1 ?
   k-1  = 1 ?
   k2   = 1 ?

[concentrations]  ; units: uM
   S    = 2 ?

[responses]
   Z    = 0.07 ?
   P.S  = 1 * Z

[data]
   delay      0.022
   offset     0
   directory  path/to/data
   sheet      data.csv
   column  2  | conc P =   50     | label c=50
   column  3  | conc P =   37.5   | label c=37.5
   column  4  | conc P =   25     | label c=25
   column  5  | conc P =   12.5   | label c=12.5
   column  6  | conc P =   5      | label c=5
   column  7  | conc P =   2.5    | label c=2.5
   column  8  | conc P =   1.25   | label c=1.25

[output]
   directory  path/to/output/folder

[settings]
   {ConfidenceIntervals}
      LevelPercent  = 95
   {Output}
      XAxisLabel = time [s]
      YAxisLabel = anisotropy

[end]
```

HT7 microplate reader labeling kinetics model 1

(Time series of each condition were not averaged before DynaFit analysis since the TECAN plate reader has small inconsistencies in measurement intervals)

```
[task]
   data  = progress
   task  = fit
   confidence = monte-carlo

[mechanism]
   P + S ----> Z     :   k_app

[constants] ; units: uM, sec
   k_app = 1 ?

[concentrations]  ; units: uM
   S   = 0.05

[responses]
   Z   = 4000 ?

[data]
   delay      1
   offset     87.118
   directory  path/to/data
   sheet      data.csv

   column  2 |  conc P =  0     |  label 0
   column  3 |  conc P =  0     |  label 0
   column  4 |  conc P =  0     |  label 0

   column  5 |  conc P =  0.4   |  label 400
   column  6 |  conc P =  0.4   |  label 400
   column  7 |  conc P =  0.4   |  label 400

   column  8 |  conc P =  0.8   |  label 800
   column  9 |  conc P =  0.8   |  label 800
   column 10 |  conc P =  0.8   |  label 800

   column 11 |  conc P =  1.6   |  label 1600
   column 12 |  conc P =  1.6   |  label 1600
   column 13 |  conc P =  1.6   |  label 1600

   column 14 |  conc P =  3.2   |  label 3200
   column 15 |  conc P =  3.2   |  label 3200
   column 16 |  conc P =  3.2   |  label 3200

   column 17 |  conc P =  6.4   |  label 6400
   column 18 |  conc P =  6.4   |  label 6400
   column 19 |  conc P =  6.4   |  label 6400

   column 20 |  conc P =  12.8  |  label 12800
   column 21 |  conc P =  12.8  |  label 12800
   column 22 |  conc P =  12.8  |  label 12800

   column 23 |  conc P =  25.6  |  label 25600
   column 24 |  conc P =  25.6  |  label 25600
   column 25 |  conc P =  25.6  |  label 25600

   column 26 |  conc P =  51.2  |  label 51200
   column 27 |  conc P =  51.2  |  label 51200
   column 28 |  conc P =  51.2  |  label 51200

[output]
   directory path/to/output/folder

[settings]
   {ConfidenceIntervals}
      LevelPercent = 95
   {Output}
      XAxisLabel = time [s]
      YAxisLabel = anisotropy

[end]
```

SNAP-/CLIP microplate reader labeling kinetics model 1

(Time series of each condition were not averaged before DynaFit analysis since the TECAN plate reader has small inconsistencies in measurement intervals)

```
[task]
   data  = progress
   task  = fit
   confidence = monte-carlo

[mechanism]
   P + S ----> Z        :  k_app

[constants]  ; units: nM, sec
   k_app = 0.0001  ?

[concentrations]  ; units: nM
   S = 50  ?

[responses]
   Z = 2  ?

[data]
   delay      2.7
   offset     73.46
   directory  path/to/data
   sheet      data.csv

   column  2  | conc  P =  52      | label 52
   column  3  | conc  P =  52      | label 52
   column  4  | conc  P =  52      | label 52

   column  5  | conc  P =  79      | label 79
   column  6  | conc  P =  79      | label 79
   column  7  | conc  P =  79      | label 79

   column  8  | conc  P =  118.5   | label 118.5
   column  9  | conc  P =  118.5   | label 118.5
   column 10  | conc  P =  118.5   | label 118.5

   column 11  | conc  P =  177.7   | label 177.7
   column 12  | conc  P =  177.7   | label 177.7
   column 13  | conc  P =  177.7   | label 177.7

   column 14  | conc  P =  266.6   | label 266.6
   column 15  | conc  P =  266.6   | label 266.6
   column 16  | conc  P =  266.6   | label 266.6

   column 17  | conc  P =  400     | label 400
   column 18  | conc  P =  400     | label 400
   column 19  | conc  P =  400     | label 400

   column 20  | conc  P =  600     | label 600
   column 21  | conc  P =  600     | label 600
   column 22  | conc  P =  600     | label 600

   column 23  | conc  P =  900     | label 900
   column 24  | conc  P =  900     | label 900
   column 25  | conc  P =  900     | label 900

[output]
  directory  path/to/output/folder

[settings]
   {ConfidenceIntervals}
      LevelPercent  = 95
   {Output}
      XAxisLabel = time [s]
      YAxisLabel = anisotropy

[end]
```

SNAP-/CLIP microplate reader labeling kinetics model 1.2

(Time series of each condition were not averaged before DynaFit analysis since the TECAN plate reader has small inconsistencies in measurement intervals)

```
[task]
   data  = progress
   task  = fit
   confidence = monte-carlo

[mechanism]
   P + S ----> Z        :  k_app
   Z     ----> Z2       :  k_app_2

[constants] ; units: nM, sec
   k_app   = 0.0001 ?
   k_app_2 = 0.0001 ?

[concentrations]  ; units: nM
   S = 50  ?

[responses]
   Z  = 2 ?
   Z2 = 2 ?

[data]
   delay      2.7
   offset     73.46
   directory  path/to/data
   sheet      data.csv

   column  2  | conc  P =  52     | label 52
   column  3  | conc  P =  52     | label 52
   column  4  | conc  P =  52     | label 52

   column  5  | conc  P =  79     | label 79
   column  6  | conc  P =  79     | label 79
   column  7  | conc  P =  79     | label 79

   column  8  | conc  P =  118.5  | label 118.5
   column  9  | conc  P =  118.5  | label 118.5
   column 10  | conc  P =  118.5  | label 118.5

   column 11  | conc  P =  177.7  | label 177.7
   column 12  | conc  P =  177.7  | label 177.7
   column 13  | conc  P =  177.7  | label 177.7

   column 14  | conc  P =  266.6  | label 266.6
   column 15  | conc  P =  266.6  | label 266.6
   column 16  | conc  P =  266.6  | label 266.6

   column 17  | conc  P =  400    | label 400
   column 18  | conc  P =  400    | label 400
   column 19  | conc  P =  400    | label 400

   column 20  | conc  P =  600    | label 600
   column 21  | conc  P =  600    | label 600
   column 22  | conc  P =  600    | label 600

   column 23  | conc  P =  900    | label 900
   column 24  | conc  P =  900    | label 900
   column 25  | conc  P =  900    | label 900

[output]
  directory  path/to/output/folder

[settings]
   {ConfidenceIntervals}
      LevelPercent  = 95
   {Output}
      XAxisLabel = time [s]
      YAxisLabel = anisotropy

[end]
```

References:

[1] Butkevich, A. N., Mitronova, G. Y., Sidenstein, S. C., Klocke, J. L., Kamin, D., Meineke, D. N., D'Este, E., Kraemer, P. T., Danzl, J. G., Belov, V. N., and Hell, S. W. (2016) Fluorescent Rhodamines and Fluorogenic Carbopyronines for Super-Resolution STED Microscopy in Living Cells, *Angew Chem Int Ed Engl 55*, 3290-3294.

[2] Mudd, G., Pi, I. P., Fethers, N., Dodd, P. G., Barbeau, O. R., and Auer, M. (2015) A general synthetic route to isomerically pure functionalized rhodamine dyes, *Methods and Applications in Fluorescence 3*.

[3] Ueno, Y., Jose, J., Loudet, A., Pérez-Bolívar, C. s., Anzenbacher, P., and Burgess, K. (2011) Encapsulated Energy-Transfer Cassettes with Extremely Well Resolved Fluorescent Outputs, *Journal of the American Chemical Society 133*, 51-55.

[4] Lukinavicius, G., Umezawa, K., Olivier, N., Honigmann, A., Yang, G., Plass, T., Mueller, V., Reymond, L., Correa, I. R., Jr., Luo, Z. G., Schultz, C., Lemke, E. A., Heppenstall, P., Eggeling, C., Manley, S., and Johnsson, K. (2013) A near-infrared fluorophore for live-cell super-resolution microscopy of cellular proteins, *Nat Chem 5*, 132-139.

[5] Wang, L., Tran, M., D'Este, E., Roberti, J., Koch, B., Xue, L., and Johnsson, K. (2020) A general strategy to develop cell permeable and fluorogenic probes for multicolour nanoscopy, *Nat Chem 12*, 165-172.

[6] Keppler, A., Gendreizig, S., Gronemeyer, T., Pick, H., Vogel, H., and Johnsson, K. (2003) A general method for the covalent labeling of fusion proteins with small molecules in vivo, *Nature biotechnology 21*, 86-89.

[7] Srikun, D., Albers, A. E., Nam, C. I., Iavarone, A. T., and Chang, C. J. (2010) Organelle-Targetable Fluorescent Probes for Imaging Hydrogen Peroxide in Living Cells via SNAP-Tag Protein Labeling, *Journal of the American Chemical Society 132*, 4455-4465.

[8] Keppler, A., Pick, H., Arrivoli, C., Vogel, H., and Johnsson, K. (2004) Labeling of fusion proteins with synthetic fluorophores in live cells, *Proceedings of the National Academy of Sciences 101*, 9955-9959.

[9] Correa, I., Baker, B., Zhang, A., Sun, L., Provost, C., Lukinavic.ius, G. z., Reymond, L., Johnsson, K., and Xu, M.-Q. (2013) Substrates for Improved Live-Cell Fluorescence Labeling of SNAP-tag, *Current Pharmaceutical Design 19*, 5414-5420.

[10] Hiblot, J., Yu, Q., Sabbadini, M. D. B., Reymond, L., Xue, L., Schena, A., Sallin, O., Hill, N., Griss, R., and Johnsson, K. (2017) Luciferases with Tunable Emission Wavelengths, *Angew Chem Int Ed Engl 56*, 14556-14560.

[11] Grimm, J. B., English, B. P., Chen, J., Slaughter, J. P., Zhang, Z., Revyakin, A., Patel, R., Macklin, J. J., Normanno, D., Singer, R. H., Lionnet, T., and Lavis, L. D. (2015) A general method to improve fluorophores for live-cell and single-molecule microscopy, *Nat Methods 12*, 244-250, 243 p following 250.

[12] Gautier, A., Juillerat, A., Heinis, C., Correa, I. R., Jr., Kindermann, M., Beaufils, F., and Johnsson, K. (2008) An engineered protein tag for multiprotein labeling in living cells, *Chem Biol 15*, 128-136.

[13] Bottanelli, F., Kromann, E. B., Allgeyer, E. S., Erdmann, R. S., Wood Baguley, S., Sirinakis, G., Schepartz, A., Baddeley, D., Toomre, D. K., Rothman, J. E., and Bewersdorf, J. (2016) Two-colour live-cell nanoscale imaging of intracellular targets, *Nature communications 7*.

[14] Zhang, Y., So, M.-k., Loening, A. M., Yao, H., Gambhir, S. S., and Rao, J. (2006) HaloTag Protein-Mediated Site-Specific Conjugation of Bioluminescent Proteins to Quantum Dots, *Angewandte Chemie International Edition 45*, 4936-4940.

[15] Masharina, A., Reymond, L., Maurel, D., Umezawa, K., and Johnsson, K. (2012) A fluorescent sensor for GABA and synthetic GABA(B) receptor ligands, *Journal of the American Chemical Society 134*, 19026-19034.

[16] Deo, C., Abdelfattah, A. S., Bhargava, H. K., Berro, A. J., Falco, N., Farrants, H., Moeyaert, B., Chupanova, M., Lavis, L. D., and Schreiter, E. R. (2021) The HaloTag as a general scaffold for far-red tunable chemigenetic indicators, *Nature chemical biology 17*, 718-723.
[17] Rossi, F., Morrone, C., Massarotti, A., Ferraris, D. M., Valenti, A., Perugino, G., and Miggiano, R. (2018) Crystal structure of a thermophilic O6-alkylguanine-DNA alkyltransferase-derived self-labeling protein-tag in covalent complex with a fluorescent probe, *Biochemical and Biophysical Research Communications 500*, 698-703.
